# Structural and functional insights into the evolution of SARS-CoV-2 KP.3.1.1 spike protein

**DOI:** 10.1016/j.celrep.2025.115941

**Published:** 2025-07-05

**Authors:** Ziqi Feng, Jiachen Huang, Sabyasachi Baboo, Jolene K. Diedrich, Sandhya Bangaru, James C. Paulson, John R. Yates, Meng Yuan, Ian A. Wilson, Andrew B. Ward

**Affiliations:** 1 Department of Integrative Structural and Computational Biology, The Scripps Research Institute, La Jolla, CA, USA; 2 Department of Molecular Medicine, The Scripps Research Institute, La Jolla, CA, USA; 3 Department of Immunology and Microbiology, The Scripps Research Institute, La Jolla, CA, USA; 4 The Skaggs Institute for Chemical Biology, The Scripps Research Institute, La Jolla, CA, USA; 5 Lead contact

## Abstract

The JN.1-sublineage KP.3.1.1 recently emerged as the globally prevalent SARS-CoV-2 variant, demonstrating increased infectivity and antibody escape. We investigate how mutations and a deletion in the KP.3.1.1 spike protein (S) affect hACE2 binding and antibody escape. Mass spectrometry confirms a new glycan site at residue N30 that alters the glycoforms at neighboring N61. Cryoelectron microscopy (cryo-EM) structures show that the N30 glycan and rearrangement of adjacent residues do not significantly change the overall spike structure, up-down ratio of receptor-binding domains (RBDs), or hACE2 binding. Furthermore, a KP.3.1.1 S with hACE2 structure further confirms an epistatic effect between F456L and Q493E on hACE2 binding. Our analysis shows that SARS-CoV-2 variants that emerged after late 2023 are now incorporating reversions to residues found in other sarbecoviruses, including the N30 glycan, Q493E, and others. Overall, these results inform on the structural and functional consequences of the KP.3.1.1 mutations, the current SARS-CoV-2 evolutionary trajectory, and immune evasion.

## In brief

Feng et al. determine high-resolution cryo-EM structures of KP.3.1.1 and other mutant spike proteins and find that an S31 deletion introduces N30 glycosylation. They demonstrate that S31 deletion and N30 glycosylation do not affect the spike conformation, hACE2 binding, and antibody RBD binding but do knock out some antibody NTD binding.

## Graphical Abstract

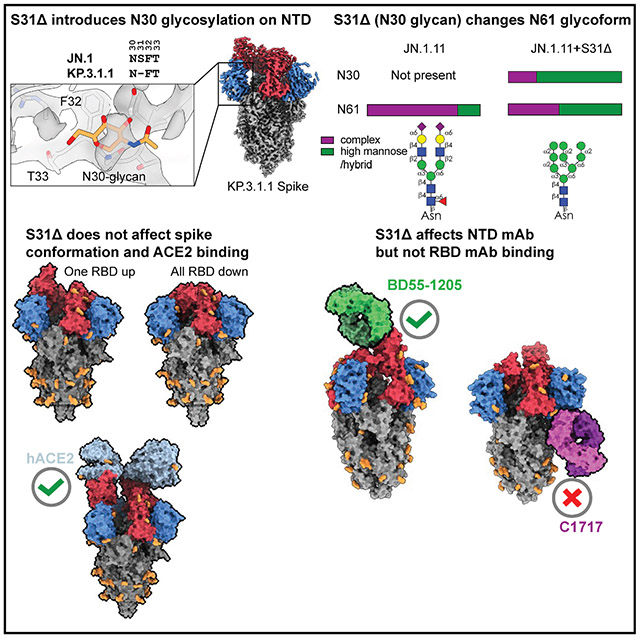

## INTRODUCTION

After 5 years of evolution in the human population, severe acute respiratory syndrome coronavirus 2 (SARS-CoV-2) continues to circulate globally, albeit with reduced severity and mortality. The continuous evolution has led to the emergence of a plethora of variants with multiple mutations, especially on the spike protein, allowing them to evade antibodies induced by prior infection or vaccination and/or enhance viral fitness and transmissibility. By the start of 2024, the JN.1 variant had outcompeted the previous XBB lineages.^1-4^ Subsequently, the JN.1-derived subvariants (e.g., KP.2 and KP.3) became the dominant strains.^5-7^ Recently, KP.3.1.1 emerged as the predominant SARS-CoV-2 variant circulating worldwide, overtaking its parent lineage KP.3 and earlier KP.2 variants.^8-11^ The proportion of KP.3.1.1 in the population continued to increase, and by September 2024, it accounted for more than 50% of global cases.^12,13^

KP.3.1.1 has acquired a higher relative effective reproduction number, higher infectivity, and greater neutralization escape than KP.3 and previous variants.^8-11,14,15^ Compared to JN.1, both KP.3 and KP.3.1.1 share two mutations, F456L and Q493E, in the receptor-binding domain (RBD) of the spike protein. These mutations work together to facilitate further antibody escape and exhibit better binding to the host receptor, human angiotensin-converting enzyme 2 (hACE2).^16,17^ An S31 deletion in the N-terminal domain (NTD) of KP.3.1.1 at ^30^NSFT^33^ introduces a potential N-linked glycosylation site (PNGS, NxS∣T motif) at N30 (NFT) that was previously observed in some non-SARS-CoV-2 sarbecoviruses. Previous studies have suggested that the S31 deletion and potential N30 glycosylation may alter the local spike conformation and enable the RBD to adopt a more “down” conformation, potentially enabling escape from certain classes of pre-existing neutralizing antibodies.^9,15^ A V1104L mutation, located on the lower stem of the spike S2, in KP.3.1.1 as well as in earlier subvariants (such as KP.2, KP.2.3, and KP.3) was considered to have a minimal effect on antibody evasion.^18^ Currently, little is known about the structural effects of the deletion and N30 glycosylation and the consequences and mechanism of immune escape in KP.3.1.1.

In this study, we used single-particle cryoelectron microscopy (cryo-EM) to investigate structures of the apo KP.3.1.1 spike and its complex with hACE2. By analyzing the molecular interactions between the KP.3.1.1 RBD and hACE2, we rationalized the epistatic and synergistic effects of F456L and Q493E mutations, which markedly enhanced the binding affinity to hACE2 over the Q493E mutation alone, similar to the F456L/Q493E synergy revealed previously revealed in the KP.3 variant.^17^ In addition, to dissect how the mutations S31Δ, F456L, and Q493E affect the overall spike stability, we introduced these mutations into the JN.1.11 spike protein. The ratio of RBD “up” and “down” in these variants was analyzed semi-quantitatively through cryo-EM. Through biochemical and structural analysis by mass spectrometry and cryo-EM, we demonstrated that the S31 deletion enabled N-glycosylation at N30 and caused a 180° flip of the side chain of the adjacent F32 residue. The S31 deletion and N30 glycosylation exhibited a minimal effect on the spike protein conformation and did not affect spike binding to hACE2 or to the broadly neutralizing antibody BD55-1205.^19^ However, addition of N-glycans at N30 knocked out the binding of the NTD antibody C1717 and altered the glycoforms at a spatially adjacent N-glycosylation site at residue N61. Through bioinformatics analysis, we also observed an important trend in recent SARS-CoV-2 evolution: many mutations in recent variants of concern recapitulate residues found in other sarbecoviruses, including but not limited to Q493E and the PNGS at N30. Most of these re-occurring mutations have emerged since late 2023. Overall, these findings advance our understanding of current SARS-CoV-2 evolution and provide new insights for prediction of subsequent evolution as well as the design of vaccines and therapeutic antibodies against current and future SARS-CoV-2 variants.

## RESULTS

### Deletion of conserved S31 on the NTD introduces N30 glycosylation

**Figure 1.**
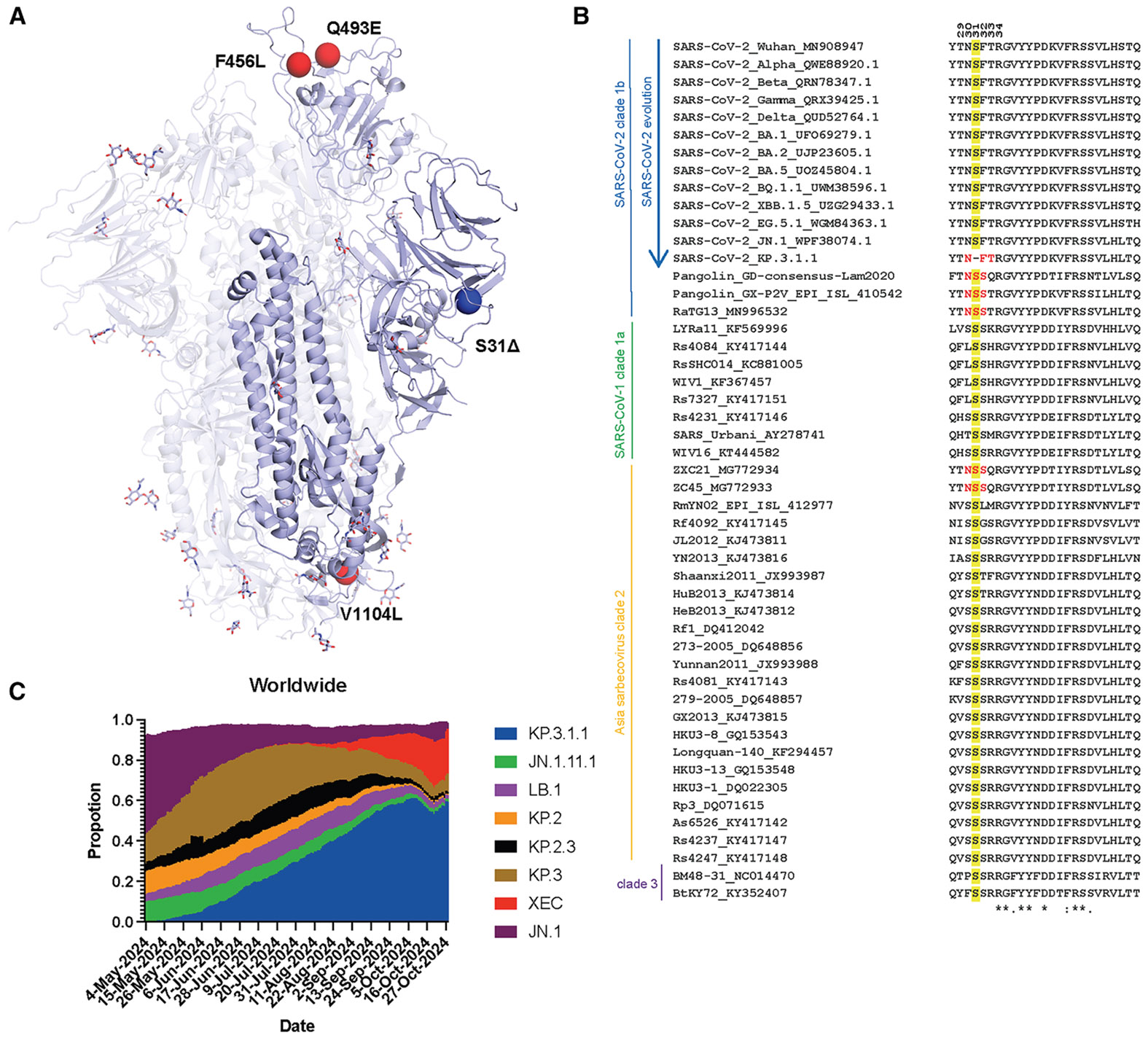
Deletion of conserved S31 on the NTD introduces an N30 glycosylation site (A) Mutations (red) and a deletion (blue) in KP.3.1.1 in the context of JN.1 S (lavender) (PDB: 8Y5J).^1^ (B) Sequence alignment of sarbecoviruses, including several SARS-CoV-2 variants of concern (VOCs). Yellow highlights the conserved S31 across sarbecoviruses except for KP.3.1.1. Red shows the N-glycosylation sequon at N30. Residues identical in all aligned sequences are labeled by an asterisk, whereas a colon and a period indicate strongly similar and less similar sequences, respectively. The sequence alignment was performed with Clustal Omega.^23^ (C) Frequencies of circulating variant JN.1, JN.1.11.1, KP.2, KP.2.3, KP.3, KP.3.1.1, and LB.1 sequences worldwide from May 4, 2024 to November 1, 2024. Data were collected from covSPECTRUM.^12^

Compared to the JN.1 spike protein, KP.3 carries two mutations in the RBD (F456L and Q493E) and one mutation in the S2 domain (V1104L). KP.3.1.1 contains only one additional change compared to KP.3, a deletion in the NTD (S31Δ) (Figure 1A). JN.1.11 is JN.1 plus V1104L, and JN.1.11.1 is JN.1.11 plus F456L. Sequence alignment of sarbecoviruses showed that S31 is conserved in all sarbecoviruses (Figure 1B). The S31Δ changes the NTD sequence from ^30^NSFT^33^ to NFT and introduces a potential N-glycosylation site at N30, which has been previously observed by cryo-EM in certain bat and pangolin sarbecovirus strains.^20-22^ However, in KP.3.1.1, it is a rare instance of an “NxT” sequon at N30 compared to a few other sarbecoviruses with an NxS sequon (Figure 1B). According to the data from Global Initiative on Sharing All Influenza Data (GISAID), KP.3.1.1 has outcompeted other JN.1-derived variants (e.g., JN.1.11.1, KP.2, KP.2.3, KP.3, and LB.1) and became the dominant strain globally as of November 2024^12,13^ (Figure 1C). Thus, the deletion at S31 and the ensuing glycosylation site at N30 appear to endow a fitness advantage over its most recent predecessors.

**Figure 2.**
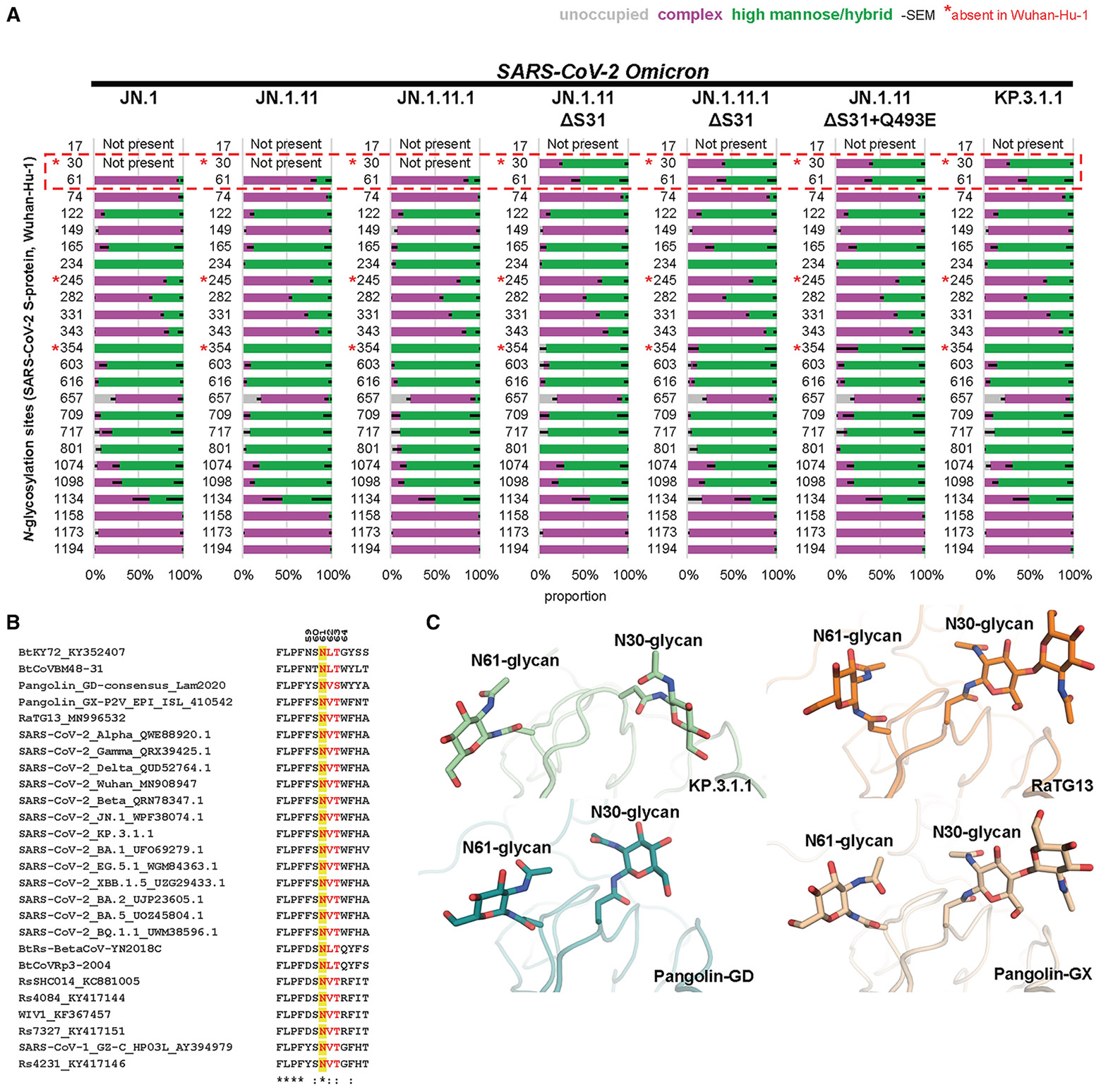
Analysis of the N-glycan landscape on the spike proteins (A) Bar graphs demonstrating site-specific differences in glycosylation, if any, between JN.1 and six natural or engineered mutants (JN.1.11, JN.1.11.1, JN.1.11 + S31Δ, JN.1.11.1 + S31Δ, JN.1.11 + S31Δ+Q493E, and KP.3.1.1) as determined by DeGlyPHER. N-glycosylation states (unoccupied, complex, or high mannose) are color coded. Error bars represent mean – SEM. S31Δ resulted in glycosylation at N30 and a concomitant significant change of complex to mainly high-mannose *N*-glycans at N61 (*p* = 0.002; KP.3.1.1 vs. JN.1). (B) Sequence alignment of sarbecoviruses, including several VOCs of SARS-CoV-2. Yellow highlights the conservation of N61 across all sarbecoviruses, and red shows the N-glycosylation sequon leading to glycosylation at N61. Residues identical in all aligned sequences are labeled by an asterisk, whereas a colon and a period indicate strongly similar and less similar sequences, respectively. The sequence alignment was performed with Clustal Omega.^23^ (C) Magnified view of the close proximity and interaction between the N30 and N61 glycans in KP.3.1.1, RaTG13 (PDB: 6ZGF),^21^ Pangolin-CoV-GD (PDB: 7BBH),^20^ and Pangolin-CoV-GX (PDB: 7CN8).^22^

To validate the potential N-glycosylation site at N30, we utilized the deglycosylation-dependent glycan/proteomic heterogeneity evaluation report (DeGlyPHER)^24^ approach, which combines sequential deglycosylation with bottom-up mass spectrometry-based proteomics to identify N-glycosylation sites and characterize site-specific glycan heterogeneity across seven spike protein variants, including JN.1, JN.1.11, JN.1.11.1, and KP.3.1.1, as well as engineered variants JN.1.11 + S31Δ, JN.1.11.1 + S31Δ, and JN.1.11 + Q493E + S31Δ (Figure 2A). All of the spike proteins in our experiments contain a trimerization domain, HexaPro mutations, and a mutated S1/S2 furin cleavage site. Our results demonstrated that S31Δ indeed induces a fully glycan-occupied N30 glycosylation site in all of the variants carrying the S31 deletion, including JN.1.11 + S31Δ, JN.1.11.1 + S31Δ, JN.1.11 + Q493E + S31Δ, and KP.3.1.1. The N30 site (NxT motif) was found to be predominantly occupied with high-mannose glycans. These findings confirm the previous hypothesis that the S31 deletion would introduce a PNGS at N30.^9,10,15^

### Structural overview of the Omicron KP.3.1.1 and other JN.1-derived mutant spike proteins

**Figure 3.**
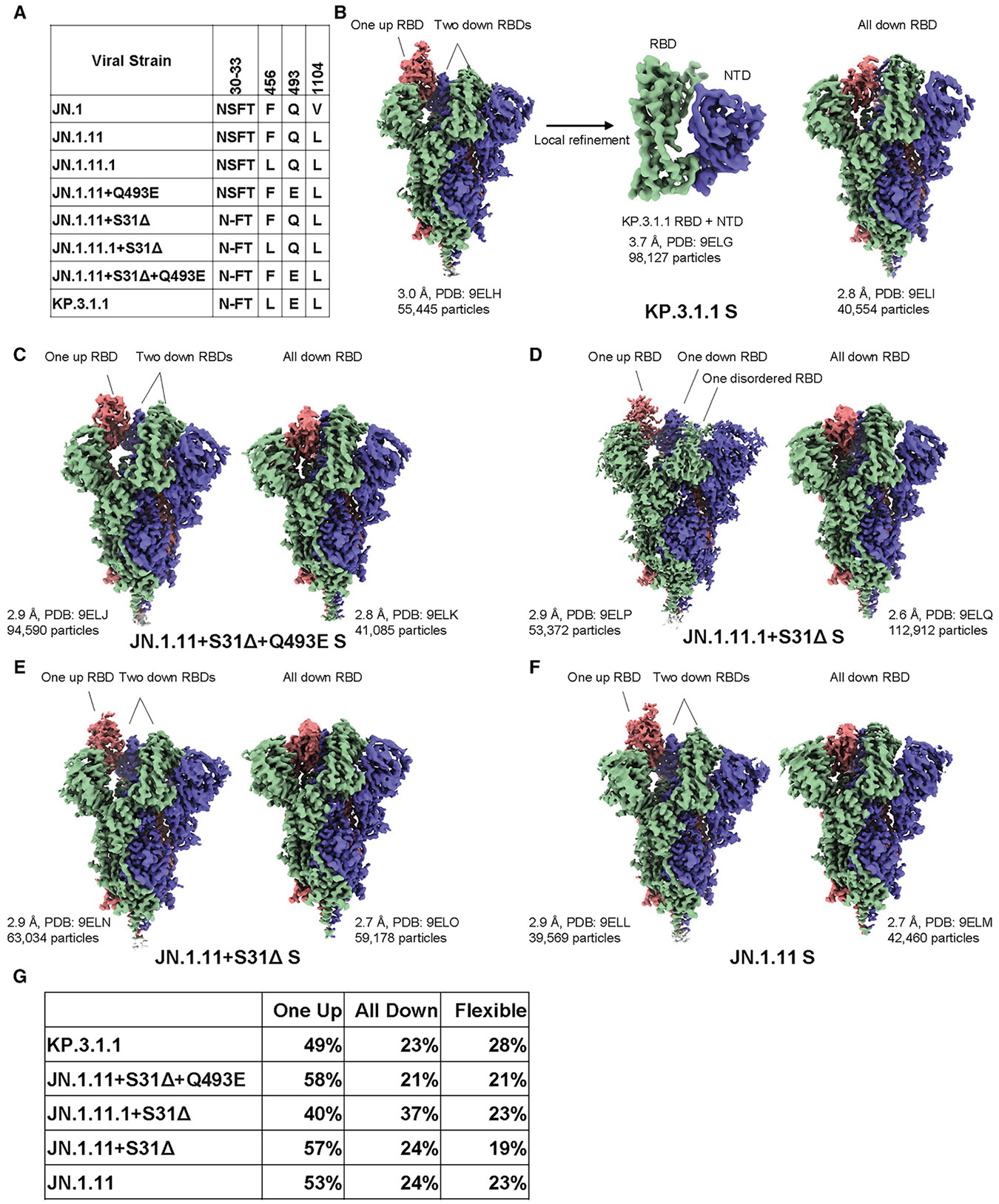
Structural overview of Omicron KP.3.1.1 and other JN.1-derived mutant spike proteins (A) Sequence alignment showing specific mutated amino acids in KP.3.1.1 and other naturally occurring or engineered JN.1-derived spike mutants. (B) Cryo-EM density maps of KP.3.1.1 S (“one RBD up” on the left and “all RBD down” on the right) and map around KP.3.1.1 RBD+NTD (center) after local refinement. Maps are colored by chain, with the three protomers of the spike trimer shown in blue, green, and salmon. (C–F) Cryo-EM density maps of JN.1.11 + S31Δ+Q493E, JN.1.11.1 + S31Δ, JN.1.11 + S31Δ, and JN.1.11 S trimers, respectively, in a side view perpendicular to the 3-fold axis of the trimer. Maps are colored by chain as in (B). Resolution, PDB ID, and particle number are labeled. Left, S in the “one RBD up” conformation. Right, S in the “all RBD down” conformation. (G) The percentage of three conformational states (one RBD up, all RBD down, and flexible (i.e., one or two RBDs disordered in the density maps due to flexibility), observed for the spike proteins in (B)–(F) After 3D variability analysis, particles are clustered into different groups by their conformational state. We then counted the state of each cluster and the number of corresponding particles. See also Figures S1-S5 and Table S1.

To investigate the effects of N30 glycosylation introduced by S31Δ and two RBD mutations (F456L and Q493E) on the structure and function of the spike protein, we determined the cryo-EM structures of the recent SARS-CoV-2 variants KP.3.1.1 and JN.1.11 along with engineered variants JN.1.11 + S31Δ+Q493E, JN.1.11.1 + S31Δ, and JN.1.11 + S31Δ (Figure 3; Table S1; Figures S1 and S2). Besides spike protein, we did not observe co-purified molecules in any of the maps. The spike proteins exhibited three conformational states: “one RBD up,” “all RBD down,” and “flexible” (i.e., one or two RBDs disordered in the density maps due to flexibility). Similar conformational states (“one up” and “all down”) were observed in the spike protein structures of KP.3.1.1’s ancestors, including BA.2, BA.2.12.1, BA.2.75, and BA.2.86, which showed both “one RBD up” and “all RBD down” conformations.^25-27^ Prior studies also indicated that the JN.1 spike protein exhibited either a “one RBD up” or “all RBD down” conformation.^1,28^ Using 3D variability analysis, we quantified the particle proportion for each conformational state and found that S31Δ did not affect the proportions of “one RBD up” and “all RBD down” particles by comparing the proportion of JN.1.11 and other spike proteins that have S31Δ (Figures 3G and S1). Additionally, S31Δ did not markedly affect the thermostability of the spike protein compared to JN1.11, JN1.11.1, and engineered variants, as assessed by nano differential scanning fluorimetry, but retained a substantially increased melting temperature (*T*_m_) over the SARS-CoV-2 wild type (WT) (Wuhan-Hu-1; GenBank: QHD43416.1) (*T*_m_ of 46° C vs. 32° C) (Figure S3). Structural alignment of SARS-CoV-2 WT, JN.1, and JN.1.11 spike proteins with KP.3.1.1 showed that the NTD and RBD exhibited greater rootmean-square deviations (RMSDs) than S2 (Figure S4). V1104L has little effect on any conformational changes; V1104 and L1104 are both surrounded by a conserved hydrophobic pocket formed by P1090, F1095, I1115, and T1120 in the same protomer (Figure S5).

### Molecular basis of KP.3.1.1 S binding to hACE2

**Figure 4.**
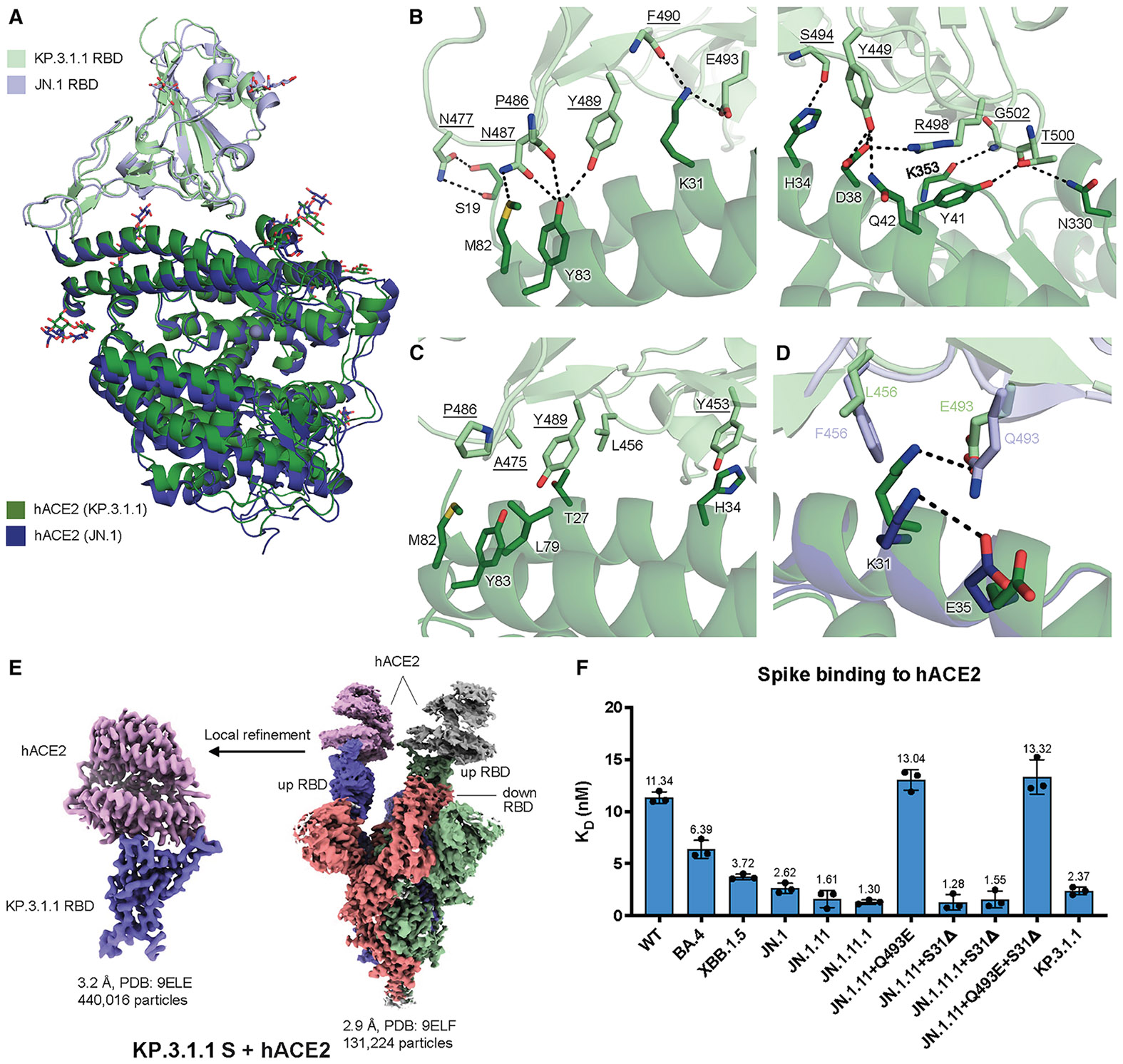
Molecular interactions of the KP.3.1.1 RBD with hACE2 (A) Structural alignment of KP.3.1.1 RBD/hACE2 with JN.1 RBD/hACE2 (PDB: 8Y18)^1^ from cryo-EM structures of their spike proteins with hACE2 using the RBD for alignment. The Cα RMSD between the two RBDs is 0.9 Å. The KP.3.1.1 RBD and hACE2 are colored light green and green, respectively. The JN.1 RBD and its hACE2 are colored light blue and blue, respectively. (B) Molecular details of hydrophilic interactions between the KP.3.1.1 RBD and hACE2. Conserved residues between KP.3.1.1 and JN.1 are underlined. Residues are numbered according to their positions on the SARS-CoV-2 spike protein sequence. Hydrogen bonds and salt bridges are represented by black dashed lines. (C) Hydrophobic interactions between the KP.3.1.1 RBD and hACE2. Aromatic and aliphatic residues are displayed. (D) Structural analysis of cooperative interactions between F456L, Q493E, and hACE2. F456 and Q493 in the JN.1 RBD and L456 and E493 in KP.3.1.1 are displayed. Two salt bridges between K31 and E35 in the JN.1 RBD/hACE2 complex (intrachain) and between K31 and E493 (interchain) in the KP.3.1.1 RBD/hACE2 complex are shown as black dashed lines. (E) Left: cryo-EM density map corresponding to the KP.3.1.1 RBD/hACE2 interaction from focused local refinement of the cryo-EM structure with spike protein and hACE2. Right: cryo-EM density of the entire KP.3.1.1 S/hACE2 complex. Maps are colored by chain, with the three protomers of the spike trimer in blue, green, and salmon and hACE2 in pink and gray. (F) Binding affinity of hACE2 to recombinant spike proteins. Each dot indicates a replicate. Binding affinity is expressed as the nanomolar dissociation constant (K_D_) and displayed above the bars. Data are shown as geometric mean values accompanied by standard deviation (SD). See also Figure S6.

To examine whether S31Δ and the other recent mutations affect S-hACE2 binding, we used biolayer interferometry (BLI) to assess the binding affinities of various spike proteins to hACE2 (Figures 4F and S6). The data from three paired variant sets (JN.1.11 & JN.1.11 + S31Δ, JN.1.11.1 & JN.1.11.1 + S31Δ, and JN.1.11 + Q493E & JN.1.11 + Q493E + S31Δ) showed that S31Δ has little impact on hACE2 binding, which is consistent with previous studies.^10,29^ This result is also concordant with our observation that S31Δ and the resulting N30 glycosylation do not interfere with the up/down configuration of the spike RBD, where the up conformation of the RBD is required for binding to hACE2. Besides, while F456L alone did not affect hACE2 binding (JN.1.11.1 has F456L compared to JN.1.11), Q493E alone substantially reduced binding affinity (Figure 4F), which is also consistent with prior research and is likely due to the electrostatic repulsion between hACE2-E35 and RBD-E493^17^ (Figure 4D).

To investigate the interactions between KP.3.1.1 S and the cell receptor, we also determined a structure of KP.3.1.1 S/hACE2 complex at 2.9 Å resolution using cryo-EM. Two RBDs were found to be in the up position, with each RBD binding to an hACE2 molecule, while the third RBD is in the down position (Figure 4E). The local refinement map of the KP.3.1.1 RBD with hACE2 was also excellent and resolved at 3.1 Å resolution (Figures 4E and S2B; Table S1). Structural alignment with the JN.1 RBD-hACE2 complex (PDB: 8Y18) revealed similar binding (Cα RMSD = 0.9 Å)^1^ (Figure 4A). Residues on the receptor-binding site of the KP.3.1.1 RBD are highly conserved with JN.1 and form extensive electrostatic interactions with hACE2. E493 and R498 form salt bridges with K31 and D38, respectively; the main chains of P486, F490, S494, and G502 form H-bonds with the side chains of Y83, K31, H34, and K353 main chain, respectively; N477 and Y489 H-bond with S19 and Y83, respectively; and Y449 H-bonds with D38 and Q42, N487 with M82 and Y83, and T500 with Y41 and N330 (Figure 4B). Several conserved aromatic and aliphatic residues (Y453, A475, P486, and Y489) also establish hydrophobic interactions with hACE2 (Figure 4C). Q493E alone reduced hACE2 binding, while F456L by itself had minimal impact on the binding; however, hACE2 binding with the Q493E mutation was recovered in the context of F456L (Figures 4F and S6, JN.1.11 + S31Δ+Q493E vs. KP.3.1.1). This phenomenon has been observed and structurally depicted in the KP.3 variant.^17^ Thus, we also observed the epistatic effect of F456L and Q493E in KP.3.1.1, which together enhanced hACE2 binding, compared to the substantially reduced binding with Q493E alone.^16,17^ RBD-Q493E alone in the presence of F456 would likely be disfavored due to an electrostatic repulsion with hACE2-E35 (Figures 4D, 4F, and S6). The bulkier RBD-F456 side chain shifts hACE2-K31 to form an intrachain salt bridge interaction with hACE2-E35, thereby reducing its interaction with RBD E493. However, when the smaller side chain of L456 is present, K31 can move and form a salt bridge with E493 (Figure 4D), which is a stronger interaction than the hydrogen bond between K31 and Q493 in the presence of F456, which explains how F456L and Q493E can synergize to recover the binding affinity with hACE2.

### BD55-1205 binding to KP.3.1.1 and other S31Δ mutants

**Figure 5.**
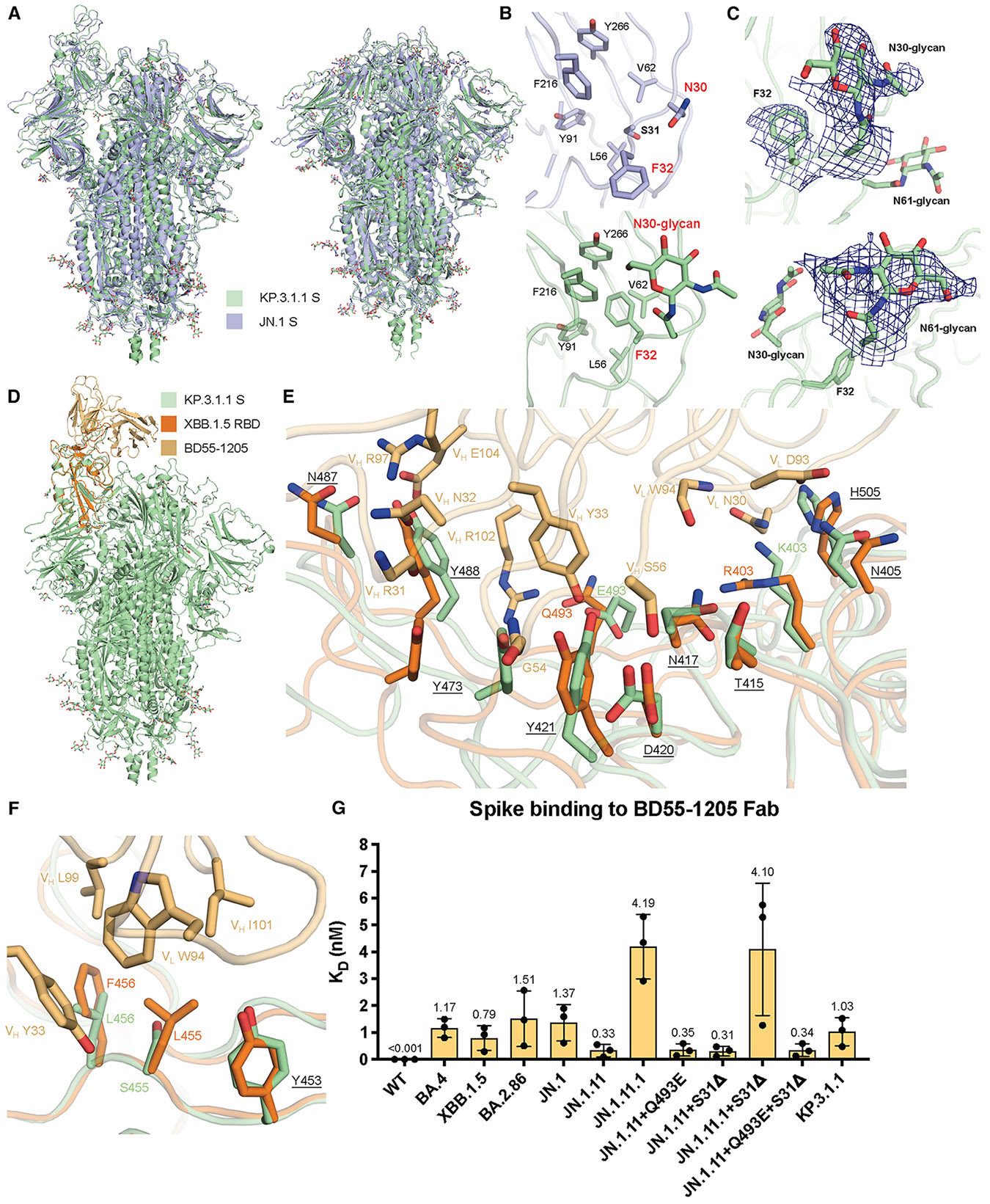
Structural details of S31Δ, N30 glycosylation, and BD55-1205 binding to KP.3.1.1 and other S31Δ mutants (A) Superimposed cryo-EM structures of KP.3.1.1 S and JN.1 S in both “one RBD up” (PDB: 8Y5J)^1^ and “all RBD down” (PDB: 8X4H)^28^ states. Cα RMSDs are 1.1 Å and 0.9 Å, respectively. KP.3.1.1 S and JN.1 S are colored light green and light blue, respectively. (B) Magnified view around the N30 glycan site. Compared to the JN.1 S, S31Δ results in an F32 side chain flip of 180° to now fit into the hydrophobic pocket formed by L56, V62, Y91, F216, and Y266. N30 is glycosylated in KP.3.1.1 S. (C) Magnified view of N30 NAG, F32 side chain, and N61 NAG, with the cryo-EM density map in deep blue. (D) Superimposed structures of KP.3.1.1 S with the XBB.1.5 RBD/BD55-1205 complex (PDB: 8XE9)^19^ to identify possible interactions of BD55-1205 with KP.3.1.1. KP.3.1.1 S, XBB.1.5 RBD, and BD55-1205 are colored in light green, orange, and light orange, respectively. (E and F) Magnified view of the hydrophilic and hydrophobic interactions between XBB.1.5 RBD and BD55-1205, respectively, Corresponding residues in KP.3.1.1 in the binding site are also displayed. Conserved residues between KP.3.1.1 and XBB.1.5 are underlined. (G) Binding affinity of the BD55-1205 Fab to recombinant spike proteins. Each dot indicates a replicate. Binding affinity is expressed as the nanomolar K_D_ and displayed above the bars. Data are shown as geometric mean values accompanied by SD. See also Figures S7-S10.

BD55-1205 is a “class 1” antibody encoded by the IGHV3-53/3–66 germline, showing potent and broad neutralizing ability across all variants from SARS-CoV-2 WT to JN.1.^19^ The epitope residues of BD55-1205 mostly overlap with hACE2, explaining its potent neutralizing ability.^19,30^ To determine whether KP.3.1.1 or other S31Δ variants escape BD55-1205, we used BLI to measure binding affinities between spike proteins and the antibody binding fragment (Fab) (Figures 5G and S7). BD55-1205 retained strong binding to KP.3.1.1 S and other mutants, indicating that S31Δ had minimal effect on BD55-1205 binding, further supporting our previous finding that S31Δ did not alter the RBD up/down ratio. In addition, other spike mutants with Q493E retain BD55-1205 binding, while F456L resulted in a binding affinity decrease where the only mutation of JN.1.11.1 is F456L compared to JN.1.11 (Figures 5G and S7). We superimposed the structure of the BD55-1205/XBB.1.5 RBD complex (PDB: 8XE9) to KP.3.1.1 S, and the RBDs showed a highly similar conformation (Cα RMSD = 0.9 Å) (Figure 5D).^19^ The affinity difference between BD55-1205 and different JN.1 subvariants is similar to previous results^19^ and consistent with Q493E possibly forming a salt bridge with V_H_ R102 (Figure 5E), while F456L may reduce the hydrophobic interactions with V_H_ Y33 and V_L_W94 (Figure 5F). BD55-1205 forms extensive hydrophilic (H-bonds and salt bridges) interactions with the RBD. Furthermore, the epitope residues involved in hydrophilic interactions are highly conserved between XBB.1.5 and KP.3.1.1, explaining the nanomolar binding affinity between BD55-1205 and the KP.3.1.1 spike protein (Figure 5E). However, hydrophobic interactions may be weakened by two mutations, L455S and F456L, (Figure 5F). The binding of BD55-1205 is substantially reduced to all of these variants compared to SARS-CoV-2 WT (Figure 5G).

### Structural implications of S31Δ and N30 glycosylation

As confirmed by mass spectrometry, S31Δ alters the ^30^NSFT^33^ sequence to ^30^N-FT^33^, introducing an N30 glycosylation site on the NTD (Figure 2A). Structural alignment between JN.1 S (S31) and KP.3.1.1 S (S31Δ) in both “one RBD up” and “all RBD down” states revealed similar overall conformations and no change in intra-domain distances (Cα RMSD = 1.1 Å and 0.9 Å, respectively) (Figures 5A and S4), indicating that S31Δ and N30 glycosylation did not induce any substantial conformational changes. To observe a clearer structure of the KP.3.1.1 NTD, we performed local refinement of the NTD and its adjacent RBD (Figure 2B). The map was of good quality and resolved at 3.7 Å resolution (Table S1; Figure S2E). Local refinement of KP.3.1.1 NTD showed that S31Δ results in a 180° flip of the F32 side-chain rotamer into a hydrophobic pocket formed by L56, V62, Y91, F216, and Y266 (Figure 5B). Furthermore, N30 N-acetylglucosamine (NAG), F32 side chain, and N61 NAG densities were clear and interpretable (Figure 5C). JN.1.11 showed an NTD structure similar to JN.1 (Figures S8A and S8B). Similarly, JN.1.11 + S31Δ, JN.1.11.1 + S31Δ, and JN.1.11 + Q493E + S31Δ also shared similar NTD structures with KP.3.1.1 (Figures S8C-S8F). Three sarbecoviruses with N30 glycosylation (RaTG13, Pangolin-GD, and Pangolin-GX) also showed similar NTD structures and N30 glycosylation as KP.3.1.1^20-22^ (Figures S8F-S8I).

In addition, N30 is located in NTD “site vi,” which comprises the binding sites for known antibodies, including the neutralizing antibody C1717 and non-neutralizing antibodies DH1052, COV57, and S2M24.^31,32-34^ Through a structural comparison and binding assay, we showed that N30 glycosylation is positioned so that it knocks out binding to mAb C1717 (Figure S9). When comparing the surface electrostatic potential between KP.3.1.1 NTD and JN.1.11 NTD, we found that the N30 glycan causes a minor effect on adjacent residues (Figure S10).

### Analysis of the N-glycan landscape on the spike proteins

The coronavirus spike proteins are heavily glycosylated, and the SARS-CoV-2 WT spike protein has 22 N-glycosylation sites per protomer.^35,36^ Glycan profiling studies by mass spectrometry have been performed for many early variants,^37-39^ but here we also investigated the glycoforms present at three new glycosylation sites (N30, N245, and N354). Compared to the WT spike protein, all of the seven spike proteins that we analyzed here exhibit two new glycosylation sites (N245 in NTD and N354 in RBD) because of the H245N and K356T mutations that emerged in recent variants, while the N17 glycosylation in early variants is absent due to the T19I mutation that emerged in BA.2 and later variants.^28,40^ N245 is mostly occupied with complex glycans, while N354 is predominantly occupied with high-mannose glycans. Notably, introduction of the S31Δ mutation in KP3.1.1 and engineered JN1.11/JN.1.11.1 spike variants created a glycosylation site at N30 that is populated more than 60% with high-mannose glycans, reducing the percentage of complex glycans from ~80%–95% to <50% at the N61 glycosylation site (Figure 2A). Sequence alignment showed that the N61 glycosylation site is highly conserved across all sarbecoviruses shown here (Figure 2B), suggesting a potentially critical, but not yet determined, role for the N61 glycan. A previous study has shown, however, that N61Q significantly reduces infectivity.^41^ Magnified views of KP.3.1.1, RaTG13, Pangolin-CoV-GD, and Pangolin-CoV-GX indicated that N30 and its attached glycan are spatially proximal to N61 (Figure 2C).^20-22^ We hypothesize that the shift of glycoforms at N61 is a consequence of crowding by the neighboring glycan at N30 and more restricted access to the glycan-processing enzymes. Furthermore, α6-fucosylation of glycans at N61 would likely clash with N30 sugar moieties. Any potential impact of the altered N61 glycoform on its function remains to be investigated. Other N-glycans throughout the spike protein remain largely unchanged.

## DISCUSSION

In this study, we used cryo-EM to determine high-resolution structures of the globally predominant variant KP.3.1.1 in both apo and hACE2-bound states. Our structural analyses of the KP.3.1.1 RBD/hACE2 complex and binding data are consistent with an epistatic interaction between the F456L and Q493E mutations in the RBD of KP.3.1.1, where Q493E alone substantially reduces binding but, in the presence of F456L, these residues synergize to restore hACE2 binding affinity. In the cryo-EM structure of the spike protein of KP.3.1.1, we observed N30 glycosylation and a 180° flip of the F32 side chain, resulting from the S31 deletion in the ^30^NSFT^33^ motif. Additionally, we also determined apo spike protein structures of engineered JN.1-derived mutants carrying S31Δ, F456L, and Q493E. CryoSPARC 3D variability analysis demonstrated that all spike proteins had similar proportions of up and down RBDs, suggesting that S31Δ does not influence the conformational dynamics of the spike protein. Furthermore, S31Δ had minimal effect on hACE2 and BD55-1205 binding affinities and the overall spike thermostability but completely knocked out C1717 binding. Glycan mass spectrometry demonstrated that glycans at the new N30 site are predominantly high mannose and alter the proportions of complex and high-mannose/hybrid glycoforms at N61.

Previous functional studies indicated that, compared to KP.3, KP.3.1.1 exhibits greater resistance to neutralizing antibodies from convalescent plasma, immunized rabbit sera, and vaccinated human sera as well as higher infectivity and reduced binding affinity to monoclonal antibodies across multiple classes.^8,10,14,15,29^ Additionally, the recently emerged XEC variant, which has another N-glycosylation in the NTD due to a T22N mutation but does not have the S31 deletion or N30 glycan (Figure S11), appears to display a similar extent of immune evasion as KP.3.1.1.^10,14,29^ Although we do not have sufficient evidence to link the addition of NTD glycans to immune evasion based only on the current structural analysis of recombinant spike proteins, an increase in density of the glycan shield in the spike due to the additional S31Δ-introduced N30 glycan and blocking of specific antibodies that target epitopes proximal to the added glycan likely contribute to immune escape of KP.3.1.1 and XEC (for the N22 glycan).^9,10^ Further functional studies in live viruses are needed to elucidate the underlying mechanism.

KP.3 contains the F456L and Q493E mutations, which exhibit synergistic effects, significantly enhancing hACE2 binding affinity compared to Q493E mutation alone.^16,17^ In this study, we demonstrated that KP.3.1.1 retains this synergistic epistasis. Epistasis is a common phenomenon in RNA viruses, including vesicular stomatitis virus,^42^ HIV-1,^43^ and influenza virus.^44,45^ It has been shown to play a vital role in enhancing viral fitness and enabling immune escape in SARS-CoV-2.^46,47^ Investigating the effects of mutations in different SARS-CoV-2 variants and other sarbecoviruses may help reveal epistatic interactions that may confer fitness advantages and shape the evolutionary trajectories of SARS-CoV-2.

**Figure 6.**
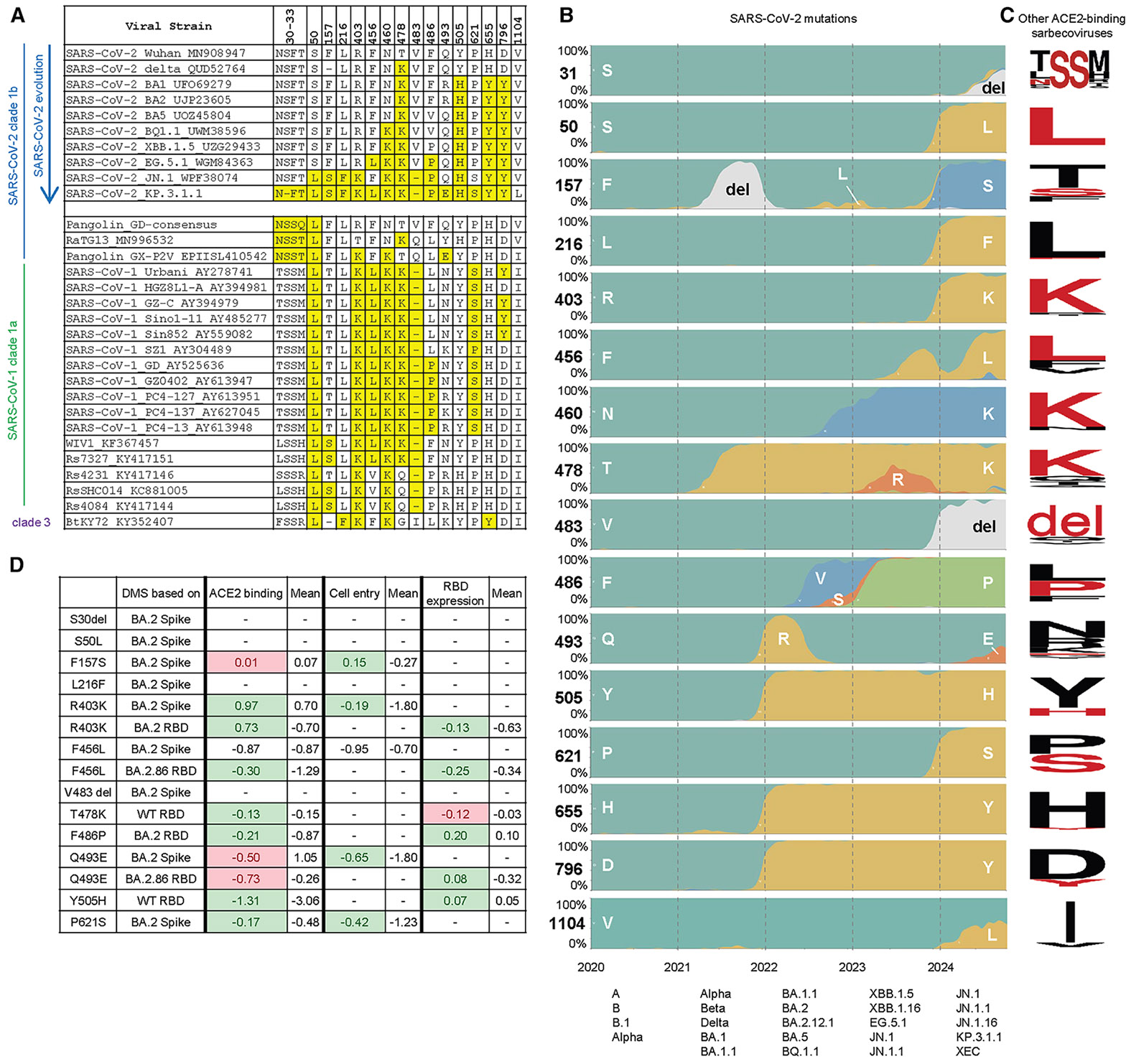
Re-occurrence of amino acids observed in other sarbecoviruses during SARS-CoV-2 evolution (A) Sequence alignment showing recently mutated amino acids in SARS-CoV-2 that are found in other ACE2-binding sarbecoviruses.^49^ Re-occurring amino acids are highlighted in yellow. Sequence accession codes are provided at the end of each virus strain name. (B) Frequency of amino acids at particular positions on SARS-CoV-2 spike as of October 2024. Data were sourced from nextstrain.org (GISAID data).^13,50^ Major variants circulating in each year are listed at the bottom. (C) Sequence conservation analysis of ACE2-binding sarbecoviruses excluding SARS-CoV-2. Each row corresponds to the residue in (B). Amino acids that have re-occurred in the currently dominant SARS-CoV-2 variant KP.3.1.1 are shown in red. The SARS-CoV-2 S31 deletion results in an N-glycosylation site ^30^NFT, corresponding to the N-glycosylation site ^30^NSS in some other sarbecoviruses, which is also highlighted here. The sequence conservation was plotted by WebLogo.^51^ (D) Fold change in hACE2 binding affinity (Δlog10 K_D_), entry into HEK293T cells expressing high ACE2 (functional score), and RBD expression (log10(Mean Fluorescence Intensity)) for assessment of re-occurring mutations. The average was calculated from all mutations at that site. Green highlights where the effect of the re-occurring mutation is greater than the average, and red highlights where the effect of the re-occurring mutation is less than the average. Data are from previous DMS studies.^16,46,48^ See also Figure S11.

The Q493E mutation and additional N-glycosylation site at N30 resulting from S31Δ have only recently emerged in SARS-CoV-2 variants. This glycan has been observed in other clade 1b sarbecoviruses over a prolonged period (Figure 6B), such as pangolin-CoV GD, pangolin-CoV GX-P2V, and RaTG13^20-22^ (Figure 6A). The glycans in all four strains (including SARS-CoV-2 KP.3.1.1 and these three non-SARS-CoV-2 sarbecoviruses) exhibit similar conformations, implying a conserved function of the glycan among sarbecoviruses (Figures 2C and S8). Furthermore, the Q493E mutation in SARS-CoV-2, which emerged in early 2024, is analogous to the E493 residue found in pangolin-CoV GX-P2V. In addition to Q493E and the gained N30 glycosylation site, other SARS-CoV-2 variants also exhibited re-occurrence of amino acids found in the spike proteins of other sarbecoviruses. In total, 15 mutations (S31Δ, S50L, F157S, L216F, R403K, F456L, N460K, T478K, V483del, F486P, Q493E, Y505H, P621S, H655Y, and D796Y) have been found in at least one SARS-CoV-2 VOC or variant of interest (Figure 6B). Notably, nine of these 15 re-occurring mutations (S31Δ, S50L, F157S, L216F, R403K, F456L, V483del, Q493E, and P621S), emerged only recently, suggesting an accelerated re-occurrence rate since late 2023. Data from previous deep mutational scanning (DMS) studies showed that most re-occurring mutations (R403K, F456L, F486P, Y505H, and P621S) performed better than average in hACE2 binding, cell entry, and RBD expression (Figure 6D).^16,46,48^ Although Q493E alone reduced hACE2 binding, it was complemented by F456L, which restored binding affinity. Overall, this analysis of major SARS-CoV-2 variants demonstrates that many of the predominant variants have reversions to amino acids originally found in other sarbecoviruses that may suggest alterations of the mutational landscape trajectory in recent years.

The evolution trajectory of SARS-CoV-2 is largely constrained and driven by immune pressure while maintaining or enhancing viral fitness. The immunity of the global population has been largely imprinted by early SARS-CoV-2 strains, either through vaccination or early infections. While SARS-CoV-2 originally diverged from related sarbecoviruses, many amino acids that were first seen were unique to SARS-CoV-2. Mutations at these residues have facilitated immune escape but sometimes at the cost of viral fitness. Interestingly, amino acids at similar positions in non-SARS-CoV-2 sarbecoviruses, which have demonstrated high viral fitness, are now re-emerging in SARS-CoV-2. These re-occurring mutations likely represent a balance between immune escape and fitness optimization. Notably, we identified 15 mutations during SARS-CoV-2 evolution that correspond to amino acids present in other sarbecoviruses. These findings highlight the evolutionary trajectories that are now possible and productive and are bringing the spike proteins in these sarbecoviruses closer together. These re-occurring mutations now appear to confer some structural and functional advantages as SARS-CoV-2 continues to evolve to maintain fitness and immune escape, offering information on possible evolutionary trajectories for the next set of SARS-CoV-2 variants. Since the selection pressure differs for SARS-CoV-2 and other sarbecoviruses, the same amino acid may play different roles in their respective spike proteins. How these recurring mutations affect viral fitness and immune escape will be experimentally explored in future studies.

Overall, the amino acid re-occurrence in related sarbecoviruses, along with the epistatic mutations observed during SARS-CoV-2 evolution, may play critical roles in enhancing viral fitness and facilitating immune escape. These observations provide invaluable insights for predicting future evolutionary pathways of SARS-CoV-2.

### Limitations of the study

The trimerization domain, HexaPro mutations, and a mutated S1/S2 furin cleavage site were employed to enhance spike protein stability and expression yield. The detergent lauryl maltose neopentyl glycol was used to avoid orientation bias of the spike protein on the cryo-EM grid. Importantly, the results from the *in vitro* recombinant spike protein may not fully recapitulate the behavior of the WT, full-length, membrane-embedded spike protein of the virus.

## STAR★METHODS

### EXPERIMENTAL MODEL AND STUDY PARTICIPANT DETAILS

Cell lines used in this study are listed in key resources table. Cell growth conditions can be found in method details.

### METHOD DETAILS

#### Expression and purification of human ACE2

For cryo-EM and biolayer interferometry (BLI) binding experiments, human ACE2 (residues 19–615, GenBank: BAJ21180.1) was flanked with an N-terminal signal peptide and a C-terminal His_6_ tag and codon optimized in phCMV3 expression vector. Expi293S cells (Life Technologies) were then transfected with ExpiFectamine 293. Reagent and incubated according to manufacturer’s instruction. Supernatants were harvested and proteins were purified by Ni Sepharose excel resin (Cytiva), eluted with 300 mM imidazole, followed by size exclusion (SEC) and buffer exchanged into 20 mM Tris-HCl, 150 mM NaCl, pH 7.4.

#### Expression and purification of BD55-1205 and C1717 Fab

The heavy and light chains of the Fab were cloned into the phCMV3 vector. The plasmids were transiently co-transfected into ExpiCHO cells at a ratio of 2:1 (heavy chain to light chain) using ExpiFectamine CHO Reagent (Thermo Fisher Scientific) according to the manufacturer’s instructions. The supernatant was collected at 7 days post-transfection. The Fab was purified with a CaptureSelect CH1-XL Pre-packed Column (Thermo Fisher Scientific), followed by SEC and buffer exchanged 20 mM Tris-HCl, 150 mM NaCl, pH 7.4.

#### Recombinant full-length SARS-CoV-2 prefusion spike protein and mutants

All spike ectodomain constructs contain a C-terminal T4 fibritin trimerization domain^59^ and a Twin-strep-tag for purification. For the SARS-CoV-2 spike, we synthesized a base construct (HP-GSAS) with residues 1 to 1208 from the WT strain (GenBank: QHD43416.1) with six stabilizing proline (HexaPro or S6P)^60^ substitutions at positions 817, 892, 899, 942, 986, and 987 and the S1/S2 furin cleavage site modified to 682-GSAS-685. The S6P expression construct containing the Omicron BA.4/BA.5 mutations (T19I, L24S, del25-27, del69-70, G142D, V213G, G339D, S371F, S373P, S375F, T376A, D405N, R408S, K417N, N440K, G446S, L452R, S477N, T478K, E484A, F486V, Q498R, N501Y, Y505H, D614G, H655Y, N658S, N679K, P681H, N764K, D796Y, Q954H, N969K) were assembled from synthesized DNA fragments. XBB.1.5, BA.2.86, JN.1, JN.1.11, JN.1.11.1, KP.3.1.1, and other mutants (JN.1.11 + S31Δ, JN.1.11 + Q493E, JN.1.11 + S31Δ+Q493E, JN.1.11.1 + S31Δ, and KP.3.1.1) were also synthesized by this method.

#### Expression and purification of recombinant spike proteins

For protein expression, Expi293F cells (Thermo Fisher Scientific: A14527, RRID: CVCL_D615) and FreeStyle 293-F cells (Thermo Fisher Scientific: R79007, RRID: CVCL_D603) were transfected with the spike plasmid of interest. Transfected cells were incubated at 37° C with shaking at 110 r.p.m. for 6 days for protein expression. The spike proteins were purified from the supernatants with Strep-TactinXT 4Flow^®^ high capacity (IBA Lifescience GmbH: 2-5010-025) and buffer-exchanged to TBS (20 mM Tris-HCl and 150 mM NaCl, pH 7.4) before further purification with SEC. Protein fractions corresponding to the trimeric spike proteins were collected and concentrated.

#### Biolayer interferometry (BLI) binding assays

Binding assays were performed by biolayer interferometry (BLI) using an Octet Red instrument (FortéBio). Each spike protein was tested in triplicate.

For spike binding to hACE2 and BD55-1205 Fab, twin-strep-tagged spike proteins at 20 μg/mL in 1x kinetics buffer (1x PBS, pH 7.4, 0.01% BSA, and 0.002% Tween 20) were loaded onto streptavidin (SA) sensors, and then incubated with 6.25, 12.5, 25, 50, and 100 nM of hACE2 or BD55-1205 Fab. The assay consisted of five steps: 1) baseline: 60 s with 1x kinetics buffer; 2) loading: 180 s with Twin-strep-tagged spike proteins; 3) baseline: 90 s with 1x kinetics buffer; 4) association: 240 s with hACE2 or antibody; and 5) dissociation: 240 s with 1x kinetics buffer. For estimating the K_D_ values, a 1:1 binding model was used.

For C1717 Fab binding to spikes, C1717 Fab 10 μg/mL in 1x kinetics buffer (1x PBS, pH 7.4, 0.01% BSA, and 0.002% Tween 20) were loaded onto FAB2G sensors, and then incubated with 3.7, 11.1, 33.3, 100, and 300 nM of various spikes. The assay consisted of five steps: 1) baseline: 60 s with 1x kinetics buffer; 2) loading: 90 s with C1717 Fab; 3) baseline: 90 s with 1x kinetics buffer; 4) association: 300 s with various spikes; and 5) dissociation: 300 s with 1x kinetics buffer. For estimating the K_D_ values, a 1:1 binding model was used.

#### Cryo-EM sample preparation

For JN.1.11, JN.1.11 + S31Δ, KP.3.1.1, 3 μL of spike protein in TBS (20 mM Tris-HCl and 150 mM NaCl, pH 7.4) at 0.7 mg/mL was mixed with 0.5 μL of 0.035 mM lauryl maltose neopentyl glycol (LMNG) solution immediately before sample deposition onto an UltrAuFoil 1.2/1.3 (Au, 300-mesh; Quantifoil Micro Tools GmbH) grid that had been plasma cleaned for 25 s using a PELCO easiGlow Glow Discharge Cleaning System (Ted Pella Inc.). Following sample application, grids were blotted for 3.5 s before being vitrified in liquid ethane using a Vitrobot Mark IV (Thermo Fisher) operating at 4° C and 100% humidity. For JN.1.11.1 + S31Δ and JN.1.11 + S31Δ+Q493E, blot time was 3 s. The KP.3.1.1 spike protein was incubated with a 3-fold molar excess of hACE2 (protomer: hACE2 = 1:1, final concentration of 0.75 mg/mL) for 30 min and following the same procedure as for the apo samples with 3 s blot time. The grids with glassy ice were stored in liquid nitrogen before data collection.

#### Cryo-EM data collection and processing

Micrographs were collected on a Glacios 2 microscope (Thermo Fisher) operating at 200 kV equipped with a Falcon 4i detector (Thermo Fisher). Nominal magnification and pixel size were 190,000x and 0.718 Å respectively. Data were collected with EPU (ThermoFisher) with a total cumulative dose of 44.84 e^−^/Å^2^ and a nominal defocus range of −0.8 to −1.7 μm. CryoSPARC Live Patch Motion Correction was used for alignment and dose weighting of movies. CTF estimations, particle picking, particle extraction, iterative rounds of 2D classification, ab-initio reconstruction, heterogeneous refinement, homogeneous refinement, 3D Variability, global CTF refinement, and non-uniform refinement were performed on CryoSPARC.^54^

#### Model building and refinement

Initial model building was performed manually in Coot using JN.1 “One RBD Up” S (PDB: 8Y5J), JN.1 “All RBD Down” S (PDB: 8X4H), and JN.1 S/hACE2 complex (PDB: 8Y16) as the templates.^1,28^ Followed by iterative rounds of Rosetta relaxed refinement, Phenix real space refinement, and Coot manual refinement to generate the final models.^55,56,61^ EMRinger and MolProbity were run following each round of Rosetta refinement to evaluate and choose the best refined models.^57,58^ Final map and model statistics are summarized in Table S1. Figures were generated using PyMOL, UCSF Chimera, and UCSF Chimera X.^52,53^

#### Nano differential scanning fluorimetry

NanoDSF was performed using Prometheus PANTA (NanoTemper Technologies, München, Germany). Samples were loaded in nanoDSF grade standard capillaries (NanoTemper Technologies GmbH, München, Germany) and exposed at thermal stress from 20° C to 95° C by thermal ramping rate of 1° C/min. Each spike protein was tested in triplicate. Fluorescence emission from tryptophan after UV excitation at 280 nm was collected at 330 nm and 350 nm with a dual-UV detector. Thermal stability parameter *T*_m_ was calculated by Panta Analysis software (NanoTemper Technologies, München, Germany). Here the first *T*_m_ was used that may correspond to unfolding of the trimer compared to all of the individual domains.

#### Mass spectrometry-based N-glycan analysis

DeGlyPHER^24,62^ was used to ascertain site-specific glycan occupancy and processivity on the examined glycoproteins.

#### Proteinase K and trypsin treatment

Recombinant SARS-CoV-2 S-glycoprotein was exchanged to water using the Microcon Ultracel PL-10 centrifugal filter. Glycoprotein was reduced with 5 mM tris (2-carboxyethyl) phosphine hydrochloride (TCEP-HCl) and alkylated with 10 mM 2-Chloroacetamide in 100 mM ammonium acetate for 20 min at room temperature (RT, 24° C). Glycoprotein was digested with 1:25 Proteinase K (PK) for 30 min at 37° C and subsequently analyzed by LC-MS/MS.

#### LC-MS/MS

Samples were analyzed on an Orbitrap Eclipse Tribrid mass spectrometer. Samples were injected directly onto a 25 cm, 100 μm ID column packed with BEH 1.7 μm C18 resin. Samples were separated at a flow rate of 300 nL/min on an EASY-nLC 1200 UHPLC. Buffers A and B were 0.1% formic acid in 5% and 80% acetonitrile, respectively. The following gradient was used: 1–25% B over 100 min, an increase to 40% B over 20 min, an increase to 90% B over another 10 min and held for 10 min at 90% B for a total run time of 140 min. The column was re-equilibrated with buffer A prior to the injection of the sample. Peptides were eluted from the tip of the column and nanosprayed directly into the mass spectrometer by application of 2.8 kV at the back of the column. The mass spectrometer was operated in a data-dependent mode. Full MS1 scans were collected in the Orbitrap at 120,000 resolution. The cycle time was set to 3s and, within this 3s, the most abundant ions per scan were selected for HCD MS/MS at 35 NCE. Dynamic exclusion was enabled with exclusion duration of 60 s and singly charged ions were excluded.

#### LC-MS/MS data processing

Protein and peptide identification were carried out with the Integrated Proteomics Pipeline (IP2). Tandem mass spectra were extracted from raw files using RawConverter^63^ and searched with ProLuCID^64^ against a database comprising UniProt reviewed (Swiss-Prot) proteome for *Homo sapiens* (UP000005640), UniProt amino acid sequences for Endo H (P04067), PNGase F (Q9XBM8), and Proteinase K (P06873), amino acid sequences for the examined proteins, and a list of general protein contaminants. The search space included no cleavage specificity. Carbamidomethylation (+57.021460 C) was considered a static modification. Deamidation in the presence of H_2_^18^O (+2.988261 N), GlcNAc (+203.079373 N), oxidation (+15.994915 M), and N-terminal pyroglutamate formation (−17.026549 Q) were considered differential modifications. Data were searched with 50 ppm precursor ion tolerance and 50 ppm fragment ion tolerance. We utilized a target-decoy database search strategy to limit the false discovery rate to 1%, at the spectrum level.^65^ A minimum of one peptide per protein, and no tryptic end per peptide for PK and one tryptic end per peptide for trypsin were required and precursor delta mass cut-off was fixed at 10 ppm. Statistical models for peptide mass modification (modstat) were applied. Census2^66^ label-free analysis was performed based on the precursor peak area, with a 10 ppm precursor mass tolerance and 0.1 min retention time tolerance. Data analysis using GlycoMSQuant^24^ was implemented to automate the analysis. GlycoMSQuant summed precursor peak areas across replicates, discarded peptides without NGS, discarded misidentified peptides when N-glycan remnant-mass modifications were localized to non-PNGS asparagines, and corrected/fixed N-glycan mislocalization where appropriate. Resultant PNGS were aligned to the SARS-CoV-2 WT S-protein sequence from UniProt (P0DTC2).

#### Sequence analysis of re-occurring amino acids

Clustal Omega^67^ was used to perform sequence alignments for the analysis of the re-occurrence of amino acids observed in other sarbecoviruses during SARS-CoV-2 evolution. Sequence accession codes for each species and strain are listed in Figure 6A. Only sarbecoviruses shown to bind ACE2 were included in this analysis.^49^ The frequency of amino acids at particular positions on the SARS-CoV-2 spike was obtained from nextstrain.org,^51^ based on GISAID data.^13^

### QUANTIFICATION AND STATISTICAL ANALYSIS

Cryo-EM data collection and refinement statistics are summarized in Table S1. All data were collected as triplicates and the standard deviation was indicated as error bar (stated in the figure legends Figures 4F, 5G, and S3B). Standard deviation/error calculations were performed using GraphPad Prism.

### KEY RESOURCES TABLE

## Supplementary Material

1

Supplemental information can be found online at https://doi.org/10.1016/j.celrep.2025.115941.

## Figures and Tables

**Table:** 

REAGENT or RESOURCE	SOURCE	IDENTIFIER
Chemicals, peptides, and recombinant proteins		
Pierce TCEP HCl	Thermo Scientific	Cat#20490
Ammonium acetate ≥98%	Sigma-Aldrich	Cat#A1542
2-Chloroacetamide ≥98%	Sigma-Aldrich	Cat#22790
Calcium chloride	Sigma-Aldrich	Cat#C5670
Trypsin	Promega	Cat#V5111
Proteinase K	Sigma-Aldrich	Cat#P2308
Acetonitrile	Honeywell	Cat#AH015-4
Formic acid 90%	J.T.Baker	Cat#0129-01
Corning^®^ Dulbecco’s Phosphate-Buffered Saline, 1X without calcium and magnesium	Thermo Fisher Scientific	Cat#21-031-CV
Critical commercial assays		
Octet^®^ SA Biosensors	Sartorius	Cat#18-5019
Octet^®^ FAB2G Biosensors	Sartorius	Cat#18-5126
NEBuilder^®^ HiFi DNA Assembly Master Mix	New England Biolabs	Cat#E2621L
ExpiCHO Expression System Kit	Gibco	Cat#A29133
Expi293 Expression System Kit	Gibco	Cat#A14635
Prometheus NT.Plex nanoDSF Grade High Sensitivity Capillary Chips	NanoTemper Technologies GmbH	Cat#PR-AC006
Deposited data		
Mass spectrometry and proteomics analysis	Pride/MassIVE	MSV000096635
Cryo-EM structure of SARS-CoV-2 Omicron KP.3.1.1 spike protein (closed state)	This paper	PDB: 9ELI, EMDB: EMD-48150
Cryo-EM structure of SARS-CoV-2 Omicron JN.1.11.1 + S31Δ spike protein (closed state)	This paper	PDB: 9ELQ, EMDB: EMD-48159
Cryo-EM structure of SARS-CoV-2 Omicron JN.1.11 + Q493E + S31Δ spike protein (closed state)	This paper	PDB: 9ELK, EMDB: EMD-48152
Cryo-EM structure of SARS-CoV-2 Omicron JN.1.11 + S31Δ spike protein (closed state)	This paper	PDB: 9ELO, EMDB: EMD-48157
Cryo-EM structure of SARS-CoV-2 Omicron JN.1.11 spike protein (closed state)	This paper	PDB: 9ELM, EMDB: EMD-48155
Cryo-EM structure of SARS-CoV-2 Omicron KP.3.1.1 spike protein (one RBD up state)	This paper	PDB: 9ELH, EMDB: EMD-48149
Cryo-EM structure of SARS-CoV-2 Omicron JN.1.11.1 + S31Δ spike protein (one RBD up state)	This paper	PDB: 9ELP, EMDB: EMD-48158
Cryo-EM structure of SARS-CoV-2 Omicron JN.1.11 + Q493E + S31Δ spike protein (one RBD up state)	This paper	PDB: 9ELJ, EMDB: EMD-48151
Cryo-EM structure of SARS-CoV-2 Omicron JN.1.11 + S31Δ spike protein (one RBD up state)	This paper	PDB: 9ELN, EMDB: EMD-48156
Cryo-EM structure of SARS-CoV-2 Omicron JN.1.11 spike protein (one RBD up state)	This paper	PDB: 9ELL, EMDB: EMD-48153
Cryo-EM structure of SARS-CoV-2 Omicron KP.3.1.1 NTD with RBD (local refinement from the spike protein)	This paper	PDB: 9ELG, EMDB: EMD-48148
Cryo-EM structure of SARS-CoV-2 Omicron KP.3.1.1 spike protein in complex with human ACE2	This paper	PDB: 9ELF, EMDB: EMD-48147
Cryo-EM structure of SARS-CoV-2 Omicron KP.3.1.1 RBD in complex with human ACE2 (local refinement from the spike protein)	This paper	PDB: 9ELE, EMDB: EMD-48146
Cryo-EM map of SARS-CoV-2 Omicron KP.3.1.1 spike protein (flexible state)	This paper	EMDB: EMD-49904
Cryo-EM map of SARS-CoV-2 Omicron JN.1.11.1 + S31Δ spike protein (flexible state)	This paper	EMDB: EMD-49908
Cryo-EM map of SARS-CoV-2 Omicron JN.1.11 + Q493E + S31Δ spike protein (flexible state)	This paper	EMDB: EMD-49905
Cryo-EM map of SARS-CoV-2 Omicron JN.1.11 + S31Δ spike protein (flexible state)	This paper	EMDB: EMD-49907
Cryo-EM map of SARS-CoV-2 Omicron JN.1.11 spike protein (flexible state)	This paper	EMDB: EMD-49906
Experimental models: Cell lines		
ExpiCHO-S^™^ Cells	Thermo Fisher Scientific	Cat#A29127; RRID: CVCL_5J31
Expi293F^™^ cells	Thermo Fisher Scientific	Cat#A14527; RRID: CVCL_D615
Expi293F^™^ GnTI- Cells	Thermo Fisher Scientific	Cat#A14528
Recombinant DNA		
phCMV3	Genlantis	Cat# P003300
Software and algorithms		
ProLuCID	IP2, Bruker Scientific LLC	http://fields.scripps.edu/yates/wp/?page_id=821
DTASelect2.0	IP2, Bruker Scientific LLC	http://fields.scripps.edu/yates/wp/?page_id=816
Census2.0	IP2, Bruker Scientific LLC	http://fields.scripps.edu/yates/wp/?page_id=824
GlycoMSQuant v.1.8.2	John R. Yates III, Scripps Research, La Jolla	https://github.com/proteomicsyates/GlycoMSQuant
RAWConverter	John R. Yates III, Scripps Research, La Jolla	http://fields.scripps.edu/rawconv/
XCalibur	Thermo Scientific	Cat#OPTON-30965-7; RRID:SCR_014593
PyMOL	PyMOL by Schrödinger	https://pymol.org: RRID: SCR_000305
UCSF ChimeraX	Goddard et al.^52^	https://www.cgl.ucsf.edu/chimerax/; RRID: SCR_015872
UCSF Chimera	Pettersen et al.^53^	https://www.cgl.ucsf.edu/chimera/; RRID: SCR_004097
WebLogo	Crooks et al.^51^	https://weblogo.berkeley.edu; RRID: SCR_010236
EPU 2 software	Thermo Fisher Scientific	N/A
ForteBio Data Analysis software	Sartorius	https://www.sartorius.com/en
cryoSPARC.v4	Punjani et al.^54^	RRID: SCR_016501
Coot	Emsley et al.^55^	RRID: SCR_014222
Phenix	Adams et al.^56^	RRID: SCR_014224
EMRinger	Barad et al.^57^	N/A
MolProbity	Williams et al.^58^	RRID: SCR_014226
Prism 10.1.0	GraphPad	https://www.graphpad.com/; RRID: SCR_002798
Other		
Orbitrap Eclipse	Thermo Scientific	N/A
EASY-nLC 1200	Thermo Scientific	Thermo ScientificCat#LC140
Laser-based micropipette puller system	Sutter Instrument	Cat#P-2000
Pressure bomb	customized with Swagelok	https://www.swagelok.com/
MJ Mini Personal Thermal Cycler	BioRad	Cat#PTC1148EDU
Microfuge18 centrifuge	Beckman Coulter	Cat#367160
ÄKTA pure chromatography system	GE Healthcare	N/A
TemCam F415 CMOS camera	TVIPS	N/A
Vitrobot mark IV	Thermo Fisher Scientific	N/A
PELCO easiGlow	Ted Pella Inc.	N/A
Glacios	Thermo Fisher Scientific	N/A
Falcon 4 direct electron detector	Thermo Fisher Scientific	N/A
Prometheus PANTA	NanoTemper Technologies GmbH	N/A
Microcon Ultracel PL-10 centrifugal filters	Millipore Sigma	Cat#MRCPRT010
Resin, Acquity UPLC^®^ BEH 1.7 μm C18	Waters	Cat#186004690
Polymicro fused silica capillary tubing 100 μm	Molex	Cat#1068150023
11 PP Vial Crimp/Snap 250 μL	Thermo Scientific	Cat#C4011-13
Snap-it Seal	Thermo Scientific	Cat#C4011-53
0.2 mL Thin-walled Tubes	Thermo Scientific	Cat#AB-0620
Protein LoBind Tube 1.5 mL	Eppendorf	Cat#022431081
DeckWorks low binding pipette tips (200 μL)	Corning	Cat#4151
Pierce HeLa protein digest standard	Thermo Scientific	Cat#88329
0.2 μm membrane filters	Fisher Scientific	Cat#564-0020
HisPur Ni-NTA Resin	Thermo Fisher Scientific	Cat#88221
CaptureSelect CH1-XL Pre-packed Column	Thermo Fisher Scientific	Cat#494346201
Superdex 200 Increase10/300 GL column	GE Healthcare	Cat#GE28-9909-44
Amicon tubes (100K, 30K, 10K)	Millipore	Cat#UFC9100, Cat#UFC9030, Cat#UFC9010
Strep-Tactin^®^XT 4Flow^®^ high-capacity resin	IBA Lifesciences GmbH	Cat#2-5030-010
Quantifoil^®^ R 1.2/1.3 300 Mesh, Au	Quantifoil Micro Tools GmbH	Cat#Q350AR13A

## Highlights

- Cryo-EM structure of KP.3.1.1 spike protein and its complex with hACE2
- S31 deletion in NTD of KP.3.1.1 results in N30 glycosylation and a 180° flip of F32
- S31 deletion and additional N30 glycan do not affect spike conformation or hACE2 binding
- Recent SARS-CoV-2 variant mutations recapitulate amino acids in other sarbecoviruses
