# Supplementary material for: Structural and functional insights into the evolution of SARS-CoV-2 KP.3.1.1 spike protein
Source: Cell Rep. Author manuscript; Available in PMC 2025 Sep 2. (PMC12404242; doi:10.1016/j.celrep.2025.115941)
Supplement: 1 [file NIHMS2099546-supplement-1.pdf]

Cell Reports, Volume *44*

Supplemental information

Structural and functional insights

into the evolution

of SARS-CoV-2 KP.3.1.1 spike protein

Ziqi Feng, Jiachen Huang, Sabyasachi Baboo, Jolene K. Diedrich, Sandhya Bangaru, James C. Paulson, John R. Yates III, Meng Yuan, Ian A. Wilson, and Andrew B. Ward

Schematic representation of the cryo-EM processing workflow in CryoSPARC (KP.3.1.1)

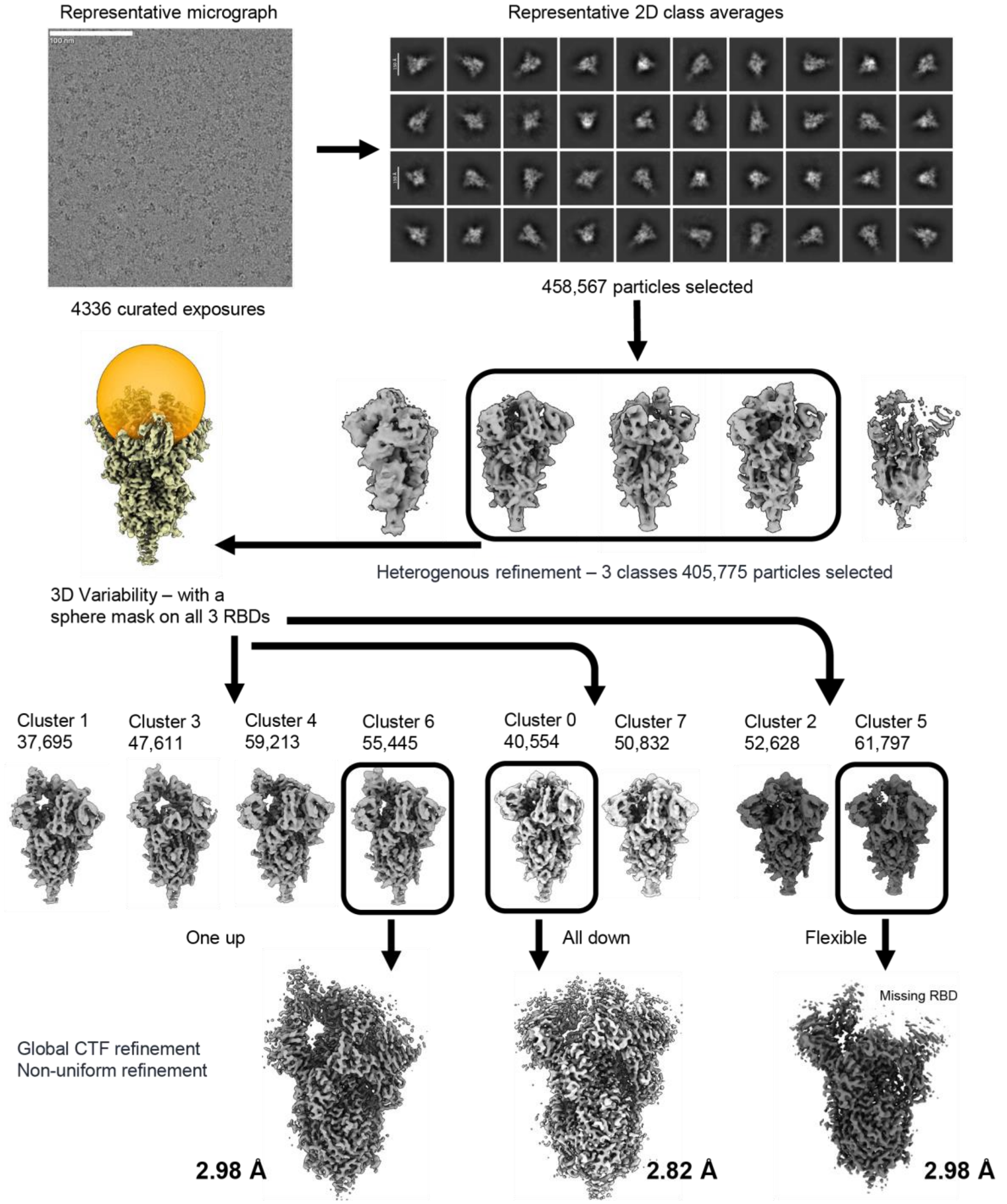

Figure S1. Schematic representation of the cryo-EM processing workflow for SARS-CoV-2 KP.3.1.1 apo spike, related to Figure 3.

Workflow in cryoSPARC including initial processing steps (motion correction, CTF estimation, micrograph selection, particle picking, and selection based on 2D classification and heterogenous refinement) and 3D variability with a sphere mask on all three RBDs. Top left: representative micrograph of KP.3.1.1 apo spike, scale bar: 100 nm. All particles were then separated into eight clusters and classified by their conformation.

A representative map from each class underwent global CTF refinement and non-uniform refinement and was used for model building. This workflow has been applied to all datasets.

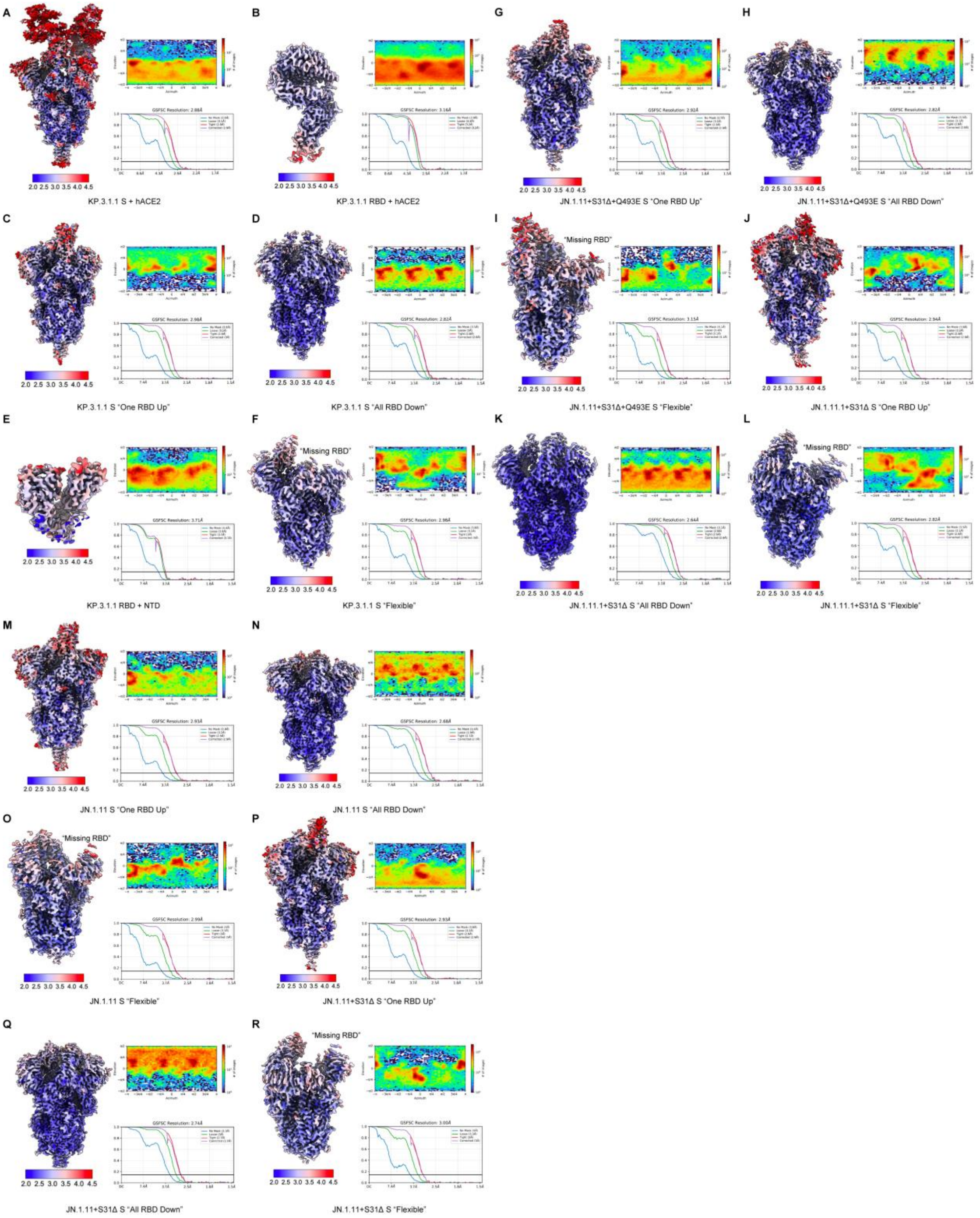

Figure S2. Angular distribution, corresponding FSC curves, and resolution distribution of the cryo-EM map, related to Figure 3.

In the global and local resolution distribution of the cryo-EM maps, blue represents the higher resolution areas (2 Å), while red represents lower resolution (4.5Å). The missing RBDs in “Flexible” S are pointed.

(A) KP.3.1.1 S + hACE2.

(B) KP.3.1.1 RBD + hACE2.

(C) KP.3.1.1 S “One RBD Up”.

(D) KP.3.1.1 S “All RBD Down”.

(E) KP.3.1.1 RBD + NTD.

(F) KP.3.1.1 S “Flexible”

(G) JN.1.11+S31Δ+Q493E S “One RBD Up”.

(H) JN.1.11+S31Δ+Q493E S “All RBD Down”.

(I) JN.1.11+S31Δ+Q493E S “Flexible”.

(J) JN.1.11.1+S31Δ S “One RBD Up”.

(K) JN.1.11.1+S31 Δ S “All RBD Down”.

(L) JN.1.11.1+S31 Δ S “Flexible”.

(M) JN.1.11 S “One RBD Up”.

(N) JN.1.11 S “All RBD Down”.

(O) JN.1.11 S Flexible”.

(P) JN.1.11+S31Δ S “One RBD Up”.

(Q) JN.1.11+S31Δ S “All RBD Down”.

(R) JN.1.11+S31Δ S “Flexible”.

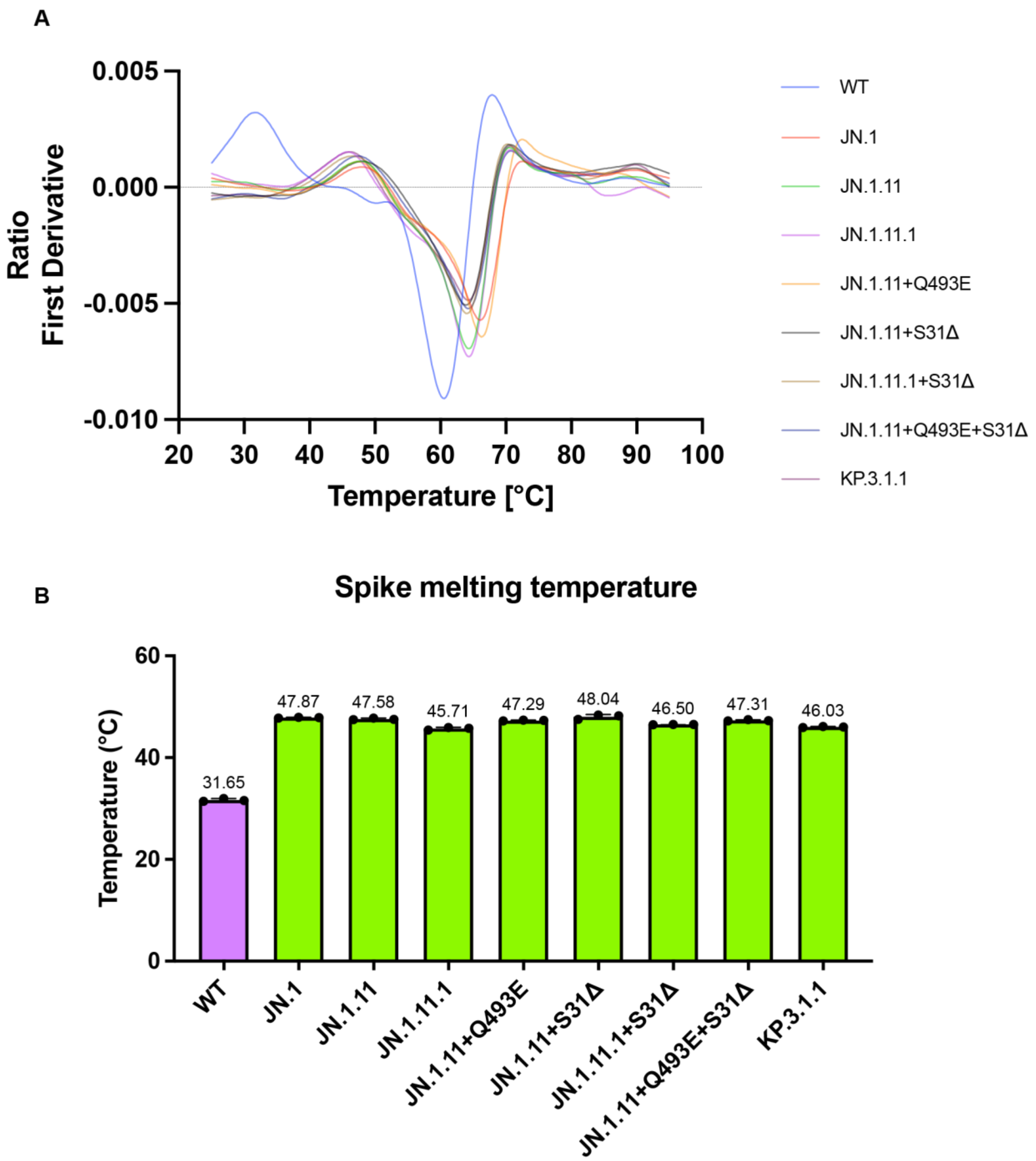

Figure S3. Differential scanning fluorimetry analysis of spike variant thermostability. Melting temperature of spike proteins are displayed above the bars. Data are shown as geometric mean values accompanied by standard deviation, related to Figure 3.

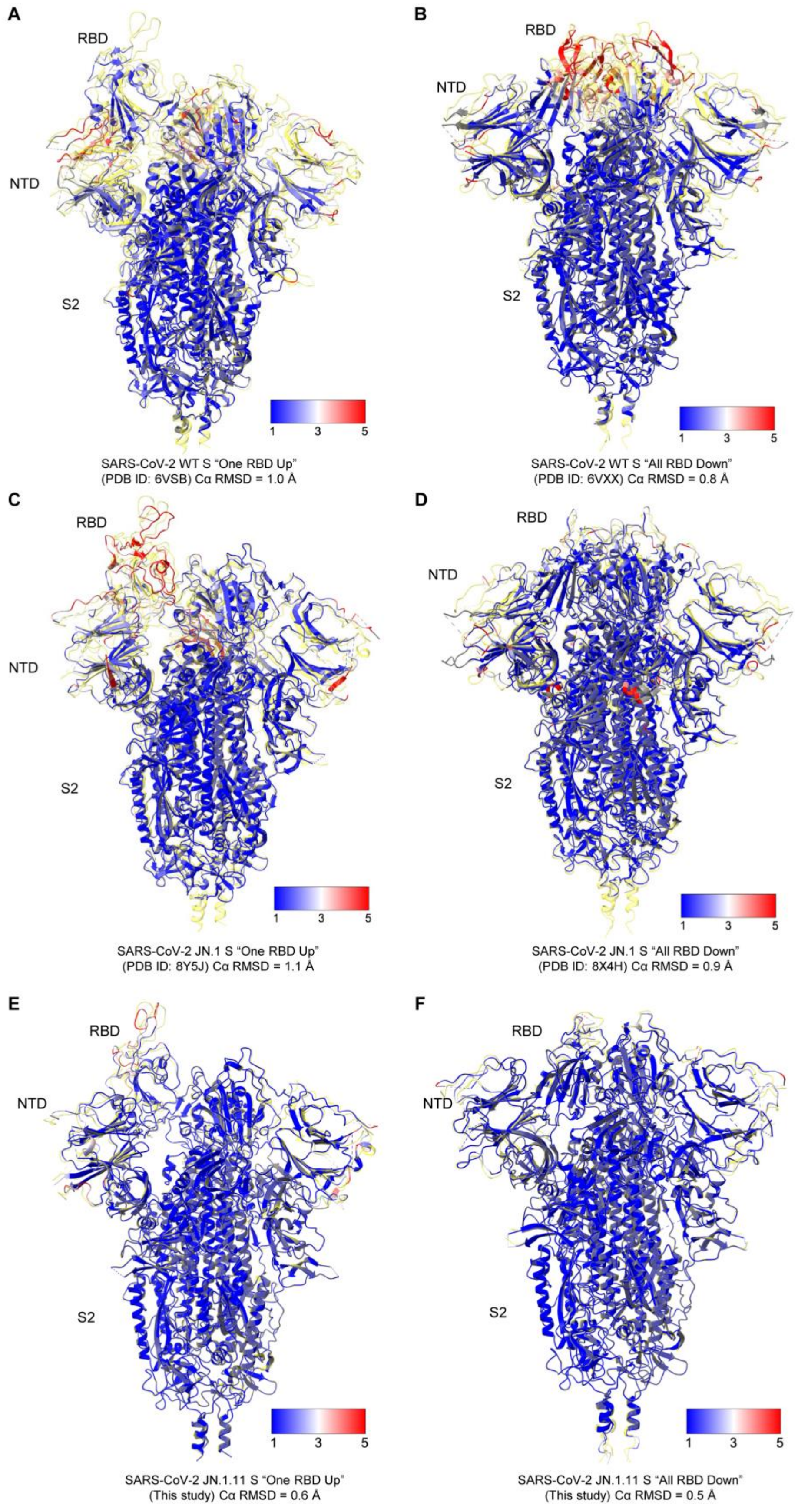
A
RBD
NTD
S2
1
3
5
SARS-CoV-2 WT S "One RBD Up"
(PDB ID: 6VSB) Cα RMSD = 1.0 Å
B
RBD
NTD
S2
1
3
5
SARS-CoV-2 WT S "All RBD Down"
(PDB ID: 6VXX) Cα RMSD = 0.8 Å
C
RBD
NTD
S2
1
3
5
SARS-CoV-2 JN.1 S "One RBD Up"
(PDB ID: 8Y5J) Cα RMSD = 1.1 Å
D
RBD
NTD
S2
1
3
5
SARS-CoV-2 JN.1 S "All RBD Down"
(PDB ID: 8X4H) Cα RMSD = 0.9 Å
E
RBD
NTD
S2
1
3
5
SARS-CoV-2 JN.1.11 S "One RBD Up"
(This study) Cα RMSD = 0.6 Å
F
RBD
NTD
S2
1
3
5
SARS-CoV-2 JN.1.11 S "All RBD Down"
(This study) Cα RMSD = 0.5 Å

Figure S4. Structural superimposition of SARS-CoV-2 WT, JN.1, and JN.1.11 spike protein with KP.3.1.1 spike protein. KP.3.1.1 spike proteins are colored light yellow. Structural differences of SARS-CoV-2 WT, JN.1, and JN.1.11 spike are color-coded by their root mean square deviation (RMSD) (Å). RBD, NTD, and S2 are labeled, related to Figure 3.

(A, C, E) Structural alignment of the KP.3.1.1 “One RBD Up” spike structure to SARS-CoV-2 WT (PDB ID: 6VSB), JN.1 (PDB ID: 8Y5J), and JN.1.11 (this study), respectively.

(B, D, F) Structural alignment of the KP.3.1.1 “All RBD Down” spike structure to SARS-CoV-2 WT (PDB ID: 6VXX), JN.1 (PDB ID: 8X4H), and JN.1.11 (this study), respectively.

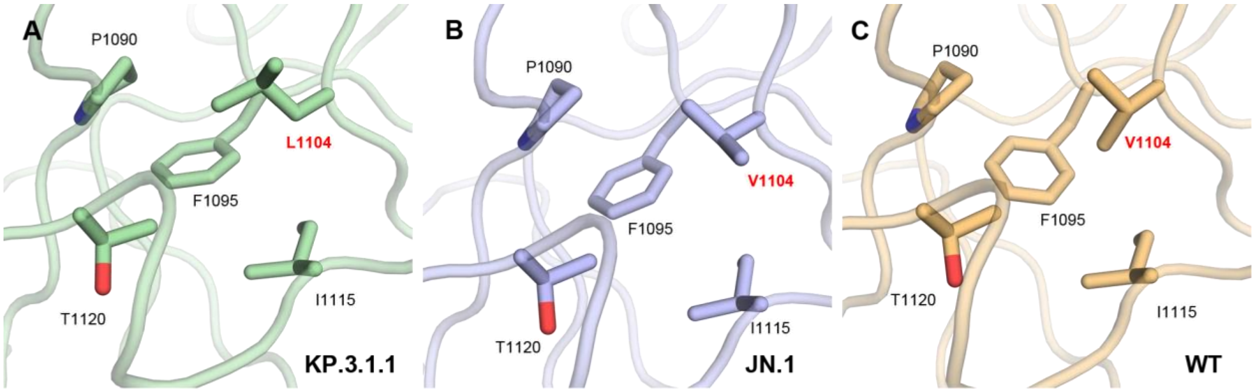

Figure S5. Zoomed-in view of V/L1104 in WT, JN.1, and KP.3.1.1 spikes, related to Figure 3.

(A-C) L1104 or V1104 is surrounded by a hydrophobic pocket formed by P1090, F1095, I1115, and T1120 in KP3.1.1., JN.1, and WT spike proteins.

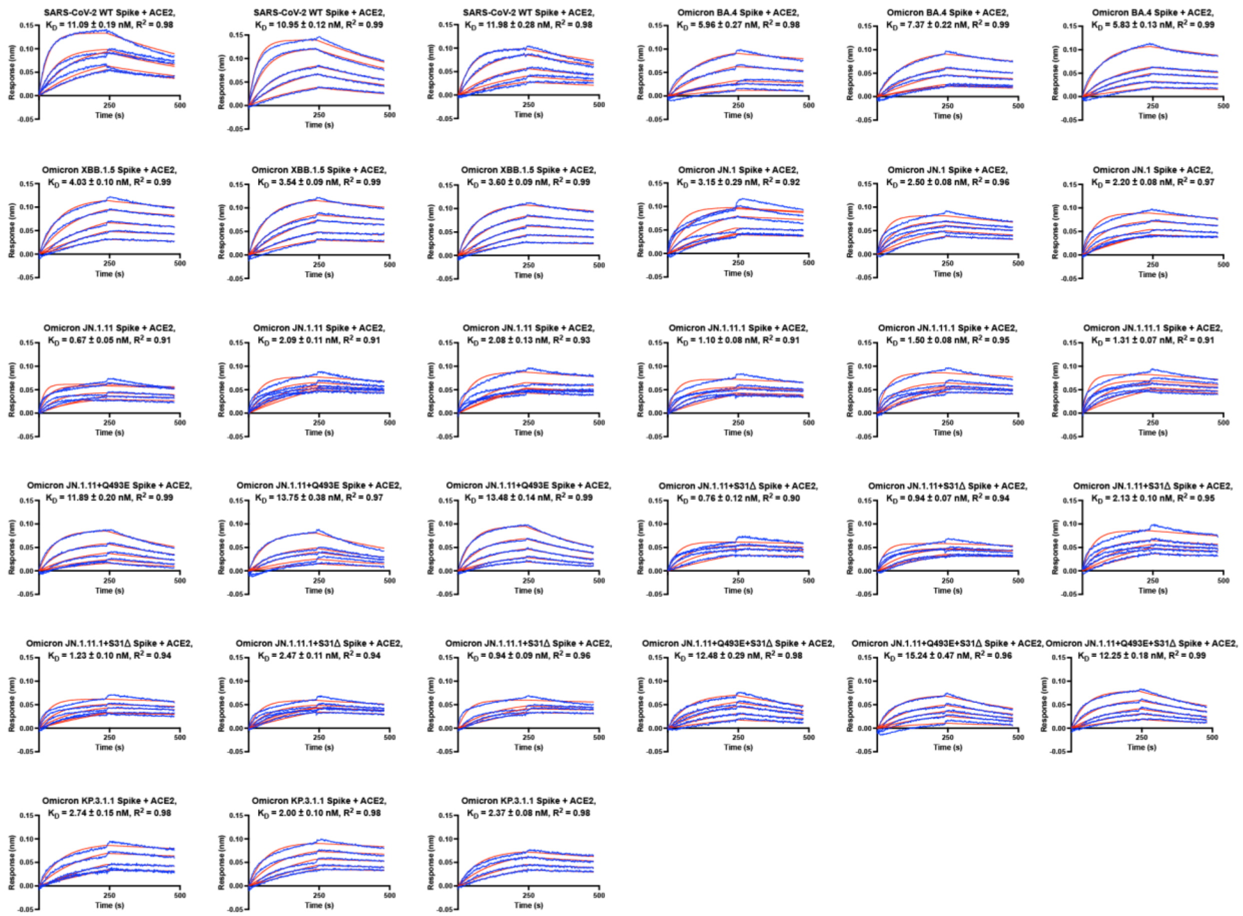

Figure S6. Sensorgrams for binding of various spikes to hACE2, related to Figure 4.

Binding kinetics of different spike proteins against hACE2 were measured by biolayer interferometry (BLI) in triplicate. The Y-axis represents the response. Red lines represent the response curve, and blue lines represent a 1:1 binding model. Binding kinetics were measured for hACE2 concentrations (6.25, 12.5, 25, 50, and 100 nM). Dissociation constant (K_D) with standard deviation and coefficient of determination (R^2) are indicated.

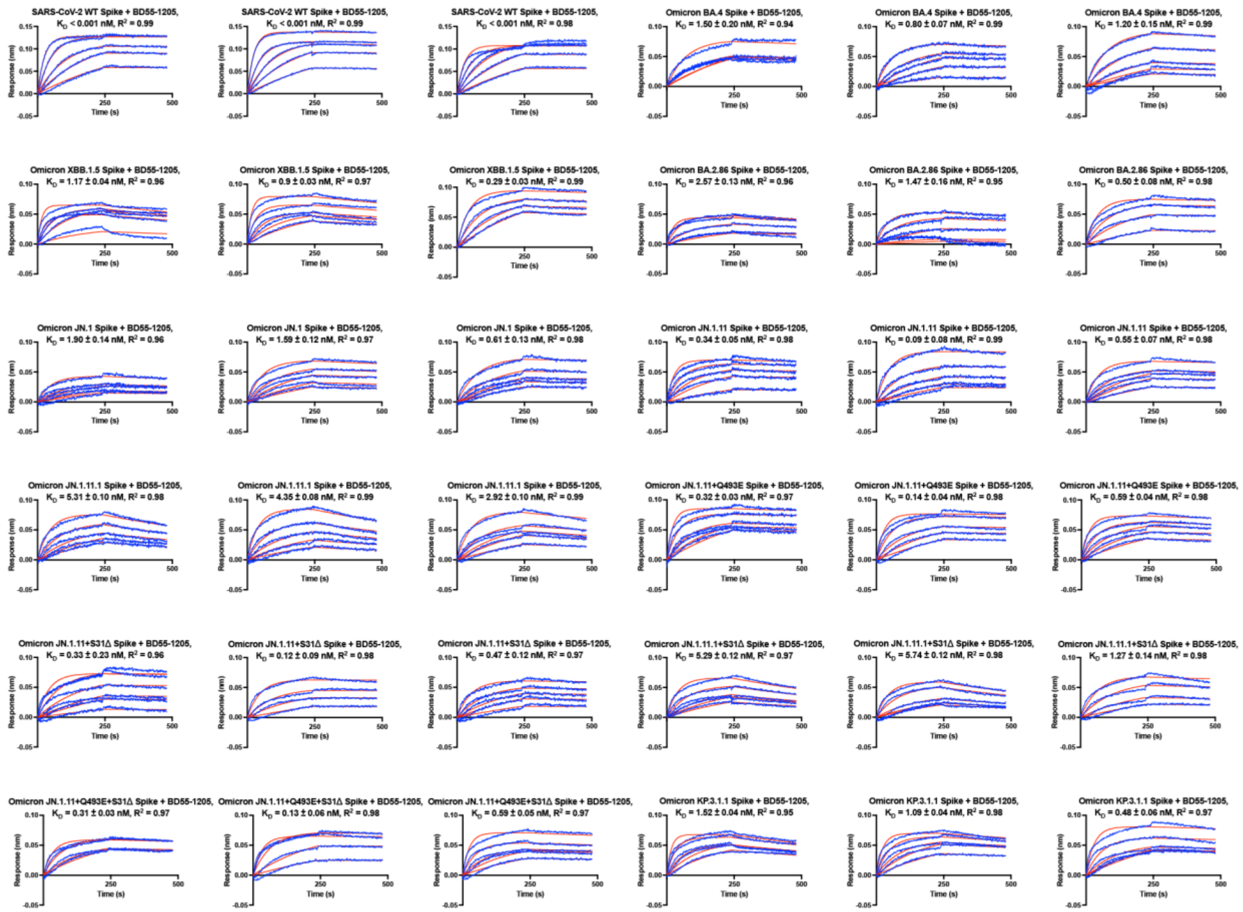

Figure S7. Sensorgrams for binding of various spikes to BD55-1205 Fab, related to Figure 5.

Binding kinetics of different spike proteins against BD55-1205 Fab were measured by biolayer interferometry (BLI) in triplicate. The Y-axis represents the response. Red lines represent the response curve, and blue lines represent a 1:1 binding model. Binding kinetics were measured for Fab concentrations (6.25, 12.5, 25, 50, and 100 nM). Dissociation constant (K_D) with standard deviation and coefficient of determination (R^2) are indicated.

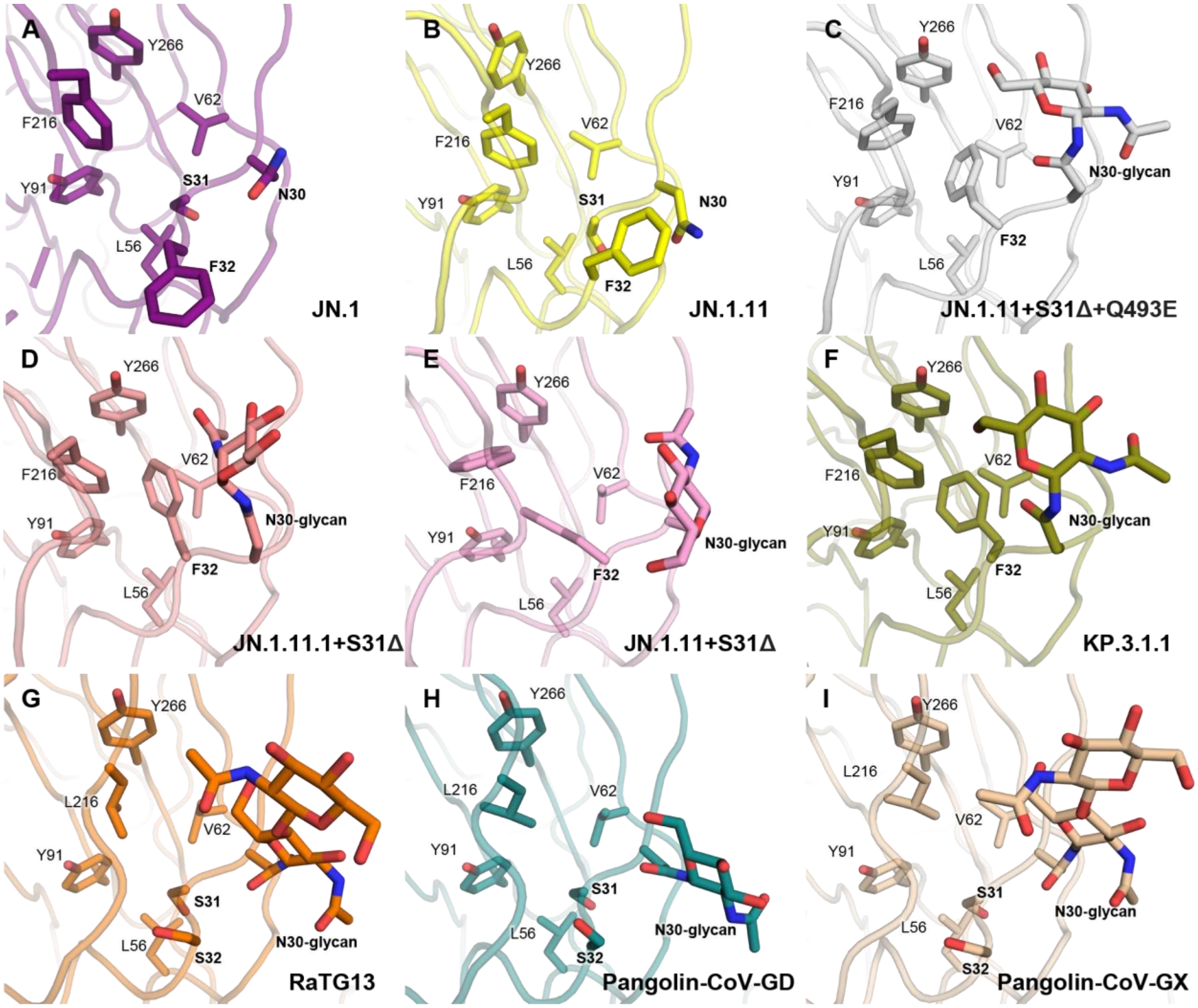

Figure S8. Zoomed-in view of N30 in various spikes, related to Figure 5.

(A-I) Molecular details of side-chain conformations including N30 with glycan, S31, F/S32, L56, V62, Y91, L/F216, and Y266 of JN.1 (purple), JN.1.11 (yellow), JN.1.11+S31Δ+Q493E (grey), JN.1.11.1+S31Δ (flesh), JN.1.11+S31Δ (pink), KP.3.1.1 (olive), RaTG13 (orange), Pangolin-CoV-GD (teal), and Pangolin-CoV-GX (wheat), respectively.

A

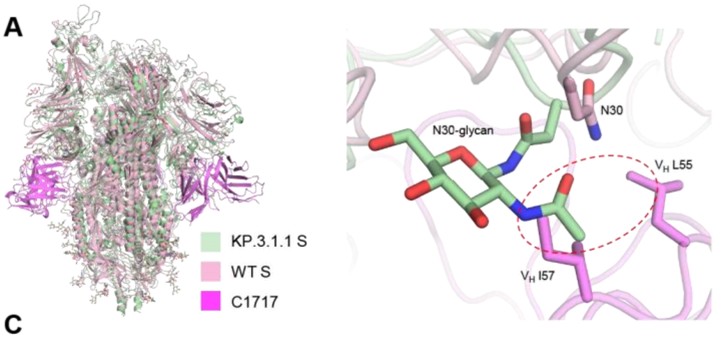

B

Viral Strain	S31Δ	Binding to C1717 Fab
WT	No	Yes
JN.1.11	No	Yes
JN.1.11.1	No	Yes
JN.1.11+Q493E	No	Yes
JN.1.11+S31Δ	Yes	No
JN.1.11.1+S31Δ	Yes	No
JN.1.11+S31Δ+Q493E	Yes	No
KP.3.1.1	Yes	No

C

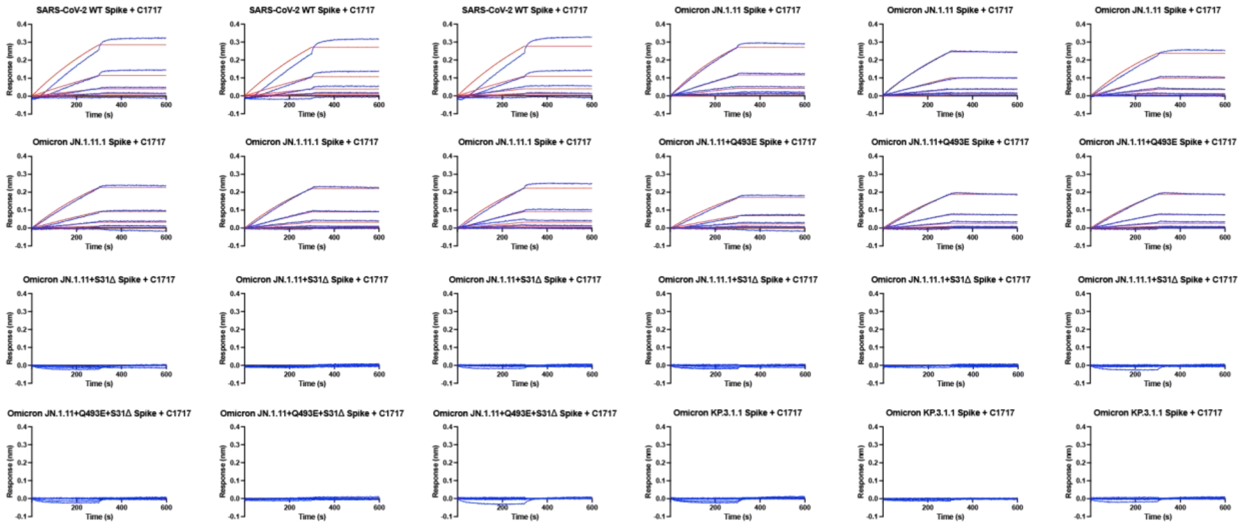

Figure S9. Structural alignment and binding between various spikes and NTD site vi antibody C1717, related to Figure 5.

(A) Left, superimposed structures of KP.3.1.1 S and WT S/C1717 complex. KP.3.1.1 S, WT S, and C1717 are colored in light green, pink, and magenta, respectively. Right, a zoomed-in view of the NTD shows that N30 glycan may clash with C1717 V_H L55 and V_H I57. The red dashed circle highlights the potential clash.

(B) Correlation of having S31Δ and binding to C1717 in various spikes.

(C) Sensorgrams for binding of various spikes to C1717 Fab. Binding kinetics of different spike proteins against BD55-1205 Fab were measured by biolayer interferometry (BLI) in triplicate. The Y-axis represents the response. Red lines represent the response curve, and blue lines represent a 1:1 binding model. Binding kinetics were measured for spike concentrations (3.7, 11.1, 33.3, 100, and 300 nM).

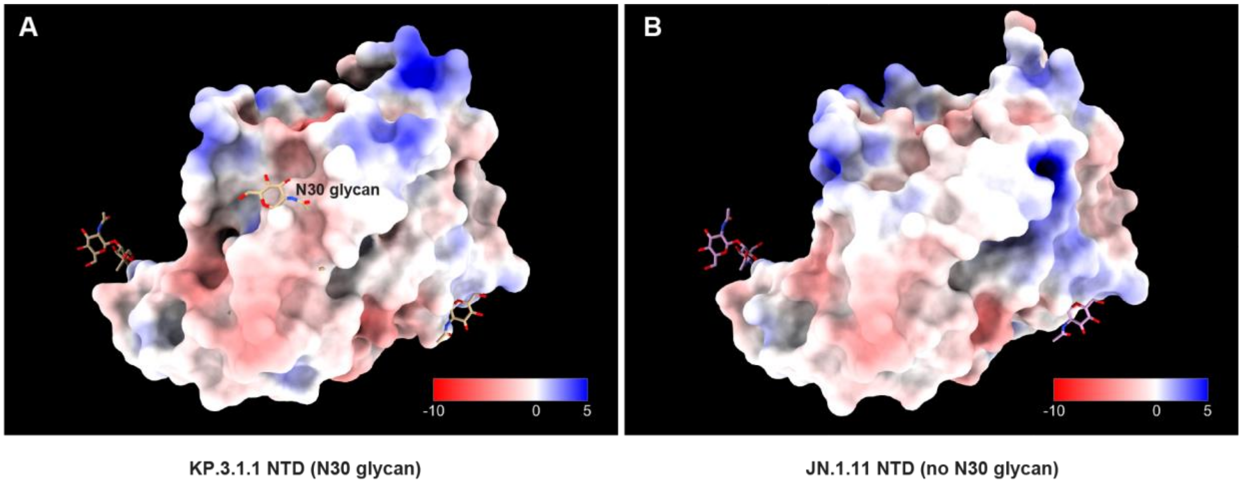

Figure S10 Electrostatic potential surfaces of spike NTD, related to Figure 5.

(A-B) The electric potential on the surface of NTD of KP.3.1.1 and JN.1.11 at pH 7.0, respectively. The position of the N30 glycan in KP.3.1.1 is labeled. The potential is visualized by colorings according to its sign: positive (blue), negative (red), and neutral (white); the intensity of the colors corresponds to the *kT/e* scale (shown on the bottom right) in the range -10 to +5 *kT/e* [J/C] (*k*—Boltzmann constant [J/K], *T*—absolute temperature [K], *e*—charge of the proton [C]; 1 *kT/e* [J/C] = 26.7 mV at 37 °C).

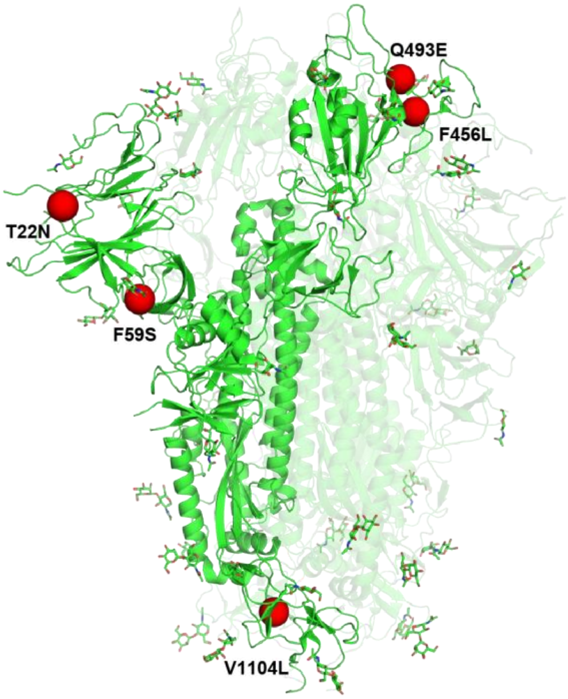

Figure S11. Mutations (red) on XEC in the context of JN.1 S structure (green) (PDB ID: 8X4H), related to Figure 6.

Table S1. Cryo-EM data collection, processing, and model building statistics.

Sample	KP.3.1.1 Spike HexaPro + hACE2		KP.3.1.1 Spike HexaPro				JN.1.11 S31del Q493E Spike HexaPro			JN.1.11 Spike HexaPro			JN.1.11 + S31del Spike HexaPro			JN.1.11.1+S31del Spike HexaPro		
Map	Local refine RBD+hACE2	Global refine S with two hACE2	Local refine RBD+NTD	One RBD Up	All RBD Down	Flexible with RBD missing	One RBD Up	All RBD Down	Flexible with RBD missing	One RBD Up	All RBD Down	Flexible with RBD missing	One RBD Up	All RBD Down	Flexible with RBD missing	One RBD Up	All RBD Down	Flexible with RBD missing
EMDB	48146	48147	48148	48149	48150	49904	48151	48152	49905	48153	48155	49906	48156	48157	49907	48158	48159	49908
PDB	9ELE	9ELF	9ELG	9ELH	9ELI		9ELJ	9ELK		9ELL	9ELM		9ELN	9ELO		9ELP	9ELQ	
**Data collection & processing**																		
Microscope/ Detector	Glacios/Falcon4																	
Voltage (kV)	200																	
Magnification	190,000																	
Recording mode	Counting																	
Pixel size (Å)	0.718																	
Total dose (e-/Å^2)	44.84																	
Defocus range (μm)	-0.8 to -1.7																	
No. of movie micrographs	4334		4336				4716			3680			4010			7672		
No. of molecular projection images in map	440,016	131,224	98,127	55,445	40,554	61,797	94,590	41,085	39,310	39,569	42,460	30,927	63,034	59,178	40,728	53,372	112,912	113,266
Symmetry	C1	C1	C1	C1	C3	C1	C1	C3	C1	C1	C3	C1	C1	C3	C1	C1	C3	C1
Map pixel size (Å)	0.718																	
Map resolution (FSC 0.143; Å)	3.16	2.88	3.71	2.98	2.82	2.98	2.92	2.82	3.15	2.93	2.68	2.99	2.93	2.74	3.00	2.94	2.64	2.82
Map sharpening B-factor (Å^2)	-107.1	-66.1	-110.6	-58.9	-72.6	-62.3	-65.5	-68.0	-52.1	-48.7	-62.7	-45.7	-58.2	-72.1	-52.6	-53.3	-76.2	-65.7
**Structure building and validation**																		
Model composition																		
Non-hydrogen atoms	6,529	35,225	4,255	25,575	25,509		25,608	25,347		25,627	25,374		25,597	25,578		23,626	25,527	
Protein residues	780	4,334	510	3,160	3,153		3,166	3,129		3,165	3,132		3,163	3,156		2,925	3,156	
Ligands	NAG:12/BMA:1	NAG:58/BMA:6	NAG:11	NAG:52/BMA:7	NAG:51/BMA:6		NAG:50/BMA:7	NAG:51/BMA:6		NAG:52/BMA:7	NAG:51/BMA:6		NAG:52/BMA:7	NAG:54/BMA:6		NAG:48/BMA:7	NAG:51/BMA:6	
RMSD bond length (Å)/angles (°)	0.007/ 1.203	0.007/ 1.23	0.006/ 1.189	0.006/ 1.129	0.017/ 1.175		0.006/ 1.048	0.007/ 1.129		0.006/ 1.079	0.009/ 1.381		0.012/ 1.37	0.008/ 1.29		0.009/ 1.21	0.008/ 1.33	
MolProbity score	1.07	1.38	1.29	1.50	1.12		1.34	1.38		1.48	1.20		1.66	1.47		1.27	1.57	
Clash score	2.83	6.92	2.64	4.85	1.59		3.05	4.20		4.73	4.01		6.46	5.35		2.74	7.06	
Ramachandran outliers/allowed/favored (%)	0/ 1.03/ 98.97	0/ 1.94/ 98.06	0/ 3.61/ 96.39	0/ 3.66/ 96.34	0/ 3.32/ 96.68		0/ 3.62/ 96.38	0/ 3.01/ 96.99		0/ 3.56/ 96.44	0/ 2.04/ 97.96		0.03/ 4.37/ 95.6	0/ 3.08/ 96.92		0.03/ 3.25/ 96.72	0/ 3.08/ 96.92	
Rotamer outliers (%)	0	0.13	0	0.07	0		0	0		0.07	0		0.14	0.11		0.16	0.11	
Cβ outliers (%)	0	0.32	0	0	0		0	0		0	0		0.14	0.1		0.07	0.3	
d FSC model (0.5; Å)	3.3	3.1	3.9	3.2	3.0		3.2	3.1		3.2	2.9		3.2	2.9		3.1	2.8	

Data S1. Sarbecoviruses spike sequence alignment

```
CLUSTAL O(1.2.4) multiple sequence alignment


SARS-CoV-2Wuhan_MN908947                M-FVFLVLLPLVSSQ----CVNLT-TRTQLPPAY--TNSFTRGVYYPDKVFRSSVLHSTQ
SARS-CoV-2delta_QUD52764.1              M-FVFLVLLPLVSSQ----CVNLR-TRTQLPPAY--TNSFTRGVYYPDKVFRSSVLHSTQ
SARS-CoV-2_BA1_UFO69279.1               M-FVFLVLLPLVSSQ----CVNLT-TRTQLPPAY--TNSFTRGVYYPDKVFRSSVLHSTQ
SARS-CoV-2_BA2_UJP23605.1               M-FVFLVLLPLVSSQ----CVNLI-TRTQ---SY--TNSFTRGVYYPDKVFRSSVLHSTQ
SARS-CoV-2_BA5_UOZ45804.1               M-FVFLVLLPLVSSQ----CVNLI-TRTQ---SY--TNSFTRGVYYPDKVFRSSVLHSTQ
SARS-CoV-2_BQ1.1_UWM38596.1             M-FVFLVLLPLVSSQ----CVNLI-TRTQ---SY--TNSFTRGVYYPDKVFRSSVLHSTQ
SARS-CoV-2_EG.5.1_WGM84363.1            M-FVFLVLLPLVSSQ----CVNLI-TRTQ---SY--TNSFTRGVYYPDKVFRSSVLHSTH
SARS-CoV-2_XBB.1.5_UZG29433.1           M-FVFLVLLPLVSSQ----CVNLI-TRTQ---SY--TNSFTRGVYYPDKVFRSSVLHSTQ
SARS-CoV-2_JN.1_WPF38074.1              M-FVFLVLLPLVSSQ----CVMPLFNLITTTQSY--TNSFTRGVYYPDKVFRSSVLHLTQ
SARS-CoV-2_KP.3.1.1                     M-FVFLVLLPLVSSQ----CVMPLFNLITTTQSY--TN-FTRGVYYPDKVFRSSVLHLTQ
Pangolin_GD-consensus_Lam2020           MLFFFFLHFALVNSQ----CVNLT-GRAAIQPSF--TNSSQRGVYYPDTIFRSNTLVLSQ
RaTG13_MN996532                         M-FVFLVLLPLVSSQ----CVNLT-TRTQLPPAY--TNSSTRGVYYPDKVFRSSVLHLTQ
Pangolin_GX-P2V_EPI_ISL_410542          M-FVFLFVLPLVSSQ----CVNLT-TRTGIPPGY--TNSSTRGVYYPDKVFRSSILHLTQ
SARS-CoV-1_Urbani_HP03L_AY278741        M-FIFLLFLTLTSGSDLDRCTTFDD---VQAPNYTQHTSSMRGVYYPDEIFRSDTLYLTQ
SARS-CoV-1_HGZ8L1-A_HP03E_AY394981      M-FIFLLFLTLTSGSDLDRCTTFDD---VQAPNYTQHTSSMRGVYYPDEIFRSDTLYLTQ
SARS-CoV-1_GZ-C_HP03L_AY394979          M-FIFLLFLTLTSGSDLDRCTTFDD---VQAPNYTQHTSSMRGVYYPDEIFRSDTLYLTQ
SARS-CoV-1_Sino1-11_HP03L_AY485277      M-FIFLLFLTLTSGSDLDRCTTFDD---VQAPNYTQHTSSMRGVYYPDEIFRSDTLYLTQ
SARS-CoV-1_Sin852_HP03L_AY559082        M-FIFLLFLTLTSGSDLDRCTTFDD---VQAPNYTQHTSSMRGVYYPDEIFRSDTLYLTQ
SARS-CoV-1_SZ1_PC03_AY304489            M-FIFLLFLTLTSGSDLDRCTTFDD---VQAPNYTQHTSSMRGVYYPDEIFRSDTLYLTQ
SARS-CoV-1_GD03T0013_HP04_AY525636      M-FIFLLFLTLTSGSDLDRCTTFDD---VQAPNYTQHTSSMRGVYYPDEIFRSDTLYLTQ
SARS-CoV-1_GZ0402_HP04_AY613947         M-FIFLLFLTLTSGSDLDRCTTFDD---VQAPNYTQHTSSMRGVYYPDEIFRSDTLYLTQ
SARS-CoV-1_PC4-127_PC04_AY613951        M-FIFLLFLTLTSGSDLDRCTTFDD---VQAPNYTQHTSSMRGVYYPDEIFRSDTLYLTQ
SARS-CoV-1_PC4-137_PC04_AY627045        M-FIFLLFLTLTSGSDLDRCTTFDD---VQAPNYTQHTSSMRGVYYPDEIFRSDTLYLTQ
SARS-CoV-1_PC4-13_PC04_AY613948         M-FIFLLFLTLTSGSDLDRCTTFDD---VQAPNYTQHTSSMRGVYYPDEIFRSDTLYLTQ
WIV1_KF367457                           MKLLVLVFATLVSSYTIEKCLDFDD---RTPPANTQFLSSHRGVYYPDDIFRSNVLHLVQ
Rs7327_KY417151                         MKLLVLVFATLVSSYTIEKCLDFDD---RTPPANTQFLSSHRGVYYPDDIFRSNVLHLVQ
Rs4231_KY417146                         M-FIFLFFLTLTSGSDLESCTTFDD---VQAPNYPQHSSSRRGVYYPDEIFRSDTLYLTQ
RsSHC014_KC881005                       MKLLVLVFATLVSSYTIEKCLDFDD---RTPPANTQFLSSHRGVYYPDDIFRSNVLHLVQ
Rs4084_KY417144                         MKLLVLVFATLVSSYTIEKCLDFDD---RTPPANTQFLSSHRGVYYPDDIFRSNVLHLVQ
BtKY72_KY352407                         MKF-FILLSLLSFTTAQEGCGILS---NKSNPALTQYFSSRRGFYYFDDTFRSSVRVLTT
                                        * : .:.   *        *                     **.** *  ***.

SARS-CoV-2Wuhan_MN908947                DLFLPFFSNVTWFHAIHVSGTNGTKRFDNPVLPFNDGVYFASTEKSNIIRGWIFGTTLDS
SARS-CoV-2delta_QUD52764.1              DLFLPFFSNVTWFHAIHVSGTNGTTRFDNPVLPFNDGVYFASTEKSNIIRGWIFGTTLDS
SARS-CoV-2_BA1_UFO69279.1               DLFLPFFSNVTWFHVI--SGTNGTKRFDNPVLPFNDGVYFASIEKSNIIRGWIFGTTLDS
SARS-CoV-2_BA2_UJP23605.1               DLFLPFFSNVTWFHAIHVSGTNGTKRFDNPVLPFNDGVYFASTEKSNIIRGWIFGTTLDS
SARS-CoV-2_BA5_UOZ45804.1               DLFLPFFSNVTWFHAI--SGTNGIKRFDNPVLPFNDGVYFASTEKSNIIRGWIFGTTLDS
SARS-CoV-2_BQ1.1_UWM38596.1             DLFLPFFSNVTWFHAI--SGTNGTKRFDNPVLPFNDGVYFASTEKSNIIRGWIFGTTLDS
SARS-CoV-2_EG.5.1_WGM84363.1            DLFLPFFSNVTWFHAIHVSGTNGTKRFDNPALPFNDGVYFASTEKSNIIRGWIFGTTLDS
SARS-CoV-2_XBB.1.5_UZG29433.1           DLFLPFFSNVTWFHAIHVSGTNGTKRFDNPALPFNDGVYFASTEKSNIIRGWIFGTTLDS
SARS-CoV-2_JN.1_WPF38074.1              DLFLPFFSNVTWFHAI--SGTNGTKRFDNPVLPFNDGVYFASTEKSNIIRGWIFGTTLDS
SARS-CoV-2_KP.3.1.1                     DLFLPFFSNVTWFHAI--SGTNGTKRFDNPVLPFNDGVYFASTEKSNIIRGWIFGTTLDS
Pangolin_GD-consensus_Lam2020           GYFLPFYSNVSWYYALTKT-NSAEKRVDNPVLDFKDGIYFAATEKSNIVRGWIFGTTLDN
RaTG13_MN996532                         DLFLPFFSNVTWFHAIHVSGTNGIKRFDNPVLPFNDGVYFASTEKSNIIRGWIFGTTLDS
Pangolin_GX-P2V_EPI_ISL_410542          DLFLPFFSNVTWFNTIHLNYQGGFKKFDNPVLPFNDGVYFASTEKSNIIRGWIFGTTLDA
SARS-CoV-1_Urbani_HP03L_AY278741        DLFLPFYSNVTGFHTIN-------HTFGNPVIPFKDGIYFAATEKSNVVRGWVFGSTMNN
SARS-CoV-1_HGZ8L1-A_HP03E_AY394981      DLFLPFYSNVTGFHTIN--------RFDNPVIPFKDGIYFAATEKSNVVRGWVFGSTMNN
SARS-CoV-1_GZ-C_HP03L_AY394979          DLFLPFYSNVTGFHTIN-------HTFGNPVIPFKDGIYFAATEKSNVVRGWVFGSTMNN
SARS-CoV-1_Sino1-11_HP03L_AY485277      DLFLPFYSNVTGFHTIN-------HTFGNPVIPFKDGIYFAATEKSNVVRGWVFGSTMNN
SARS-CoV-1_Sin852_HP03L_AY559082        DLFLPFYSNVTGFHTIN-------HTFGNPVIPFKDGIYFAATEKSNVVRGWVFGSTMNN
SARS-CoV-1_SZ1_PC03_AY304489            DLFLPFYSNVTGFHTIN-------HTFDNPVIPFKDGIYFAATEKSNVVRGWVFGSTMNN
SARS-CoV-1_GD03T0013_HP04_AY525636      DLFLPFYSNVTGFHTIN-------HTFDDPVIPFKDGIYFAATEKSNVVRGWVFGSTMNN
SARS-CoV-1_GZ0402_HP04_AY613947         DLFLPFYSNVTGFHTIN-------HTFDNPVIPFKDGIYFAATEKSNVVRGWVFGSTMNN
SARS-CoV-1_PC4-127_PC04_AY613951        DLFLPFYSNVTGFHTIN-------HTFDNPVIPFKDGIYFAATEKSNVVRGWVFGSTMNN
SARS-CoV-1_PC4-137_PC04_AY627045        DLFLPFYSNVTGFHTIN-------HTFDNPVIPFKDGIYFAATEKSNVVRGWVFGSTMNN
SARS-CoV-1_PC4-13_PC04_AY613948         DLFLPFYSNVTGFHTIN-------HTFDNPVIPFKDGIYFAATEKSNVVRGWVFGSTMNN
WIV1_KF367457                           DHFLPFDSNVTRFITFG-------LNFDNPIIPFKDGIYFAATEKSNVIRGWVFGSTMNN
Rs7327_KY417151                         DHFLPFDSNVTRFITFG-------LNFDNPIIPFRDGIYFAATEKSNVIRGWVFGSTMNN
Rs4231_KY417146                         DLFLPFYSNVTGFHTIN-------HRFDNPVIPFKDGVYFAATEKSNVVRGWVFGSTMNN
RsSHC014_KC881005                       DHFLPFDSNVTRFITFG-------LNFDNPIIPFRDGIYFAATEKSNVIRGWVFGSTMNN
Rs4084_KY417144                         DHFLPFDSNVTRFITFG-------LNFDNPIIPFRDGIYFAATEKSNVIRGWVFGSTMNN
BtKY72_KY352407                         GYFLPFNSNLTGYSSRN-SVTGRLIQFDNPNIPFKDGLYFAATERSNVIRGWIFGSTLDN
                                        . **** **:: :             ..:* : *.**:***: *:**::***:**:*::

SARS-CoV-2Wuhan_MN908947                KTQSLLIVNNATNVVIKVCEFQFCNDPFLGVYYHKNNKSWMESEFRVYSSANNCTFEYVS
SARS-CoV-2delta_QUD52764.1              KTQSLLIVNNATNVVIKVCEFQFCNDPFLGVYYHKNNKSWMES--GVYSSANNCTFEYVS
SARS-CoV-2_BA1_UFO69279.1               KTQSLLIVNNATNVVIKVCEFQFCNDPFLD---HKNNKSWMESEFRVYSSANNCTFEYVS
SARS-CoV-2_BA2_UJP23605.1               KTQSLLIVNNATNVVIKVCEFQFCNDPFLDVYYHKNNKSWMESEFRVYSSANNCTFEYVS
SARS-CoV-2_BA5_UOZ45804.1               KTQSLLIVNNATNVVIKVCEFQFCNDPFLDVYYHKNNKSWMESEFRVYSSANNCTFEYVS
SARS-CoV-2_BQ1.1_UWM38596.1             KTQSLLIVNNATNVVIKVCEFQFCNDPFLDVYYHKNNKSWMESEFRVYSSANNCTFEYVS
```

```
SARS-CoV-2_EG.5.1_WGM84363.1            KTQSLLIVNNATNVVIKVCEFQFCNDPFLDVY-QKNNKSWMESEFRVYSSANNCTFEYVS
SARS-CoV-2_XBB.1.5_UZG29433.1           KTQSLLIVNNATNVVIKVCEFQFCNDPFLDVY-QKNNKSWMESEFRVYSSANNCTFEYVS
SARS-CoV-2_JN.1_WPF38074.1              KTQSLLIVNNATNVFIKVCEFQFCNDPFLDV-YHKNNKSWMESESGVYSSANNCTFEYVS
SARS-CoV-2_KP.3.1.1                     KTQSLLIVNNATNVFIKVCEFQFCNDPFLDV-YHKNNKSWMESESGVYSSANNCTFEYVS
Pangolin_GD-consensus_Lam2020           TSQSLLIVNNATNVIIKVCNFQFCYDPYLSGYYHN-NKTWSTREFAVYSSYANCTFEYVS
RaTG13_MN996532                         KTQSLLIVNNATNVVIKVCEFQFCNDPFLGVYYHKNNKSWMESEFRVYSSANNCTFEYVS
Pangolin_GX-P2V_EPI_ISL_410542          RTQSLLIVNNATNVVIKVCEFQFCTDPFLGVYYHNNNKTWVENEFRVYSSANNCTFEYIS
SARS-CoV-1_Urbani_HP03L_AY278741        KSQSVIIINNSTNVVIRACNFELCDNPFFAVSKPMGT----QTHTMIFDNAFNCTFEYIS
SARS-CoV-1_HGZ8L1-A_HP03E_AY394981      KSQSVIIINNSTNVVIRACNFELCDNPFFAVSKPMGT----QTHTMIFDNAFNCTFEYIS
SARS-CoV-1_GZ-C_HP03L_AY394979          KSQSVIIINNSTNVVIRACNFELCDNPFFAVSKPMGT----QTHTMIFDNAFNCTFEYIS
SARS-CoV-1_Sino1-11_HP03L_AY485277      KSQSVIIINNSTNVVIRACNFELCDNPFFAVSKPMGT----QTHTMIFDNAFNCTFEYIS
SARS-CoV-1_Sin852_HP03L_AY559082        KSQSVIIINNSTNVVIRACNFELCDNPFFAVSKPMGT----QTHTMIFDNAFNCTFEYIS
SARS-CoV-1_SZ1_PC03_AY304489            KSQSVIIINNSTNVVIRACNFELCDNPFFAVSKPMGT----QTHTMIFDNAFNCTFEYIS
SARS-CoV-1_GD03T0013_HP04_AY525636      KSQSVIIINNSTNVVIRACNFELCDNPFFVVSKPMGT----RTHTMIFDNAFNCTFEYIS
SARS-CoV-1_GZ0402_HP04_AY613947         KSQSVIIINNSTNVVIRACNFELCDNPFFVVSKPMGT----RTHTMIFDNAFNCTFEYIS
SARS-CoV-1_PC4-127_PC04_AY613951        KSQSVIIINNSTNVVIRACNFELCDNPFFVVSKPMGT----RTHTMIFDNAFNCTFEYIS
SARS-CoV-1_PC4-137_PC04_AY627045        KSQSVIIINNSTNVVIRACNFELCDNPFFVVSKPMGT----RTHTMIFDNAFNCTFEYIS
SARS-CoV-1_PC4-13_PC04_AY613948         KSQSVIIINNSTNVVIRACNFELCDNPFFVVSKPMGT----RTHTMIFDNAFNCTFEYIS
WIV1_KF367457                           KSQSVIIMNNSTNLVIRACNFELCDNPFFVVLKSNNT----QIPSYIFNNAFNCTFEYVS
Rs7327_KY417151                         KSQSVIIMNNSTNLVIRACNFELCDNPFFVVLKSNNT----QIPSYIFNNAFNCTFEYVS
Rs4231_KY417146                         KSQSVIIINNSTNVVIRACNFELCDNPFFAVSKPTGT----QTHTMIFDNAFNCTFEYIS
RsSHC014_KC881005                       KSQSVIIMNNSTNLVIRACNFELCDNPFFVVLKSNNT----QIPSYIFNNAFNCTFEYVS
Rs4084_KY417144                         KSQSVIIMNNSTNLVIRACNFELCDNPFFVVLKSNNT----QIPSYIFNNAFNCTFEYVS
BtKY72_KY352407                         TTQSAVLFNNGTHIVINVCNFYFCQDPMLAVANGSHFKSWV------FLNATNCTYNRV-
                                         :** ::.**.*::.*..*:* :* :* :                  : .  ***:: : 

SARS-CoV-2Wuhan_MN908947                QPFLMDLEGKQGNFKNLREFVFKNIDGYFKIYSKHTPINL--VRDLPQGFSALEPLVDLP
SARS-CoV-2delta_QUD52764.1              QPFLMDLEGKQGNFKNLREFVFKNIDGYFKIYSKHTPINL--VRDLPQGFSALEPLVDLP
SARS-CoV-2_BA1_UFO69279.1               QPFLMDLEGKQGNFKNLREFVFKNIDGYFKIYSKHTPIIVREPEDLPQGFSALEPLVDLP
SARS-CoV-2_BA2_UJP23605.1               QPFLMDLEGKQGNFKNLREFVFKNIDGYFKIYSKHTPINL--GRDLPQGFSALEPLVDLP
SARS-CoV-2_BA5_UOZ45804.1               QPFLMDLEGKQGNFKNLREFVFKNIDGYFKIYSKHTPINL--GRDLPQGFSALEPLVDLP
SARS-CoV-2_BQ1.1_UWM38596.1             QPFLMDLEGKQGNFKNLREFVFKNIDGYFKIYSKHTPINL--GRDLPQGFSALEPLVDLP
SARS-CoV-2_EG.5.1_WGM84363.1            QPFLMDLEGKEGNFKNLREFVFKNIDGYFKIYSKHTPINL--ERDLPQGFSALEPLVDLP
SARS-CoV-2_XBB.1.5_UZG29433.1           QPFLMDLEGKEGNFKNLREFVFKNIDGYFKIYSKHTPINL--ERDLPQGFSALEPLVDLP
SARS-CoV-2_JN.1_WPF38074.1              QPFLMDLEGKQGNFKNLREFVFKNIDGYFKIYSKHTPIIGR---DFPQGFSALEPLVDLP
SARS-CoV-2_KP.3.1.1                     QPFLMDLEGKQGNFKNLREFVFKNIDGYFKIYSKHTPIIGR---DFPQGFSALEPLVDLP
Pangolin_GD-consensus_Lam2020           KSFMLDIAGKSGLFDTLREFVFRNVDGYFKIYSKYTPVNVN--SNLPIGFSALEPLVEIP
RaTG13_MN996532                         QPFLMDLEGKQGNFKNLREFVFKNIDGYFKIYSKHTPINL--VRDLPPGFSALEPLVDLP
Pangolin_GX-P2V_EPI_ISL_410542          QPFLMDLEGKQGNFKNLREFVFKNVDGYFKIYSKHTPIDL--VRDLPRGFAALEPLVDLP
SARS-CoV-1_Urbani_HP03L_AY278741        DAFSLDVSEKSGNFKHLREFVFKNKDGFLYVYKGYQPIDVV--RDLPSGFNTLKPIFKLP
SARS-CoV-1_HGZ8L1-A_HP03E_AY394981      DAFSLDVSEKSGNFKHLREFVFKNKDGFLYVYKGYQPIDVV--RDLPSGFNTLKPIFKLP
SARS-CoV-1_GZ-C_HP03L_AY394979          DAFSLDVSEKSGNFKHLREFVFKNKDGFLYVYKGYQPIDVV--RDLPSGFNTLKPIFKLP
SARS-CoV-1_Sino1-11_HP03L_AY485277      DAFSLDVSEKSGNFKHLREFVFKNKDGFLYVYKGYQPIDVV--RDLPSGFNTLKPIFKLP
SARS-CoV-1_Sin852_HP03L_AY559082        DAFSLDVSEKSGNFKHLREFVFKNKDGFLYVYKGYQPIDVV--RDLPSGFNTLKPIFKLP
SARS-CoV-1_SZ1_PC03_AY304489            DAFSLDVSEKSGNFKHLREFVFKNKDGFLYVYKGYQPIDVV--RDLPSGFNTLKPIFKLP
SARS-CoV-1_GD03T0013_HP04_AY525636      DAFSLDVSEKSGNFKHLREFVFKNKDGFLYVYKGYQPIDVV--RDLPSGFNTLKPIFKLP
SARS-CoV-1_GZ0402_HP04_AY613947         DAFSLDVSEKSGNFKHLREFVFKNKDGFLYVYKGYQPIDVV--RDLPSGFNTLKPIFKLP
SARS-CoV-1_PC4-127_PC04_AY613951        DAFSLDVSEKSGNFKHLREFVFKNKDGFLYVYKGYQPIDVV--RDLPSGFNTLKPIFKLP
SARS-CoV-1_PC4-137_PC04_AY627045        DAFSLDVSEKSGNFKHLREFVFKNKDGFLYVYKGYQPIDVV--RDLPSGFNTLKPIFKLP
SARS-CoV-1_PC4-13_PC04_AY613948         DAFSLDVSEKSGNFKHLREFVFKNKDGFLYVYKGYQPIDVV--RDLPSGFNTLKPIFKLP
WIV1_KF367457                           KDFNLDLGEKPGNFKDLREFVFRNKDGFLHVYSGYQPISAA--SGLPTGFNALKPIFKLP
Rs7327_KY417151                         KDFNLDLGEKPGNFKDLREFVFRNKDGFLHVYSGYQPISAA--SGLPTGFNALKPIFKLP
Rs4231_KY417146                         DSFSLDVAEKSGNFKHLREFVFKNKDGFLYVYKGYQPIDVV--RDLPSGFNILKPIFKLP
RsSHC014_KC881005                       KDFNLDLGEKPGNFKDLREFVFRNKDGFLHVYSGYQPISAA--SGLPTGFNALKPIFKLP
Rs4084_KY417144                         KDFNLDLGEKPGNFKDLREFVFRNKDGFLHVYSGYQPISAA--SGLPTGFNALKPIFKLP
BtKY72_KY352407                         HGFEIDPSPNTGSFIHLREHVFRNVDGFLYVYHNYERVDVY--DNFPQGFSVLKPIFKLP
                                        . * :*   : * *  ***.**:* **:: :*  :  :      .:* **  *:*:..:*

SARS-CoV-2Wuhan_MN908947                IGINITRFQTLLALHRSYLTPGDSSSGWTAGAAAYYVGYLQPRTFLLKYNENGTITDAVD
SARS-CoV-2delta_QUD52764.1              IGINITRFQTLLALHRSYLTPGDSSSGWTAGAAAYYVGYLQPRTFLLKYNENGTITDAVD
SARS-CoV-2_BA1_UFO69279.1               IGINITRFQTLLALHRSYLTPGDSSSGWTAGAAAYYVGYLQPRTFLLKYNENGTITDAVD
SARS-CoV-2_BA2_UJP23605.1               IGINITRFQTLLALHRSYLTPGDSSSGWTAGAAAYYVGYLQPRTFLLKYNENGTITDAVD
SARS-CoV-2_BA5_UOZ45804.1               IGINITRFQTLLALHRSYLTPGDSSSGWTAGAAAYYVGYLQPRTFLLKYNENGTITDAVD
SARS-CoV-2_BQ1.1_UWM38596.1             IGINITRFQTLLALHRSYLTPGDSSSGWTAGAAAYYVGYLQPRTFLLKYNENGTITDAVD
SARS-CoV-2_EG.5.1_WGM84363.1            IGINITRFQTLLALHRSYLTPVDSSSGWTAGAAAYYVGYLQPRTFLLKYNENGTITDAVD
SARS-CoV-2_XBB.1.5_UZG29433.1           IGINITRFQTLLALHRSYLTPVDSSSGWTAGAAAYYVGYLQPRTFLLKYNENGTITDAVD
SARS-CoV-2_JN.1_WPF38074.1              IGINITRFQTLLALNRSYLTPGDSSSGWTAGAADYYVGYLQPRTFLLKYNENGTITDAVD
SARS-CoV-2_KP.3.1.1                     IGINITRFQTLLALNRSYLTPGDSSSGWTAGAADYYVGYLQPRTFLLKYNENGTITDAVD
Pangolin_GD-consensus_Lam2020           AGINITKFRTLLTIHRGDPMP---NNGWTVFSAAYYVGYLAPRTFMLNYNENGTITDAVD
RaTG13_MN996532                         IGINITRFQTLLALHRSYLTPGDSSSGWTAGAAAYYVGYLQPRTFLLKYNENGTITDAVD
Pangolin_GX-P2V_EPI_ISL_410542          IGINITRFQTLLALHRSYLTPGKLESGWTTGAAAYYVGYLQQRTFLLSYNQNGTITDAVD
SARS-CoV-1_Urbani_HP03L_AY278741        LGINITNFRAILTAFSP------AQDIWGTSAAAYFVGYLKPTTFMLKYDENGTITDAVD
SARS-CoV-1_HGZ8L1-A_HP03E_AY394981      LGINITNFRAILTAFLP------AQDTWGTSAAAYFVGYLKPTTFMLKYDENGTITDAVD
SARS-CoV-1_GZ-C_HP03L_AY394979          LGINITNFRAILTAFSP------AQDIWGTSAAAYFVGYLKPTTFMLKYDENGTITDAVD
SARS-CoV-1_Sino1-11_HP03L_AY485277      LGINITNFRAILTAFSP------AQDIWGTSAAAYFVGYLKPTTFMLKYDENGTITDAVD
SARS-CoV-1_Sin852_HP03L_AY559082        LGINITNFRAILTAFSP------AQDIWGTSAAAYFVGYLKPTTFMLKYDENGTITDAVD
```

```
SARS-CoV-1_SZ1_PC03_AY304489          LGIKITNFRAILTAFLP------AQDTWGTSAAAYFVGYLKPTKFMLKYDENGTITDAVD
SARS-CoV-1_GD03T0013_HP04_AY525636    LGINITNFRAILTAFSP------AQDTWGTSAAAYFVGYLKPTTFMLKYDENGTITDAVD
SARS-CoV-1_GZ0402_HP04_AY613947       LGINITNFRAILTAFSP------AQDTWGTSAAAYFVGYLKPTTFMLKYDENGTITDAVD
SARS-CoV-1_PC4-127_PC04_AY613951      LGINITNFRAILTAFSP------AQDTWGTSAAAYFVGYLKPTTFMLKYDENGTITDAVD
SARS-CoV-1_PC4-137_PC04_AY627045      LGINITNFRAILTAFSP------AQDTWGTSAAAYFVGYLKPTTFMLKYDENGTITDAVD
SARS-CoV-1_PC4-13_PC04_AY613948       LGINITNFRAILTAFSP------AQDTWGTSAAAYFVGYLKPTTFMLKYDENGTITDAVD
WIV1_KF367457                         LGINITNFRTLLTAFPP------RPDYWGTSAAAYFVGYLKPTTFMLKYDENGTITDAVD
Rs7327_KY417151                       LGINITNFRTLLTAFPP------RPDYWGTSAAAYFVGYLKPTTFMLKYDENGTITDAVD
Rs4231_KY417146                       LGINITNFRAILTAFLP------AQDTWGTSAAAYFVGYLKPATFMLKYDENGTITDAVD
RsSHC014_KC881005                     LGINITNFRTLLTAFPP------RPDYWGTSAAAYFVGYLKPTTFMLKYDENGTITDAVD
Rs4084_KY417144                       LGINITNFRTLLTAFPP------RPDYWGTSAAAYFVGYLKPTTFMLKYDENGTITDAVD
BtKY72_KY352407                       FGLNITQFKVIMTLFSP------TTSSFNADASVYFVGHLKPLTMLAEFDENGTITDAVD
                                       *::**.*:.:::            . : . :: *:**:*   .:: .:::*********

SARS-CoV-2Wuhan_MN908947              CALDPLSETKCTLKSFTVEKGIYQTSNFRVQPTESIVRFPNITNLCPFGEVFNATRFASV
SARS-CoV-2delta_QUD52764.1            CALDPLSETKCTLKSFTVEKGIYQTSNFRVQPTESIVRFPNITNLCPFGEVFNATRFASV
SARS-CoV-2_BA1_UFO69279.1             CALDPLSETKCTLKSFTVEKGIYQTSNFRVQPTESIVRFPNITNLCPFDEVFNATRFASV
SARS-CoV-2_BA2_UJP23605.1             CALDPLSETKCTLKSFTVEKGIYQTSNFRVQPTESIVRFPNITNLCPFDEVFNATRFASV
SARS-CoV-2_BA5_UOZ45804.1             CALDPLSETKCTLKSFTVEKGIYQTSNFRVQPTESIVRFPNITNLCPFDEVFNATRFASV
SARS-CoV-2_BQ1.1_UWM38596.1           CALDPLSETKCTLKSFTVEKGIYQTSNFRVQPTESIVRFPNITNLCPFDEVFNATTFASV
SARS-CoV-2_EG.5.1_WGM84363.1          CALDPLSETKCTLKSFTVEKGIYQTSNFRVQPTESIVRFPNITNLCPFHEVFNATTFASV
SARS-CoV-2_XBB.1.5_UZG29433.1         CALDPLSETKCTLKSFTVEKGIYQTSNFRVQPTESIVRFPNITNLCPFHEVFNATTFASV
SARS-CoV-2_JN.1_WPF38074.1            CALDPLSETKCTLKSFTVEKGIYQTSNFRVQPTESIVRFPNVTNLCPFHEVFNATRFASV
SARS-CoV-2_KP.3.1.1                   CALDPLSETKCTLKSFTVEKGIYQTSNFRVQPTESIVRFPNVTNLCPFHEVFNATRFASV
Pangolin_GD-consensus_Lam2020         CALDPLSEAKCTLKSLTVEKGIYQTSNFRVQPTESIVRFPNITNLCPFGEVFNATTFASV
RaTG13_MN996532                       CALDPLSETKCTLKSFTVEKGIYQTSNFRVQPTDSIVRFPNITNLCPFGEVFNATTFASV
Pangolin_GX-P2V_EPI_ISL_410542        CSLDPLSETKCTLKSLTVEKGIYQTSNFRVQPTISIVRFPNITNLCPFGEVFNASKFASV
SARS-CoV-1_Urbani_HP03L_AY278741      CSQNPLAELKCSVKSFEIDKGIYQTSNFRVVPSGDVVRFPNITNLCPFGEVFNATKFPSV
SARS-CoV-1_HGZ8L1-A_HP03E_AY394981    CSQNPLAELKCSVKSFEIDKGIYQTSNFRVVPSRDVVRFPNITNLCPFGEVFNATKFPSV
SARS-CoV-1_GZ-C_HP03L_AY394979        CSQNPLAELKCSVKSFEIDKGIYQTSNFRVVPSGDVVRFPNITNLCPFGEVFNATKFPSV
SARS-CoV-1_Sino1-11_HP03L_AY485277    CSQNPLAELKCSVKSFEIDKGIYQTSNFRVVPSGDVVRFPNITNLCPFGEVFNATKFPSV
SARS-CoV-1_Sin852_HP03L_AY559082      CSQNPLAELKCSVKSFEIDKGIYQTSNFRVVPSGDVVRFPNITNLCPFGEVFNATKFPSV
SARS-CoV-1_SZ1_PC03_AY304489          CSQNPLAELKCSVKSFEIDKGIYQTSNFRVVPSGDVVRFPNITNLCPFGEVFNATKFPSV
SARS-CoV-1_GD03T0013_HP04_AY525636    CSQNPLAELKCSVKSFEIDKGIYQTSNFRVVPSGDVVRFPNITNLCPFGEVFNATKFPSV
SARS-CoV-1_GZ0402_HP04_AY613947       CSQNPLAELKCSVKSFEIDKGIYQTSNFRVVPSGDVVRFPNITNLCPFGEVFNATKFPSV
SARS-CoV-1_PC4-127_PC04_AY613951      CSQNPLAELKCSVKSFEIDKGIYQTSNFRVVPSGDVVRFPNITNLCPFGEVFNATKFPSV
SARS-CoV-1_PC4-137_PC04_AY627045      CSQNPLAELKCSVKSFEIDKGIYQTSNFRVVPSGDVVRFPNITNLCPFGEVFNATKFPSV
SARS-CoV-1_PC4-13_PC04_AY613948       CSQNPLAELKCSVKSFEIDKGIYQTSNFRVVPSGDVVRFPNITNLCPFGEVFNATKFPPV
WIV1_KF367457                         CSQNPLAELKCSVKSFEIDKGIYQTSNFRVAPSKEVVRFPNITNLCPFGEVFNATTFPSV
Rs7327_KY417151                       CSQNPLAELKCSVKSFEIDKGIYQTSNFRVAPSKEVVRFPNITNLCPFGEVFNATTFPSV
Rs4231_KY417146                       CSQNPLAELKCSVKSFEIDKGIYQTSNFRVAPSKEVVRFPNITNLCPFGEVFNATTFPSV
RsSHC014_KC881005                     CSQNPLAELKCSVKSFEIDKGIYQTSNFRVAPSKEVVRFPNITNLCPFGEVFNATTFPSV
Rs4084_KY417144                       CSQNPLAELKCSVKSFEIDKGIYQTSNFRVAPSKEVVRFPNITNLCPFGEVFNATTFPSV
BtKY72_KY352407                       CSQDPLSELKCTTKSLTVEKGIYQTSNFRVSPSTEVVRFPNITNLCPFGQVFNASNFPSV
                                      *: :**:* **: **: ::*********** *: .:*****:****** :****: *  *

SARS-CoV-2Wuhan_MN908947              YAWNRKRISNCVADYSVLYNS-ASFSTFKCYGVSPTKLNDLCFTNVYADSFVIRGDEVRQ
SARS-CoV-2delta_QUD52764.1            YAWNRKRISNCVADYSVLYNS-ASFSTFKCYGVSPTKLNDLCFTNVYADSFVIRGDEVRQ
SARS-CoV-2_BA1_UFO69279.1             YAWNRKRISNCVADYSVLYNL-APFFTFKCYGVSPTKLNDLCFTNVYADSFVIRGDEVRQ
SARS-CoV-2_BA2_UJP23605.1             YAWNRKRISNCVADYSVLYNF-APFFAFKCYGVSPTKLNDLCFTNVYADSFVIRGNEVSQ
SARS-CoV-2_BA5_UOZ45804.1             YAWNRKRISNCVADYSVLYNF-APFFAFKCYGVSPTKLNDLCFTNVYADSFVIRGNEVSQ
SARS-CoV-2_BQ1.1_UWM38596.1           YAWNRKRISNCVADYSVLYNF-APFFAFKCYGVSPTKLNDLCFTNVYADSFVIRGNEVSQ
SARS-CoV-2_EG.5.1_WGM84363.1          YAWNRKRISNCVADYSVIYNF-APFFAFKCYGVSPTKLNDLCFTNVYADSFVIRGNEVSQ
SARS-CoV-2_XBB.1.5_UZG29433.1         YAWNRKRISNCVADYSVIYNF-APFFAFKCYGVSPTKLNDLCFTNVYADSFVIRGNEVSQ
SARS-CoV-2_JN.1_WPF38074.1            YAWNRTRISNCVADYSVLYNF-APFFAFKCYGVSPTKLNDLCFTNVYADSFVIKGNEVSQ
SARS-CoV-2_KP.3.1.1                   YAWNRTRISNCVADYSVLYNF-APFFAFKCYGVSPTKLNDLCFTNVYADSFVIKGNEVSQ
Pangolin_GD-consensus_Lam2020         YAWNRKRISNCVADYSVLYNS-TSFSTFKCYGVSPTKLNDLCFTNVYADSFVVRGDEVRQ
RaTG13_MN996532                       YAWNRKRISNCVADYSVLYNS-TSFSTFKCYGVSPTKLNDLCFTNVYADSFVITGDEVRQ
Pangolin_GX-P2V_EPI_ISL_410542        YAWNRKRISNCVADYSVLYNS-TSFSTFKCYGVSPTKLNDLCFTNVYADSFVVKGDEVRQ
SARS-CoV-1_Urbani_HP03L_AY278741      YAWERKKISNCVADYSVLYNS-TFFSTFKCYGVSATKLNDLCFSNVYADSFVVKGDDVRQ
SARS-CoV-1_HGZ8L1-A_HP03E_AY394981    YAWERKRISNCVADYSVLYNS-TFFSTFKCYGVSATKLNDLCFSNVYADSFVVKGDDVRQ
SARS-CoV-1_GZ-C_HP03L_AY394979        YAWERKKISNCVADYSVLYNS-TFFSTFKCYGVSATKLNDLCFSNVYADSFVVKGDDVRQ
SARS-CoV-1_Sino1-11_HP03L_AY485277    YAWERKKISNCVADYSVLYNS-TFFSTFKCYGVSATKLNDLCFSNVYADSFVVKGDDVRQ
SARS-CoV-1_Sin852_HP03L_AY559082      YAWERKKISNCVADYFVLYNS-TFFSTFKCYGVSATKLNDLCFSNVYADSFVVKGDDVRQ
SARS-CoV-1_SZ1_PC03_AY304489          YAWERKRISNCVADYSVLYNS-TSFSTFKCYGVSATKLNDLCFSNVYADSFVVKGDDVRQ
SARS-CoV-1_GD03T0013_HP04_AY525636    YAWERKRISNCVADYSVLYNS-TSFSTFKCYGVSATKLNDLCFSNVYADSFVVKGDDVRQ
SARS-CoV-1_GZ0402_HP04_AY613947       YAWERKRISNCVADYSVLYNS-TSFSTFKCYGVSATKLNDLCFSNVYADSFVVKGDDVRQ
SARS-CoV-1_PC4-127_PC04_AY613951      YAWERKRISNCVADYSVLYNS-TSFSTFKCYGVSATKLNDLCFSNVYADSFVVKGDDVRQ
SARS-CoV-1_PC4-137_PC04_AY627045      YAWERKRISNCVADYSVLYNS-TSFSTFKCYGVSATKLNDLCFSNVYADSFVVKGDDVRQ
SARS-CoV-1_PC4-13_PC04_AY613948       YAWERKRISNCVADYSVLYNS-TSFSTFKCYGVSATKLNDLCFSNVYADSFVVKGDDVRQ
WIV1_KF367457                         YAWERKRISNCVADYSVLYNS-TSFSTFKCYGVSATKLNDLCFSNVYADSFVVKGDDVRQ
Rs7327_KY417151                       YAWERKRISNCVADYSVLYNS-TSFSTFKCYGVSATKLNDLCFSNVYADSFVVKGDDVRQ
Rs4231_KY417146                       YAWERKRISNCVADYSVLYNS-TSFSTFKCYGVSATKLNDLCFSNVYADSFVVKGDDVRQ
RsSHC014_KC881005                     YAWERKRISNCVADYSVLYNS-TSFSTFKCYGVSATKLNDLCFSNVYADSFVVKGDDVRQ
Rs4084_KY417144                       YAWERKRISNCVADYSILYNS-TSFSTFKCYGVSATKLNDLCFSNVYADSFVVKGDDVRQ
BtKY72_KY352407                       YAWERLRISDCVADYAVLYNSSSSFSTFKCYGVSPTKLNDLCFSSVYADYFVVKGDDVRQ
```

```
                                          ***:* :**:***** ::**  : * :******* ********:.**** **: *::* *

SARS-CoV-2Wuhan_MN908947                  IAPGQTGKIADYNYKLPDDFTGCVIAWNSNNLDSKVGGNYNYLYRLFRKSNLKPFERDIS
SARS-CoV-2delta_QUD52764.1                IAPGQTGKIADYNYKLPDDFTGCVIAWNSNNLDSKVGGNYNYRYRLFRKSNLKPFERDIS
SARS-CoV-2_BA1_UFO69279.1                 IAPGQTGNIADYNYKLPDDFTGCVIAWNSNKLDSKVSGNYNYLYRLFRKSNLKPFERDIS
SARS-CoV-2_BA2_UJP23605.1                 IAPGQTGNIADYNYKLPDDFTGCVIAWNSNKLDSKVGGNYNYLYRLFRKSNLKPFERDIS
SARS-CoV-2_BA5_UOZ45804.1                 IAPGQTGNIADYNYKLPDDFTGCVIAWNSNKLDSKVGGNYNYRYRLFRKSNLKPFERDIS
SARS-CoV-2_BQ1.1_UWM38596.1               IAPGQTGNIADYNYKLPDDFTGCVIAWNSNKLDSTVGGNYNYRYRLFRKSKLKPFERDIS
SARS-CoV-2_EG.5.1_WGM84363.1              IAPGQTGNIADYNYKLPDDFTGCVIAWNSNKLDSKPSGNYNYLYRLLRKSKLKPFERDIS
SARS-CoV-2_XBB.1.5_UZG29433.1             IAPGQTGNIADYNYKLPDDFTGCVIAWNSNKLDSKPSGNYNYLYRLFRKSKLKPFERDIS
SARS-CoV-2_JN.1_WPF38074.1                IAPGQTGNIADYNYKLPDDFTGCVIAWNSNKLDSKHSGNYDYWYRSFRKSKLKPFERDIS
SARS-CoV-2_KP.3.1.1                       IAPGQTGNIADYNYKLPDDFTGCVIAWNSNKLDSKHSGNYDYWYRSLRKSKLKPFERDIS
Pangolin_GD-consensus_Lam2020             IAPGQTGRIADYNYKLPDDFTGCVIAWNSNNLDSKVGGNYNYLYRLFRKSNLKPFERDIS
RaTG13_MN996532                           IAPGQTGKIADYNYKLPDDFTGCVIAWNSKHIDAKEGGNFNYLYRLFRKANLKPFERDIS
Pangolin_GX-P2V_EPI_ISL_410542            IAPGQTGVIADYNYKLPDDFTGCVIAWNSVKQDALTGGNYGYLYRLFRKSKLKPFERDIS
SARS-CoV-1_Urbani_HP03L_AY278741          IAPGQTGVIADYNYKLPDDFMGCVLAWNTRNIDATSTGNYNYKYRYLRHGKLRPFERDIS
SARS-CoV-1_HGZ8L1-A_HP03E_AY394981        IAPGQTGVIADYNYKLPDDFMGCVLAWNTRNIDATSTGNYNYKYRYLRHGKLRPFERDIS
SARS-CoV-1_GZ-C_HP03L_AY394979            IAPGQTGVIADYNYKLPDDFMGCVLAWNTRNIDATSTGNHNYKYRYLRHGKLRPFERDIS
SARS-CoV-1_Sino1-11_HP03L_AY485277        IAPGQTGVIADYNYKLPDDFMGCVLAWNTRNIDATSTGNYNYKYRSLRHGKLRPFERDIS
SARS-CoV-1_Sin852_HP03L_AY559082          IAPGQTGVIADYNYKLPDDFMGCVLAWNTRNIDATSTGNYNYKYRYLRHGKLRPFERDIS
SARS-CoV-1_SZ1_PC03_AY304489              IAPGQTGVIADYNYKLPDDFMGCVLAWNTRNIDATSTGNYNYKYRYLRHGKLRPFERDIS
SARS-CoV-1_GD03T0013_HP04_AY525636        IAPGQTGVIADYNYKLPDDFMGCVLAWNTRNIDATSTGNYNYKYRYLRHGKLRPFERDIS
SARS-CoV-1_GZ0402_HP04_AY613947           IAPGQTGVIADYNYKLPDDFMGCVLAWNIRNIDATSTGNYNYKYRYLRHGKLRPFERDIS
SARS-CoV-1_PC4-127_PC04_AY613951          IAPGQTGVIADYNYKLPDDFMGCVLAWNTRNIDATSTGNYNYKYRYLRHGKLRPFERDIS
SARS-CoV-1_PC4-137_PC04_AY627045          IAPGQTGVIADYNYKLPDDFMGCVLAWNTRNIDATSTGNYNYKYRYLRHGKLRPFERDIS
SARS-CoV-1_PC4-13_PC04_AY613948           IAPGQTGVIADYNYKLPDDFMGCVLAWNTRNIDATSTGNYNYKYRYLRHGKLRPFERDIS
WIV1_KF367457                             IAPGQTGVIADYNYKLPDDFTGCVLAWNTRNIDATQTGNYNYKYRSLRHGKLRPFERDIS
Rs7327_KY417151                           IAPGQTGVIADYNYKLPDDFMGCVLAWNTRNIDATSTGNYNYKYRSLRHGKLRPFERDIS
Rs4231_KY417146                           IAPGQTGVIADYNYKLPDDFLGCVLAWNTNSKDSSTSGNYNYLYRWVRRSKLNPYERDLS
RsSHC014_KC881005                         IAPGQTGVIADYNYKLPDDFLGCVLAWNTNSKDSSTSGNYNYLYRWVRRSKLNPYERDLS
Rs4084_KY417144                           IAPGQTGVIADYNYKLPDDFLGCVLAWNTNSKDSSTSGNYNYLYRWVRRSKLNPYERDLS
BtKY72_KY352407                           IAPAQTGVIADYNYKLPDDFTGCVLAWNTNSVDSKSGNN--FYYRLFRHGKIKPYERDIS
                                          ***.*** ************ ***:***    *:   .*  : ** .*:.::.*:***:*

SARS-CoV-2Wuhan_MN908947                  TEIYQAGSTPCNGVEGFNCYFPLQSYGFQPTNGVGYQPYRVVVLSFELLHAPATVCGPKK
SARS-CoV-2delta_QUD52764.1                TEIYQAGSKPCNGVEGFNCYFPLQSYGFQPTNGVGYQPYRVVVLSFELLHAPATVCGPKK
SARS-CoV-2_BA1_UFO69279.1                 TEIYQAGNKPCNGVAGFNCYFPLRSYSFRPTYGVGHQPYRVVVLSFELLHAPATVCGPKK
SARS-CoV-2_BA2_UJP23605.1                 TEIYQAGNKPCNGVAGFNCYFPLRSYGFRPTYGVGHQPYRVVVLSFELLHAPATVCGPKK
SARS-CoV-2_BA5_UOZ45804.1                 TEIYQAGNKPCNGVAGVNCYFPLQSYGFRPTYGVGHQPYRVVVLSFELLHAPATVCGPKK
SARS-CoV-2_BQ1.1_UWM38596.1               TEIYQAGNKPCNGVAGVNCYFPLQSYGFRPTYGVGHQPYRVVVLSFELLHAPATVCGPKK
SARS-CoV-2_EG.5.1_WGM84363.1              TEIYQAGNKPCNGVAGPNCYSPLQSYGFRPTYGVGHQPYRVVVLSFELLHAPATVCGPKK
SARS-CoV-2_XBB.1.5_UZG29433.1             TEIYQAGNKPCNGVAGPNCYSPLQSYGFRPTYGVGHQPYRVVVLSFELLHAPATVCGPKK
SARS-CoV-2_JN.1_WPF38074.1                TEIYQAGNKPCKG-KGPNCYFPLQSYGFRPTYGVGHQPYRVVVLSFELLHAPATVCGPKK
SARS-CoV-2_KP.3.1.1                       TEIYQAGNKPCKG-KGPNCYFPLESYGFRPTYGVGHQPYRVVVLSFELLHAPATVCGPKK
Pangolin_GD-consensus_Lam2020             TEIYQAGSTPCNGVEGFNCYFPLQSYGFHPTNGVGYQPYRVVVLSFELLNAPATVCGPKQ
RaTG13_MN996532                           TEIYQAGSKPCNGQTGLNCYYPLYRYGFYPTDGVGHQPYRVVVLSFELLNAPATVCGPKK
Pangolin_GX-P2V_EPI_ISL_410542            TEIYQAGSTPCNGQVGLNCYYPLERYGFHPTTGVNYQPFRVVVLSFELLNGPATVCGPKL
SARS-CoV-1_Urbani_HP03L_AY278741          NVPFSPDGKPCTP-PALNCYWPLNDYGFYTTTGIGYQPYRVVVLSFELLNAPATVCGPKL
SARS-CoV-1_HGZ8L1-A_HP03E_AY394981        NVPFSPDGKPCTP-PALNCYWPLNDYGFYTTTGIGYQPYRVVVLSFELLNAPATVCGPKL
SARS-CoV-1_GZ-C_HP03L_AY394979            NVPFSPDGKPCTP-PALNCYWPLNDYGFYTTTGIGYQPYRVVVLSFELLNAPATVCGPKL
SARS-CoV-1_Sino1-11_HP03L_AY485277        NVPFSPDGKPCTP-PALNCYWPLNDYGFYTTTGIGYQPYRVVVLSFELLNAPATVCGPKL
SARS-CoV-1_Sin852_HP03L_AY559082          NVPFSPDGKPCTP-PALNCYWPLNDYGFYTTTGIGYQPYRVVVLSFELLNAPATVCGPKL
SARS-CoV-1_SZ1_PC03_AY304489              NVPFSPDGKPCTP-PALNCYWPLKDYGFYTTSGIGYQPYRVVVLSFELLNAPATVCGPKL
SARS-CoV-1_GD03T0013_HP04_AY525636        NVPFSPDGKPCTP-PAPNCYWPLNGYGFYTTSGIGYQPYRVVVLSFELLNAPATVCGPKL
SARS-CoV-1_GZ0402_HP04_AY613947           NVPFSPDGKPCTP-PAPNCYWPLNGYGFYTTSGIGYQPYRVVVLSFELLNAPATVCGPKL
SARS-CoV-1_PC4-127_PC04_AY613951          NVPFSPDGKPCTP-PAPNCYWPLRGYGFYTTSGIGYQPYRVVVLSFELLNAPATVCGPKL
SARS-CoV-1_PC4-137_PC04_AY627045          NVPFSPDGKPCTP-PAPNCYWPLKGYGFYTTSGIGYQPYRVVVLSFELLNAPATVCGPKL
SARS-CoV-1_PC4-13_PC04_AY613948           NVPFSSDGKPCTP-PAPNCYWPLRGYGFYTTSGIGYQPYRVVVLSFELLNAPATVCGPKL
WIV1_KF367457                             NVPFSPDGKPCTP-PAFNCYWPLNDYGFYITNGIGYQPYRVVVLSFELLNAPATVCGPKL
Rs7327_KY417151                           NVPFSPDGKPCTP-PAFNCYWPLNDYGFFTTNGIGYQPYRVVVLSFELLNAPATVCGPKL
Rs4231_KY417146                           NDIYSPGGQSCSA-IGPNCYNPLRPYGFFTTAGVGHQPYRVVVLSFELLNAPATVCGPKL
RsSHC014_KC881005                         NDIYSPGGQSCSA-VGPNCYNPLRPYGFFTTAGVGHQPYRVVVLSFELLNAPATVCGPKL
Rs4084_KY417144                           NDIYSPGGQSCSA-VGPNCYNPLRPYGFFTTAGVGHQPYRVVVLSFELLNAPATVCGPKL
BtKY72_KY352407                           NVLYNSAGGTCSSISQLGCYEPLKSYGFTPTVGVGYQPYRVVVLSFELLNAPATVCGPKK
                                          .  :.  .  *.     .** **  *.*  * *:.:**:**********:.********

SARS-CoV-2Wuhan_MN908947                  STNLVKNKCVNFNFNGLTGTGVLTESNKKFLPFQQFGRDIADTTDAVRDPQTLEILDITP
SARS-CoV-2delta_QUD52764.1                STNLVKNKCVNFNFNGLTGTGVLTESNKKFLPFQQFGRDIADTTDAVRDPQTLEILDITP
SARS-CoV-2_BA1_UFO69279.1                 STNLVKNKCVNFNFNGLKGTGVLTESNKKFLPFQQFGRDIADTTDAVRDPQTLEILDITP
SARS-CoV-2_BA2_UJP23605.1                 STNLVKNKCVNFNFNGLTGTGVLTESNKKFLPFQQFGRDIADTTDAVRDPQTLEILDITP
SARS-CoV-2_BA5_UOZ45804.1                 STNLVKNKCVNFNFNGLTGTGVLTESNKKFLPFQQFGRDIADTTDAVRDPQTLEILDITP
SARS-CoV-2_BQ1.1_UWM38596.1               STNLVKNKCVNFNFNGLTGTGVLTESNKKFLPFQQFGRDIADTTDAVRDPQTLEILDITP
SARS-CoV-2_EG.5.1_WGM84363.1              STNLVKNKCVNFNFNGLTGTGVLTESNKKFLPFQQFGRDIADTTDAVRDPQTLEILDITP
SARS-CoV-2_XBB.1.5_UZG29433.1             STNLVKNKCVNFNFNGLTGTGVLTESNKKFLPFQQFGRDIADTTDAVRDPQTLEILDITP
SARS-CoV-2_JN.1_WPF38074.1                STNLVKNKCVNFNFNGLTGTGVLTKSNKKFLPFQQFGRDIVDTTDAVRDPQTLEILDITP
SARS-CoV-2_KP.3.1.1                       STNLVKNKCVNFNFNGLTGTGVLTKSNKKFLPFQQFGRDIVDTTDAVRDPQTLEILDITP
```

```
Pangolin_GD-consensus_Lam2020          STNLVKNKCVNFNFNGLTGTGVLTESSKKFLPFQQFGRDIADTTDAVRDPQTLEILDITP
RaTG13_MN996532                        STNLVKNKCVNFNFNGLTGTGVLTESNKKFLPFQQFGRDIADTTDAVRDPQTLEILDITP
Pangolin_GX-P2V_EPI_ISL_410542         STTLVKDKCVNFNFNGLTGTGVLTTSKKQFLPFQQFGRDISDTTDAVRDPQTLEILDITP
SARS-CoV-1_Urbani_HP03L_AY278741       STDLIKNQCVNFNFNGLTGTGVLTPSSKRFQPFQQFGRDVSDFTDSVRDPKTSEILDISP
SARS-CoV-1_HGZ8L1-A_HP03E_AY394981     STDLIKNQCVNFNFNGLTGTGVLTPSSKRFQPFQQFGRDVSDFTDSVRDPKTSEILDISP
SARS-CoV-1_GZ-C_HP03L_AY394979         STDLIKNQCVNFNFNGLTGTGVLTPSSKRFQPFQQFGRDVSDFTDSVRDPKTSEILDISP
SARS-CoV-1_Sino1-11_HP03L_AY485277     STDLIKNQCVNFNFNGLTGTGVLTPSSKRFQPFQQFGRDVSDFTDSVRDPKTSEILDISP
SARS-CoV-1_Sin852_HP03L_AY559082       STDLIKNQCVNFNFNGLTGTGVLTPSSKRFQPFQQFGRDVSDFTDSVRDPKTSEILDISP
SARS-CoV-1_SZ1_PC03_AY304489           STDLIKNQCVNFNFNGLTGTGVLTPSSKRFQPFQQFGRDVSDITDSVRDPKTSEILDISP
SARS-CoV-1_GD03T0013_HP04_AY525636     STDLIKNQCVNFNFNGLTGTGVLTPSSKRFQPFQQFGRDVSDFTDSVRDPKTSEILDISP
SARS-CoV-1_GZ0402_HP04_AY613947        STDLIKNQCVNFNFNGLTGTGVLTPSSKRFQPFQQFGRDVSDFTDSVRDPKTSEILDISP
SARS-CoV-1_PC4-127_PC04_AY613951       STDLIKNQCVNFNFNGLTGTGVLTPSSKRFQPFQQFGRDVSDFTDSVRDPKTSEILDISP
SARS-CoV-1_PC4-137_PC04_AY627045       STDLIKNQCVNFNFNGLTGTGVLTPSSKRFQPFQQFGRDVSDFTDSVRDPKTSEILDISP
SARS-CoV-1_PC4-13_PC04_AY613948        STDLIKNQCVNFNFNGLTGTGVLTPSSKRFQPFQQFGRDVSDFTDSVRDPKTSEILDISP
WIV1_KF367457                          STDLIKNQCVNFNFNGLTGTGVLTPSSKRFQPFQQFGRDVSDFTDSVRDPKTSEILDISP
Rs7327_KY417151                        STDLIKNQCVNFNFNGLTGTGVLTSSSKRFQPFQQFGRDVSDFTDSVRDPKTSEILDISP
Rs4231_KY417146                        STDLIKNQCVNFNFNGLTGTGVLTSSSKRFQPFQQFGRDVSDFTDSVRDPKTSEILDISP
RsSHC014_KC881005                      STDLIKNQCVNFNFNGLTGTGVLTPSSKRFQPFQQFGRDVSDFTDSVRDPKTSEILDISP
Rs4084_KY417144                        STDLIKNQCVNFNFNGLTGTGVLTPSSKRFQPFQQFGRDVSDFTDSVRDPKTSEILDISP
BtKY72_KY352407                        STELVKNKCVNFNFNGLTGTGVLTSSTKKFQPFQQFGRDVSDFTDSVRDPKTFEILDISP
                                       ** *:*::*********.****** *.*:* ********: * **:****:* *****:*

SARS-CoV-2Wuhan_MN908947               CSFGGVSVITPGTNTSNQVAVLYQDVNCTEVPVAIHADQLTPTWRVYSTG--SNVFQTRA
SARS-CoV-2delta_QUD52764.1             CSFGGVSVITPGTNTSNQVAVLYQGVNCTEVPVAIHADQLTPTWRVYSTG--SNVFQTRA
SARS-CoV-2_BA1_UFO69279.1              CSFGGVSVITPGTNTSNQVAVLYQGVNCTEVPVAIHADQLTPTWRVYSTG--SNVFQTRA
SARS-CoV-2_BA2_UJP23605.1              CSFGGVSVITPGTNTSNQVAVLYQGVNCTEVPVAIHADQLTPTWRVYSTG--SNVFQTRA
SARS-CoV-2_BA5_UOZ45804.1              CSFGGVSVITPGTNTSNQVAVLYQGVNCTEVPVAIHADQLTPTWRVYSTG--SNVFQTRA
SARS-CoV-2_BQ1.1_UWM38596.1            CSFGGVSVITPGTNTSNQVAVLYQGVNCTEVPVAIHADQLTPTWRVYSTG--SNVFQTRA
SARS-CoV-2_EG.5.1_WGM84363.1           CSFGGVSVITPGTNTSNQVAVLYQGVNCTEVPVAIHADQLTPTWRVYSTG--SNVFQTRA
SARS-CoV-2_XBB.1.5_UZG29433.1          CSFGGVSVITPGTNTSNQVAVLYQGVNCTEVPVAIHADQLTPTWRVYSTG--SNVFQTRA
SARS-CoV-2_JN.1_WPF38074.1             CSFGGVSVITPGTNTSNQVAVLYQGVNCTEVSVAIHADQLTPTWRVYSTG--SNVFQTRA
SARS-CoV-2_KP.3.1.1                    CSFGGVSVITPGTNTSNQVAVLYQGVNCTEVSVAIHADQLTPTWRVYSTG--SNVFQTRA
Pangolin_GD-consensus_Lam2020          CSFGGVSVITPGTNTSNQVAVLYQDVNCTEVPVAIHADQLTPTWSVYSTG--SNVFQTRA
RaTG13_MN996532                        CSFGGVSVITPGTNASNQVAVLYQDVNCTEVPVAIHADQLTPTWRVYSTG--SNVFQTRA
Pangolin_GX-P2V_EPI_ISL_410542         CSFGGVSVITPGTNTSNQVAVLYQDVNCTEVPMAIHAEQLTPAWRVYSAG--ANVFQTRA
SARS-CoV-1_Urbani_HP03L_AY278741       CSFGGVSVITPGTNASSEVAVLYQDVNCTDVSTAIHADQLTPAWRIYSTG--NNVFQTQA
SARS-CoV-1_HGZ8L1-A_HP03E_AY394981     CSFGGVSVITPGTNASSEVAVLYQDVNCTDVSTAIHADQLTPAWRIYSTG--NNVFQTQA
SARS-CoV-1_GZ-C_HP03L_AY394979         CSFGGVSVITPGTNASSEVAVLYQDVNCTDVSTAIHADQLTPAWRIYSTG--NNVFQTQA
SARS-CoV-1_Sino1-11_HP03L_AY485277     CSFGGVSVITPGTNASSEVAVLYQDVNCTDVSTAIHADQLTPAWRIYSTG--NNVFQTQA
SARS-CoV-1_Sin852_HP03L_AY559082       CSFGGVSVITPGTNASSEVAVLYQDVNCTDVSTAIHADQLTPAWRIYSTG--NNVFQTQA
SARS-CoV-1_SZ1_PC03_AY304489           CSFGGVSVITPGTNASSEVAVLYQDVNCTDVPTAIHADQLTPAWRIYSTG--NNVFQTQA
SARS-CoV-1_GD03T0013_HP04_AY525636     CSFGGVSVITPGTNASSEVAVLYQDVNCTDVSTLIHAEQLTPAWRIYSTG--NNVFQTQA
SARS-CoV-1_GZ0402_HP04_AY613947        CSFGGVSVITPGTNASSEVAVLYQDVNCTDVSIAIHADQLTPAWRIYSTG--NNVFQTQA
SARS-CoV-1_PC4-127_PC04_AY613951       CSFGGVSVITPGTNASSEVAVLYQDVNCTDVSTLIHAEQLTPAWRIYSTG--NNVFQTQA
SARS-CoV-1_PC4-137_PC04_AY627045       CSFGGVSVITPGTNASSEVAVLYQDVNCTDVSTLIHADQLTPAWRIYSTG--NNVFQTQA
SARS-CoV-1_PC4-13_PC04_AY613948        CSFGGVSVITPGTNASSEVAVLYQDVNCTDVSTLIHAEQLTPAWRIYSTG--NNVFQTQA
WIV1_KF367457                          CSFGGVSVITPGTNTSSEVAVLYQDVNCTDVPVAIHADQLTPSWRVHSTG--NNVFQTQA
Rs7327_KY417151                        CSFGGVSVITPGTNTSSEVAVLYQDVNCTDVPVAIHADQLTPAWRIYSTG--NNVFQTQA
Rs4231_KY417146                        CSFGGVSVITPGTNTSSEVAVLYQDVNCTDVPVAIHADQLTPAWRIYSTG--NNVFQTQA
RsSHC014_KC881005                      CSFGGVSVITPGTNTSSEVAVLYQDVNCTDVPVAIHADQLTPSWRVYSTG--NNVFQTQA
Rs4084_KY417144                        CSFGGVSVITPGTNTSSEVAVLYQDVNCTDVPVAIHADQLTPSWRVYSTG--NNVFQTQA
BtKY72_KY352407                        CSYGGVSVITPGTNTSKAVAVLYQDVNCTDVPTMIHVEQVSSDWRVYAFNSYGNMFQTQA
                                       **:***********:*. ******.****:*   **.:*::  * ::: .   *:***:*

SARS-CoV-2Wuhan_MN908947               GCLIGAEHVNNSYECDIPIGAGICASYQTQTNSPRRARSVASQSIIAYTMSLGAENSVAY
SARS-CoV-2delta_QUD52764.1             GCLIGAEHVNNSYECDIPIGAGICASYQTQTNSRRRARSVASQSIIAYTMSLGAENSVAY
SARS-CoV-2_BA1_UFO69279.1              GCLIGAEYVNNSYECDIPIGAGICASYQTQTKSHRRARSVASQSIIAYTMSLGAENSVAY
SARS-CoV-2_BA2_UJP23605.1              GCLIGAEYVNNSYECDIPIGAGICASYQTQTKSHRRARSVASQSIIAYTMSLGAENSVAY
SARS-CoV-2_BA5_UOZ45804.1              GCLIGAEYVNNSYECDIPIGAGICASYQTQTKSHRRARSVASQSIIAYTMSLGAENSVAY
SARS-CoV-2_BQ1.1_UWM38596.1            GCLIGAEYVNNSYECDIPIGAGICASYQTQTKSHRRARSVASQSIIAYTMSLGAENSVAY
SARS-CoV-2_EG.5.1_WGM84363.1           GCLIGAEYVNNSYECDIPIGAGICASYQTQTKSHRRARSVASQSIIAYTMSLGAENSVAY
SARS-CoV-2_XBB.1.5_UZG29433.1          GCLIGAEYVNNSYECDIPIGAGICASYQTQTKSHRRARSVASQSIIAYTMSLGAENSVAY
SARS-CoV-2_JN.1_WPF38074.1             GCLIGAEYVNNSYECDIPIGAGICASYQTQTKSRRRARSVASQSIIAYTMSLGAENSVAY
SARS-CoV-2_KP.3.1.1                    GCLIGAEYVNNSYECDIPIGAGICASYQTQTKSRRRARSVASQSIIAYTMSLGAENSVAY
Pangolin_GD-consensus_Lam2020          GCLIGAEHVNNSYECDIPIGAGICASYQTQTN----SRSVSSQAIIAYTMSLGAENSVAY
RaTG13_MN996532                        GCLIGAEHVNNSYECDIPIGAGICASYQTQTNS----RSVASQSIIAYTMSLGAENSVAY
Pangolin_GX-P2V_EPI_ISL_410542         GCLVGAEHVNNSYECDIPVGAGICASYHSMSS----FRSVNQRSIIAYTMSLGAENSVAY
SARS-CoV-1_Urbani_HP03L_AY278741       GCLIGAEHVDTSYECDIPIGAGICASYHTVSL----LRSTSQKSIVAYTMSLGADSSIAY
SARS-CoV-1_HGZ8L1-A_HP03E_AY394981     GCLIGAEHVDTSYECDIPIGAGICASYHTVSL----LRSTSQKSIVAYTMSLGADSSIAY
SARS-CoV-1_GZ-C_HP03L_AY394979         GCLIGAEHVDTSYECDIPIGAGICASYHTVSL----LRSTSQKSIVAYTMSLGADSSIAY
SARS-CoV-1_Sino1-11_HP03L_AY485277     GCLIGAEHVDTSYECDIPIGAGICASYHTVSL----LRSTSQKSIVAYTMSLGADSSIAY
SARS-CoV-1_Sin852_HP03L_AY559082       GCLIGAEHVDTSYECDIPIGAGICASYHTVSL----LRSTSQKSIVAYTMSLGADSSIAY
SARS-CoV-1_SZ1_PC03_AY304489           GCLIGAEHVDTSYECDIPIGAGICASYHTVSS----LRSTSQKSIVAYTMSLGADSSIAY
SARS-CoV-1_GD03T0013_HP04_AY525636     GCLIGAEHVDTSYECDIPIGAGICASYHTVSS----LRSTSQKSIVAYTMSLGADSSIAY
SARS-CoV-1_GZ0402_HP04_AY613947        GCLIGAEHVDTSYECDIPIGAGICASYHTVSS----LRSTSQKSIVAYTMSLGADSSIAY
SARS-CoV-1_PC4-127_PC04_AY613951       GCLIGAEHVDTSYECDIPIGAGICASYHTVSS----LRSTSQKSIVAYTMSLGADSSIAY
```

```
SARS-CoV-1_PC4-137_PC04_AY627045        GCLIGAEHVDTSYECDIPIGAGICASYHTVSS----LRSTSQKSIVAYTMSLGADSSIAY
SARS-CoV-1_PC4-13_PC04_AY613948         GCLIGAEHVDTSYECDIPIGAGICASYHTVSS----LRSTSQKSIVAYTMSLGADSSIAY
WIV1_KF367457                           GCLIGAEHVDTSYECDIPIGAGICASYHTVSS----LRSTSQKSIVAYTMSLGADSSIAY
Rs7327_KY417151                         GCLIGAEHVDTSYECDIPIGAGICASYHTVSS----LRSTSQKSIVAYTMSLGADSSIVY
Rs4231_KY417146                         GCLIGAEHVDTSYECDIPIGAGICASYHTVSS----LRSTSQKSIVAYTMSLGADSSIAY
RsSHC014_KC881005                       GCLIGAEHVDTSYECDIPIGAGICASYHTVSS----LRSTSQKSIVAYTMSLGADSSIAY
Rs4084_KY417144                         GCLIGAEHVDTSYECDIPIGAGICASYHTVSS----LRSTSQKSIVAYTMSLGADSSIAY
BtKY72_KY352407                         GCLVGAIYENTTYECDIPIGAGICAKFGSDKI------RMGQESIVAYTMSIGEDQSIAY
                                        ***:** : :.:******:******.: : .          ..:*:*****:* :.*:.*

SARS-CoV-2Wuhan_MN908947                SNNSIAIPTNFTISVTTEILPVSMTKTSVDCTMYICGDSTECSNLLLQYGSFCTQLNRAL
SARS-CoV-2delta_QUD52764.1              SNNSIAIPTNFTISVTTEILPVSMTKTSVDCTMYICGDSTECSNLLLQYGSFCTQLNRAL
SARS-CoV-2_BA1_UFO69279.1               SNNSIAIPTNFTISVTTEILPVSMTKTSVDCTMYICGDSTECSNLLLQYGSFCTQLKRAL
SARS-CoV-2_BA2_UJP23605.1               SNNSIAIPTNFTISVTTEILPVSMTKTSVDCTMYICGDSTECSNLLLQYGSFCTQLKRAL
SARS-CoV-2_BA5_UOZ45804.1               SNNSIAIPTNFTISVTTEILPVSMTKTSVDCTMYICGDSTECSNLLLQYGSFCTQLKRAL
SARS-CoV-2_BQ1.1_UWM38596.1             SNNSIAIPTNFTISVTTEILPVSMTKTSVDCTMYICGDSTECSNLLLQYGSFCTQLKRAL
SARS-CoV-2_EG.5.1_WGM84363.1            SNNSIAIPTNFTISVTTEILPVSMTKTSVDCTMYICGDSTECSNLLLQYGSFCTQLKRAL
SARS-CoV-2_XBB.1.5_UZG29433.1           SNNSIAIPTNFTISVTTEILPVSMTKTSVDCTMYICGDSTECSNLLLQYGSFCTQLKRAL
SARS-CoV-2_JN.1_WPF38074.1              SNNSIAIPTNFTISVTTEILPVSMTKTSVDCTMYICGDSTECSNLLLQYGSFCTQLKRAL
SARS-CoV-2_KP.3.1.1                     SNNSIAIPTNFTISVTTEILPVSMTKTSVDCTMYICGDSTECSNLLLQYGSFCTQLKRAL
Pangolin_GD-consensus_Lam2020           ANNSIAIPTNFTISVTTEILPVSMTKTSVDCTMYICGDSIECSNLLLQYGSFCTQLNRAL
RaTG13_MN996532                         SNNSIAIPTNFTISVTTEILPVSMTKTSVDCTMYICGDSTECSNLLLQYGSFCTQLNRAL
Pangolin_GX-P2V_EPI_ISL_410542          SNNSIAIPTNFTISVTTEILPVSMTKTSVDCTMYICGDSIECSNLLLQYGSFCTQLNRAL
SARS-CoV-1_Urbani_HP03L_AY278741        SNNTIAIPTNFSISITTEVMPVSMAKTSVDCNMYICGDSTECANLLLQYGSFCTQLNRAL
SARS-CoV-1_HGZ8L1-A_HP03E_AY394981      SNNTIAIPTNFSISITTEVMPVSMAKTSVDCNMYICGDSTECANLLLQYGSFCTQLNRAL
SARS-CoV-1_GZ-C_HP03L_AY394979          SNNTIAIPTNFSISITTEVMPVSMAKTSVDCNMYICGDSTECANLLLQYGSFCTQLNRAL
SARS-CoV-1_Sino1-11_HP03L_AY485277      SNNTIAIPTNFSISITTEVMPVSMAKTSVDCNMYICGDSTECANLLLQYGSFCTQLNRAL
SARS-CoV-1_Sin852_HP03L_AY559082        SNNTIAIPTNFSISITTEVMPVSMAKTSVDCNMYICGDSTECANLLLQYGSFCTQLNRAL
SARS-CoV-1_SZ1_PC03_AY304489            SNNTIAIPTNFLISITTEVMPVSMVKTSVDCNMYICGDSTECANLLLQYGSFCAQLNRAL
SARS-CoV-1_GD03T0013_HP04_AY525636      SNNTIAIPTNFSISITTEVMPVSMAKTSVDCNMYICGDSTECANLLLQYGSFCRQLNRAL
SARS-CoV-1_GZ0402_HP04_AY613947         SNNTIAIPTNFSISITTEVMPVSMAKTSVDCNMYICGDSTECANLLLQYGSFCRQLNRAL
SARS-CoV-1_PC4-127_PC04_AY613951        SNNTIAIPTNFSISITTEVMPVSMAKTSVDCNMYICGDSTECANLLLQYGSFCRQLNRAL
SARS-CoV-1_PC4-137_PC04_AY627045        SNNTIAIPTNFSISITTEVMPVSMAKTSVDCNMYICGDSTECANLLLQYGSFCRQLNRAL
SARS-CoV-1_PC4-13_PC04_AY613948         SNNTIAIPTNFSISITTEVMPVSMAKTSVDCNMYICGDSTECANLLLQYGSFCRQLNRAL
WIV1_KF367457                           SNNTIAIPTNFSISITTEVMPVSMAKTSVDCNMYICGDSTECANLLLQYGSFCTQLNRAL
Rs7327_KY417151                         SNNTIAIPTNFSISITTEVMPVSMAKTSVDCNMYICGDSTECANLLLQYGSFCTQLNRAL
Rs4231_KY417146                         SNNTIAIPTNFSISITTEVMPVSMAKTSVDCNMYICGDSTECANLLLQYGSFCTQLNRAL
RsSHC014_KC881005                       SNNTIAIPTNFSISITTEVMPVSMAKTSVDCNMYICGDSTECANLLLQYGSFCTQLNRAL
Rs4084_KY417144                         SNNTIAIPTNFSISITTEVMPVSMAKTSVDCNMYICGDSTECANLLLQYGSFCTQLNRAL
BtKY72_KY352407                         SNNIIAIPTNFSISVTTEVLPVSMTKTSVDCNMYICGDSTECSNLLLQYGSFCTQLNRAL
                                        :** ******* **:***::****.******.******* **:********** **:***

SARS-CoV-2Wuhan_MN908947                TGIAVEQDKNTQEVFAQVKQIYKTPPIKDFGGFNFSQILPDPSKPSKRSFIEDLLFNKVT
SARS-CoV-2delta_QUD52764.1              TGIAVEQDKNTQEVFAQVKQIYKTPPIKDFGGFNFSQILPDPSKPSKRSFIEDLLFNKVT
SARS-CoV-2_BA1_UFO69279.1               TGIAVEQDKNTQEVFAQVKQIYKTPPIKYFGGFNFSQILPDPSKPSKRSFIEDLLFNKVT
SARS-CoV-2_BA2_UJP23605.1               TGIAVEQDKNTQEVFAQVKQIYKTPPIKYFGGFNFSQILPDPSKPSKRSFIEDLLFNKVT
SARS-CoV-2_BA5_UOZ45804.1               TGIAVEQDKNTQEVFAQVKQIYKTPPIKYFGGFNFSQILPDPSKPSKRSFIEDLLFNKVT
SARS-CoV-2_BQ1.1_UWM38596.1             TGIAVEQDKNTQEVFAQVKQIYKTPPIKYFGGFNFSQILPDPSKPSKRSFIEDLLFNKVT
SARS-CoV-2_EG.5.1_WGM84363.1            TGIAVEQDKNTQEVFAQVKQIYKTPPIKYFGGFNFSQILPDPSKPSKRSFIEDLLFNKVT
SARS-CoV-2_XBB.1.5_UZG29433.1           TGIAVEQDKNTQEVFAQVKQIYKTPPIKYFGGFNFSQILPDPSKPSKRSFIEDLLFNKVT
SARS-CoV-2_JN.1_WPF38074.1              TGIAVEQDKNTQEVFAQVKQIYKTPPIKYFGGFNFSQILPDPSKPSKRSFIEDLLFNKVT
SARS-CoV-2_KP.3.1.1                     TGIAVEQDKNTQEVFAQVKQIYKTPPIKYFGGFNFSQILPDPSKPSKRSFIEDLLFNKVT
Pangolin_GD-consensus_Lam2020           TGIAVEQDKNTQEVFAQVKQIYKTPPIKDFGGFNFSQILPDPSKPSKRSFIEDLLFNKVT
RaTG13_MN996532                         TGIAVEQDKNTQEVFAQVKQIYKTPPIKDFGGFNFSQILPDPSKPSKRSFIEDLLFNKVT
Pangolin_GX-P2V_EPI_ISL_410542          TGIAVEQDKNTQEVFAQVKQIYKTPPIKDFGGFNFLQILPDPSKPSKRSFIEDLLFNKVT
SARS-CoV-1_Urbani_HP03L_AY278741        SGIAAEQDRNTREVFAQVKQMYKTPTLKYFGGFNFSQILPDPLKPTKRSFIEDLLFNKVT
SARS-CoV-1_HGZ8L1-A_HP03E_AY394981      SGIAAEQDRNTREVFAQVKQMYKTPTLKDFGGFNFSQILPDPLKPTKRSFIEDLLFNKVT
SARS-CoV-1_GZ-C_HP03L_AY394979          SGIAAEQDRNTREVFAQVKQMYKTPTLKYFGGFNFSQILPDPLKPTKRSFIEDLLFNKVT
SARS-CoV-1_Sino1-11_HP03L_AY485277      SGIAAEQDRNTREVFAQVKQMYKTPTLKYFGGFNFSQILPDPLKPTKRSFIEDLLFNKVT
SARS-CoV-1_Sin852_HP03L_AY559082        SGIAAEQDRNTREVFAQVKQMYKTPTLKYFGGFNFSQILPDPLKPTKRSFIEDLLFNKVT
SARS-CoV-1_SZ1_PC03_AY304489            SGIAVEQDRNTREVFAQVKQMYKTPTLKDFGGFNFSQILPDPLKPTKRSFIEDLLFNKVT
SARS-CoV-1_GD03T0013_HP04_AY525636      SGIAAEQDRNTREVFVQVKQMYKTPTLKDFGGFNFSQILPDPLKPTKRSFIEDLLFNKVT
SARS-CoV-1_GZ0402_HP04_AY613947         SGIAAEQDRNTREVFVQVKQMYKTPTLKDFGGFNFSQILPDPLKPTKRSFIEDLLFNKVT
SARS-CoV-1_PC4-127_PC04_AY613951        SGIAAEQDRNTREVFVQVKQMYKTPTLKDFGGFNFSQILPDPLKPTKRSFIEDLLFNKVT
SARS-CoV-1_PC4-137_PC04_AY627045        SGIAAEQDRNTREVFAQVKQMYKTPTLKDFGGFNFSQILPDPLKPTKRSFIEDLLFNKVT
SARS-CoV-1_PC4-13_PC04_AY613948         SGIAAEQDRNTREVFVQVKQMYKTPTLKDFGGFNFSQILPDPLKPTKRSFIEDLLFNKVT
WIV1_KF367457                           SGIAVEQDRNTREVFAQVKQMYKTPTLKDFGGFNFSQILPDPLKPTKRSFIEDLLFNKVT
Rs7327_KY417151                         SGIAVEQDRNTREVFAQVKQMYKTPTLKDFGGFNFSQILPDPLKPTKRSFIEDLLFNKVT
Rs4231_KY417146                         SGIAVEQDRNTREVFAQVKQMYKTPALKDFGGFNFSQILPDPLKPTKRSFIEDLLFNKVT
RsSHC014_KC881005                       SGIAVEQDRNTREVFAQVKQMYKTPTLKDFGGFNFSQILPDPLKPTKRSFIEDLLFNKVT
Rs4084_KY417144                         SGIAVEQDRNTREVFAQVKQMYKTPTLKDFGGFNFSQILPDPLKPTKRSFIEDLLFNKVT
BtKY72_KY352407                         SGIAVEQDRNTRDVFAQTKSIYKTPNIKDFGGFNFSQILPDPKKLSYRSFIEDLLYNKVT
                                        :***.***:**::**.*.*.:**** :* ****** ****** * : ********:****

SARS-CoV-2Wuhan_MN908947                LADAGFIKQYGDCLGDIAARDLICAQKFNGLTVLPPLLTDEMIAQYTSALLAGTITSGWT
SARS-CoV-2delta_QUD52764.1              LADAGFIKQYGDCLGDIAARDLICAQKFNGLTVLPPLLTDEMIAQYTSALLAGTITSGWT
```

```
SARS-CoV-2_BA1_UFO69279.1              LADAGFIKQYGDCLGDIAARDLICAQKFKGLTVLPPLLTDEMIAQYTSALLAGTITSGWT
SARS-CoV-2_BA2_UJP23605.1              LADAGFIKQYGDCLGDIAARDLICAQKFNGLTVLPPLLTDEMIAQYTSALLAGTITSGWT
SARS-CoV-2_BA5_UOZ45804.1              LADAGFIKQYGDCLGDIAARDLICAQKFNGLTVLPPLLTDEMIAQYTSALLAGTITSGWT
SARS-CoV-2_BQ1.1_UWM38596.1            LADAGFIKQYGDCLGDIAARDLICAQKFNGLTVLPPLLTDEMIAQYTSALLAGTITSGWT
SARS-CoV-2_EG.5.1_WGM84363.1           LADAGFIKQYGDCLGDIAARDLICAQKFNGLTVLPPLLTDEMIAQYTSALLAGTITSGWT
SARS-CoV-2_XBB.1.5_UZG29433.1          LADAGFIKQYGDCLGDIAARDLICAQKFNGLTVLPPLLTDEMIAQYTSALLAGTITSGWT
SARS-CoV-2_JN.1_WPF38074.1             LADAGFIKQYGDCLGDIAARDLICAQKFNGLTVLPPLLTDEMIAQYTSALLAGTITSGWT
SARS-CoV-2_KP.3.1.1                    LADAGFIKQYGDCLGDIAARDLICAQKFNGLTVLPPLLTDEMIAQYTSALLAGTITSGWT
Pangolin_GD-consensus_Lam2020          LADAGFIKQYGDCLGDIAARDLICAQKFNGLTVLPPLLTDEMIAQYTSALLAGTITSGWT
RaTG13_MN996532                        LADAGFIKQYGDCLGDIAARDLICAQKFNGLTVLPPLLTDEMIAQYTSALLAGTITSGWT
Pangolin_GX-P2V_EPI_ISL_410542         LADAGFIKQYGDCLGDIAARDLICAQKFNGLTVLPPLLTDEMIAQYTSALLAGTITSGWT
SARS-CoV-1_Urbani_HP03L_AY278741       LADAGFMKQYGECLGDINARDLICAQKFNGLTVLPPLLTDDMIAAYTAALVSGTATAGWT
SARS-CoV-1_HGZ8L1-A_HP03E_AY394981     LADAGFMKQYGECLGDINARDLICAQKFNGLTVLPPLLTDDMIAAYTAALVSGTATAGWT
SARS-CoV-1_GZ-C_HP03L_AY394979         LADAGFMKQYGECLGDINARDLICAQKFNGLTVLPPLLTDDMIAAYTAALVSGTATAGWT
SARS-CoV-1_Sino1-11_HP03L_AY485277     LADAGFMKQYGECLGDINARDLICAQKFNGLTVLPPLLTDDMIAAYTAALVSGTATAGWT
SARS-CoV-1_Sin852_HP03L_AY559082       LADAGFMKQYGECLGDINARDLICAQKFNGLTVLPPLLTDDMIAAYTAALVSGTATAGWT
SARS-CoV-1_SZ1_PC03_AY304489           LADAGFMKQYGECLGDINARDLICAQKFNGLTVLPPLLTDDMIAAYTAALVSGTATAGWT
SARS-CoV-1_GD03T0013_HP04_AY525636     LADAGFMKQYGECLGDINARDLICAQKFNGLTVLPPLLTDDMIAAYTAALVSGTATAGWT
SARS-CoV-1_GZ0402_HP04_AY613947        LADAGFMKQYGECLGDINARDLICAQKFNGLTVLPPLLTDDMIAAYSAALVSGTATAGWT
SARS-CoV-1_PC4-127_PC04_AY613951       LADAGFMKQYGECLGDINARDLICAQKFNGLTVLPPLLTDDMIAAYTAALVSGTATAGWT
SARS-CoV-1_PC4-137_PC04_AY627045       LADAGFMKQYGECLGDINARDLICAQKFNGLTVLPPLLTDDMIAAYTAALVSGTATAGWT
SARS-CoV-1_PC4-13_PC04_AY613948        LADAGFMKQYGECLGDINARDLICAQKFNGLTVLPPLLTDDMIAAYTAALVSGTATAGWT
WIV1_KF367457                          LADAGFMKQYGECLGDINARDLICAQKFNGLTVLPPLLTDDMIAAYTAALVSGTATAGWT
Rs7327_KY417151                        LADAGFMKQYGECLGDINARDLICAQKFNGLTVLPPLLTDDMIAAYTAALVSGTATAGWT
Rs4231_KY417146                        LADAGFMKQYGECLGDVNARDLICAQKFNGLTVLPPLLTDDMIAAYTAALVSGTATAGWT
RsSHC014_KC881005                      LADAGFMKQYGECLGDINARDLICAQKFNGLTVLPPLLTDDMIAAYTAALVSGTATAGWT
Rs4084_KY417144                        LADAGFMKQYGECLGDINARDLICAQKFNGLTVLPPLLTDDMIAAYTAALVSGTATAGWT
BtKY72_KY352407                        LSDPGFMKQYGDCLGGINARDLICAQKFNGLTVLPPLLTDDMIAAYTAALISGTATAGYT
                                       *:* **:****:***.: **********:***********:*** *::**::** *:*:*

SARS-CoV-2Wuhan_MN908947               FGAGAALQIPFAMQMAYRFNGIGVTQNVLYENQKLIANQFNSAIGKIQDSLSSTASALGK
SARS-CoV-2delta_QUD52764.1             FGAGAALQIPFAMQMAYRFNGIGVTQNVLYENQKLIANQFNSAIGKIQDSLSSTASALGK
SARS-CoV-2_BA1_UFO69279.1              FGAGAALQIPFAMQMAYRFNGIGVTQNVLYENQKLIANQFNSAIGKIQDSLSSTASALGK
SARS-CoV-2_BA2_UJP23605.1              FGAGAALQIPFAMQMAYRFNGIGVTQNVLYENQKLIANQFNSAIGKIQDSLSSTASALGK
SARS-CoV-2_BA5_UOZ45804.1              FGAGAALQIPFAMQMAYRFNGIGVTQNVLYENQKLIANQFNSAIGKIQDSLSSTASALGK
SARS-CoV-2_BQ1.1_UWM38596.1            FGAGAALQIPFAMQMAYRFNGIGVTQNVLYENQKLIANQFNSAIGKIQDSLSSTASALGK
SARS-CoV-2_EG.5.1_WGM84363.1           FGAGAALQIPFAMQMAYRFNGIGVTQNVLYENQKLIANQFNSAIGKIQDSLSSTASALGK
SARS-CoV-2_XBB.1.5_UZG29433.1          FGAGAALQIPFAMQMAYRFNGIGVTQNVLYENQKLIANQFNSAIGKIQDSLSSTASALGK
SARS-CoV-2_JN.1_WPF38074.1             FGAGAALQIPFAMQMAYRFNGIGVTQNVLYENQKLIANQFNSAIGKIQDSLFSTASALGK
SARS-CoV-2_KP.3.1.1                    FGAGAALQIPFAMQMAYRFNGIGVTQNVLYENQKLIANQFNSAIGKIQDSLFSTASALGK
Pangolin_GD-consensus_Lam2020          FGAGAALQIPFAMQMAYRFNGIGVTQNVLYENQKLIANQFNSAIGKIQDSLSSTASALGK
RaTG13_MN996532                        FGAGAALQIPFAMQMAYRFNGIGVTQNVLYENQKLIANQFNSAIGKIQDSLSSTASALGK
Pangolin_GX-P2V_EPI_ISL_410542         FGAGAALQIPFAMQMAYRFNGIGVTQNVLYENQKLIANQFNSAIGKIQDSLSSTASALGK
SARS-CoV-1_Urbani_HP03L_AY278741       FGAGAALQIPFAMQMAYRFNGIGVTQNVLYENQKQIANQFNKAISQIQESLTTTSTALGK
SARS-CoV-1_HGZ8L1-A_HP03E_AY394981     FGAGAALQIPFAMQMAYRFNGIGVTQNVLYENQKQIANQFNKAISQIQESLTTTSTALGK
SARS-CoV-1_GZ-C_HP03L_AY394979         FGAGAALQIPFAMQMAYRFNGIGVTQNVLYENQKQIANQFNKAISQIQESLTTTSTALGK
SARS-CoV-1_Sino1-11_HP03L_AY485277     FGAGAALQIPFAMQMAYRFNGIGVTQNVLYENQKQIANQFNKAISQIQESLTTTSTALGK
SARS-CoV-1_Sin852_HP03L_AY559082       FGAGAALQIPFAMQMAYRFNGIGVTQNVLYENQKQIANQFNKAISQIQESLTTTSTALGK
SARS-CoV-1_SZ1_PC03_AY304489           FGAGAALQIPFAMQMAYRFNGIGVAQNVLYENQKQIANQFNKAISQIQESLTTTSTALGK
SARS-CoV-1_GD03T0013_HP04_AY525636     FGAGAALQIPFAMQMAYRFNGIGVTQNVLYENQKQIANQFNKAISQIQESLTTTSTALGK
SARS-CoV-1_GZ0402_HP04_AY613947        FGAGAALQIPFAMQMAYRFNGIGVTQNVLYENQKQIANQFNKAISQIQESLTTTSTALGK
SARS-CoV-1_PC4-127_PC04_AY613951       FGAGAALQIPFAMQMAYRFNGIGVTQNVLYENQKQIANQFNKAISQIQESLTTTSTALGK
SARS-CoV-1_PC4-137_PC04_AY627045       FGAGAALQIPFAMQMAYRFNGIGVTQNVLYENQKQIANQFNKAISQIQESLTTTSTALGK
SARS-CoV-1_PC4-13_PC04_AY613948        FGAGAALQIPFAMQMAYRFNGIGVTQNVLYENQKQIANQFNKAISQIQESLTTTSTALGK
WIV1_KF367457                          FGAGAALQIPFAMQMAYRFNGIGVTQNVLYENQKQIANQFNKAISQIQESLTTTSTALGK
Rs7327_KY417151                        FGAGAALQIPFAMQMAYRFNGIGVTQNVLYENQKQIANQFNKAISQIQESLTTTSTALGK
Rs4231_KY417146                        FGAGAALQIPFAMQMAYRFNGIGVTQNVLYENQKQIANQFNKAISQIQESLTTTSTALGK
RsSHC014_KC881005                      FGAGAALQIPFAMQMAYRFNGIGVTQNVLYENQKQIANQFNKAISQIQESLTTTSTALGK
Rs4084_KY417144                        FGAGAALQIPFAMQMAYRFNGIGVTQNVLYENQKQIANQFNKAISQIQESLTTTSTALGK
BtKY72_KY352407                        FGAGAALQIPFAMQMAYRFNGIGVTQNVLYENQKQIANQFNNAISKIQDSLTTTSAALGK
                                       ************************:********* ******.**.:**:** :*::****

SARS-CoV-2Wuhan_MN908947               LQDVVNQNAQALNTLVKQLSSNFGAISSVLNDILSRLDKVEAEVQIDRLITGRLQSLQTY
SARS-CoV-2delta_QUD52764.1             LQNVVNQNAQALNTLVKQLSSNFGAISSVLNDILSRLDKVEAEVQIDRLITGRLQSLQTY
SARS-CoV-2_BA1_UFO69279.1              LQDVVNHNAQALNTLVKQLSSKFGAISSVLNDIFSRLDKVEAEVQIDRLITGRLQSLQTY
SARS-CoV-2_BA2_UJP23605.1              LQDVVNHNAQALNTLVKQLSSKFGAISSVLNDILSRLDKVEAEVQIDRLITGRLQSLQTY
SARS-CoV-2_BA5_UOZ45804.1              LQDVVNHNAQALNTLVKQLSSKFGAISSVLNDILSRLDKVEAEVQIDRLITGRLQSLQTY
SARS-CoV-2_BQ1.1_UWM38596.1            LQDVVNHNAQALNTLVKQLSSKFGAISSVLNDILSRLDKVEAEVQIDRLITGRLQSLQTY
SARS-CoV-2_EG.5.1_WGM84363.1           LQDVVNHNAQALNTLVKQLSSKFGAISSVLNDILSRLDKVEAEVQIDRLITGRLQSLQTY
SARS-CoV-2_XBB.1.5_UZG29433.1          LQDVVNHNAQALNTLVKQLSSKFGAISSVLNDILSRLDKVEAEVQIDRLITGRLQSLQTY
SARS-CoV-2_JN.1_WPF38074.1             LQDVVNHNAQALNTLVKQLSSKFGAISSVLNDILSRLDKVEAEVQIDRLITGRLQSLQTY
SARS-CoV-2_KP.3.1.1                    LQDVVNHNAQALNTLVKQLSSKFGAISSVLNDILSRLDKVEAEVQIDRLITGRLQSLQTY
Pangolin_GD-consensus_Lam2020          LQDVVNQNAQALNTLVKQLSSNFGAISSVLNDILSRLDKVEAEVQIDRLITGRLQSLQTY
RaTG13_MN996532                        LQDVVNQNAQALNTLVKQLSSNFGAISSVLNDILSRLDKVEAEVQIDRLITGRLQSLQTY
Pangolin_GX-P2V_EPI_ISL_410542         LQDVVNQNAQALNTLVKQLSSNFGAISSVLNDILSRLDKVEAEVQIDRLITGRLQSLQTY
SARS-CoV-1_Urbani_HP03L_AY278741       LQDVVNQNAQALNTLVKQLSSNFGAISSVLNDILSRLDKVEAEVQIDRLITGRLQSLQTY
```

```
SARS-CoV-1_HGZ8L1-A_HP03E_AY394981      LQDVVNQNAQALNTLVKQLSSNFGAISSVLNDILSRLDKVEAEVQIDRLITGRLQSLQTY
SARS-CoV-1_GZ-C_HP03L_AY394979          LQDVVNQNAQALNTLVKQLSSNFGAISSVLNDILSRLDKVEAEVQIDRLITGRLQSLQTY
SARS-CoV-1_Sino1-11_HP03L_AY485277      LQDVVNQNAQALNTLVKQLSSNFGAISSVLNDILSRLDKVEAEVQIDRLITGRLQSLQTY
SARS-CoV-1_Sin852_HP03L_AY559082        LQDVVNQNAQALNTLVKQLSSNFGAISSVLNDILSRLDKVEAEVQIDRLITGRLQSLQTY
SARS-CoV-1_SZ1_PC03_AY304489            LQDVVNQNAQALNTLVKQLSSNFGAISSVLNDILSRLDKVEAEVQIDRLITGRLQSLQTY
SARS-CoV-1_GD03T0013_HP04_AY525636      LQDVVNQNAQALNTLVKQLSSNFGAISSVLNDILSRLDKVEAEVQIDRLITGRLQSLQTY
SARS-CoV-1_GZ0402_HP04_AY613947         LQDVVNQNAQALNTLVKQLSSNFGAISSVLNDILSRLDKVEAEVQIDRLITGRLQSLQTY
SARS-CoV-1_PC4-127_PC04_AY613951        LQDVVNQNAQALNTLVKQLSSNFGAISSVLNDILSRLDKVEAEVQIDRLITGRLQSLQTY
SARS-CoV-1_PC4-137_PC04_AY627045        LQDVVNQNAQALNTLVKQLSSNFGAISSVLNDILSRLDKVEAEVQIDRLITGRLQSLQTY
SARS-CoV-1_PC4-13_PC04_AY613948         LQDVVNQNAQALNTLVKQLSSNFGAISSVLNDILSRLDKVEAEVQIDRLITGRLQSLQTY
WIV1_KF367457                           LQDVVNQNAQALNTLVKQLSSNFGAISSVLNDILSRLDKVEAEVQIDRLITGRLQSLQTY
Rs7327_KY417151                         LQDVVNQNAQALNTLVKQLSSNFGAISSVLNDILSRLDKVEAEVQIDRLITGRLQSLQTY
Rs4231_KY417146                         LQDVVNQNAQALNTLVKQLSSNFGAISSVLNDILSRLDKVEAEVQIDRLITGRLQSLQTY
RsSHC014_KC881005                       LQDVVNQNAQALNTLVKQLSSNFGAISSVLNDILSRLDKVEAEVQIDRLITGRLQSLQTY
Rs4084_KY417144                         LQDVVNQNAQALNTLVKQLSSNFGAISSVLNDILSRLDKVEAEVQIDRLITGRLQSLQTY
BtKY72_KY352407                         LQDVINQNAVALNTLVKQLSSNFGAISSVLNDILSRLDKVEAEVQIDRLITGRLQSLQTY
                                        **:*:*:** ***********:***********:**************************

SARS-CoV-2Wuhan_MN908947                VTQQLIRAAEIRASANLAATKMSECVLGQSKRVDFCGKGYHLMSFPQSAPHGVVFLHVTY
SARS-CoV-2delta_QUD52764.1              VTQQLIRAAEIRASANLAATKMSECVLGQSKRVDFCGKGYHLMSFPQSAPHGVVFLHVTY
SARS-CoV-2_BA1_UFO69279.1               VTQQLIRAAEIRASANLAATKMSECVLGQSKRVDFCGKGYHLMSFPQSAPHGVVFLHVTY
SARS-CoV-2_BA2_UJP23605.1               VTQQLIRAAEIRASANLAATKMSECVLGQSKRVDFCGKGYHLMSFPQSAPHGVVFLHVTY
SARS-CoV-2_BA5_UOZ45804.1               VTQQLIRAAEIRASANLAATKMSECVLGQSKRVDFCGKGYHLMSFPQSAPHGVVFLHVTY
SARS-CoV-2_BQ1.1_UWM38596.1             VTQQLIRAAEIRASANLAATKMSECVLGQSKRVDFCGKGYHLMSFPQSAPHGVVFLHVTY
SARS-CoV-2_EG.5.1_WGM84363.1            VTQQLIRAAEIRASANLAATKMSECVLGQSKRVDFCGKGYHLMSFPQSAPHGVVFLHVTY
SARS-CoV-2_XBB.1.5_UZG29433.1           VTQQLIRAAEIRASANLAATKMSECVLGQSKRVDFCGKGYHLMSFPQSAPHGVVFLHVTY
SARS-CoV-2_JN.1_WPF38074.1              VTQQLIRAAEIRASANLAATKMSECVLGQSKRVDFCGKGYHLMSFPQSAPHGVVFLHVTY
SARS-CoV-2_KP.3.1.1                     VTQQLIRAAEIRASANLAATKMSECVLGQSKRVDFCGKGYHLMSFPQSAPHGVVFLHVTY
Pangolin_GD-consensus_Lam2020           VTQQLIRAAEIRASANLAATKMSECVLGQSKRVDFCGKGYHLMSFPQSAPHGVVFLHVTY
RaTG13_MN996532                         VTQQLIRAAEIRASANLAATKMSECVLGQSKRVDFCGKGYHLMSFPQSAPHGVVFLHVTY
Pangolin_GX-P2V_EPI_ISL_410542          VTQQLIRAAEIRASANLAATKMSECVLGQSKRVDFCGKGYHLMSFPQSAPHGVVFLHVTY
SARS-CoV-1_Urbani_HP03L_AY278741        VTQQLIRAAEIRASANLAATKMSECVLGQSKRVDFCGKGYHLMSFPQAAPHGVVFLHVTY
SARS-CoV-1_HGZ8L1-A_HP03E_AY394981      VTQQLIRAAEIRASANLAATKMSECVLGQSKRVDFCGKGYHLMSFPQAAPHGVVFLHVTY
SARS-CoV-1_GZ-C_HP03L_AY394979          VTQQLIRAAEIRASANLAATKMSECVLGQSKRVDFCGKGYHLMSFPQAAPHGVVFLHVTY
SARS-CoV-1_Sino1-11_HP03L_AY485277      VTQQLIRAAEIRASANLAATKMSECVLGQSKRVDFCGKGYHLMSFPQAAPHGVVFLHVTY
SARS-CoV-1_Sin852_HP03L_AY559082        VTQQLIRAAEIRASANLAATKMSECVLGQSKRVDFCGKGYHLMSFPQAAPHGVVFLHVTY
SARS-CoV-1_SZ1_PC03_AY304489            VTQQLIRAAEIRASANLAATKMSECVLGQSKRVDFCGKGYHLMSFPQAAPHGVVFLHVTY
SARS-CoV-1_GD03T0013_HP04_AY525636      VTQQLIRAAEIRASANLAATKMSECVLGQSKRVDFCGKGYHLMSFPQAAPHGVVFLHVTY
SARS-CoV-1_GZ0402_HP04_AY613947         VTQQLIRAAEIRASANLAATKMSECVLGQSKRVDFCGKGYHLMSFPQAAPHGVVFLHVTY
SARS-CoV-1_PC4-127_PC04_AY613951        VTQQLIRAAEIRASANLAATKMSECVLGQSKRVDFCGKGYHLMSFPQAAPHGVVFLHVTY
SARS-CoV-1_PC4-137_PC04_AY627045        VTQQLIRAAEIRASANLAATKMSECVLGQSKRVDFCGKGYHLMSFPQAAPHGVVFLHVTY
SARS-CoV-1_PC4-13_PC04_AY613948         VTQQLIRAAEIRASANLAATKMSECVLGQSKRVDFCGKGYHLMSFPQAAPHGVVFLHVTY
WIV1_KF367457                           VTQQLIRAAEIRASANLAATKMSECVLGQSKRVDFCGKGYHLMSFPQAAPHGVVFLHVTY
Rs7327_KY417151                         VTQQLIRAAEIRASANLAATKMSECVLGQSKRVDFCGKGYHLMSFPQAAPHGVVFLHVTY
Rs4231_KY417146                         VTQQLIRAAEIRASANLAATKMSECVLGQSKRVDFCGKGYHLMSFPQAAPHGVVFLHVTC
RsSHC014_KC881005                       VTQQLIRAAEIRASANLAATKMSECVLGQSKRVDFCGKGYHLMSFPQAAPHGVVFLHVTY
Rs4084_KY417144                         VTQQLIRAAEIRASANLAATKMSECVLGQSKRVDFCGKGYHLMSFPQAAPHGVVFLHVTY
BtKY72_KY352407                         VTQQLIRAAEIRASANLAATKMSECVLGQSKRVDFCGKGYHLMSFPQAAPHGVVFLHVTY
                                        ***********************************************:*********** 

SARS-CoV-2Wuhan_MN908947                VPAQEKNFTTAPAICHDGKAHFPREGVFVSNGTHWFVTQRNFYEPQIITTDNTFVSGNCD
SARS-CoV-2delta_QUD52764.1              VPAQEKNFTTAPAICHDGKAHFPREGVFVSNGTHWFVTQRNFYEPQIITTDNTFVSGNCD
SARS-CoV-2_BA1_UFO69279.1               VPAQEKNFTTAPAICHDGKAHFPREGVFVSNGTHWFVTQRNFYEPQIITTDNTFVSGNCD
SARS-CoV-2_BA2_UJP23605.1               VPAQEKNFTTAPAICHDGKAHFPREGVFVSNGTHWFVTQRNFYEPQIITTDNTFVSGNCD
SARS-CoV-2_BA5_UOZ45804.1               VPAQEKNFTTAPAICHDGKAHFPREGVFVSNGTHWFVTQRNFYEPQIITTDNTFVSGNCD
SARS-CoV-2_BQ1.1_UWM38596.1             VPAQEKNFTTAPAICHDGKAHFPREGVFVSNGTHWFVTQRNFYEPQIITTDNTFVSGNCD
SARS-CoV-2_EG.5.1_WGM84363.1            VPAQEKNFTTAPAICHDGKAHFPREGVFVSNGTHWFVTQRNFYEPQIITTDNTFVSGNCD
SARS-CoV-2_XBB.1.5_UZG29433.1           VPAQEKNFTTAPAICHDGKAHFPREGVFVSNGTHWFVTQRNFYEPQIITTDNTFVSGNCD
SARS-CoV-2_JN.1_WPF38074.1              VPAQEKNFTTAPAICHDGKAHFPREGVFVSNGTHWFVTQRNFYEPQIITTDNTFVSGNCD
SARS-CoV-2_KP.3.1.1                     VPAQEKNFTTAPAICHDGKAHFPREGVFVSNGTHWFLTQRNFYEPQIITTDNTFVSGNCD
Pangolin_GD-consensus_Lam2020           VPSQEKNFTTTPAICHEGKAHFPREGVFVSNGTHWFVTQRNFYEPQIITTDNTFVSGSCD
RaTG13_MN996532                         VPAQEKNFTTAPAICHDGKAHFPREGVFVSNGTHWFVTQRNFYEPQIITTDNTFVSGSCD
Pangolin_GX-P2V_EPI_ISL_410542          VPAQEKNFTTAPAICHEGKAHFPREGVFVSNGTHWFITQRNFYEPQIITTDNTFVSGSCD
SARS-CoV-1_Urbani_HP03L_AY278741        VPSQERNFTTAPAICHEGKAYFPREGVFVFNGTSWFITQRNFFSPQIITTDNTFVSGNCD
SARS-CoV-1_HGZ8L1-A_HP03E_AY394981      VPSQERNFTTAPAICHEGKAYFPREGVFVFNGTSWFITQRNFFSPQIITTDNTFVSGNCD
SARS-CoV-1_GZ-C_HP03L_AY394979          VPSQERNFTTAPAICHEGKAYFPREGVFVFNGTSWFITQRNFFSPQIITTDNTFVSGNCD
SARS-CoV-1_Sino1-11_HP03L_AY485277      VPSQERNFTTAPAICHEGKAYFPREGVFVFNGTSWFITQRNFFSPQIITTDNTFVSGNCD
SARS-CoV-1_Sin852_HP03L_AY559082        VPSQERNFTTAPAICHEGKAYFPREGVFVFNGTSWFITQRNFFSPQIITTDNTFVSGNCD
SARS-CoV-1_SZ1_PC03_AY304489            VPSQERNFTTAPAICHEGKAYFPREGVFVFNGTSWFITQRNFFSPQIITTDNTFVSGNCD
SARS-CoV-1_GD03T0013_HP04_AY525636      VPSQERNFTTAPAICHEGKAYFPREGVFVFNGTSWFITQRNFFSPQIITTDNTFVSGNCD
SARS-CoV-1_GZ0402_HP04_AY613947         VPSQERNFTTAPAICHEGKAYFPREGVFVFNGTSWFITQRNFFSPQIITTDNTFVSGNCD
SARS-CoV-1_PC4-127_PC04_AY613951        VPSQERNFTTAPAICHEGKAYFPREGVFVFNGTSWFITQRNFFSPQIITTDNTFVSGNCD
SARS-CoV-1_PC4-137_PC04_AY627045        VPSQERNFTTAPAICHEGKAYFPREGVFVFNGTSWFITQRNFFSPQIITTDNTFVSGNCD
SARS-CoV-1_PC4-13_PC04_AY613948         VPSQERNFTTAPAICHEGKAYFPREGVFVFNGTSWFITQRNFFSPQIITTDNTFVSGNCD
WIV1_KF367457                           VPSQERNFTTAPAICHEGKAYFPREGVFVFNGTSWFITQRNFFSPQIITTDNTFVSGSCD
Rs7327_KY417151                         VPSQERNFTTAPAICHEGKAYFPREGVFVFNGTSWFITQRNFFSPQIITTDNTFVSGNCD
```

```
Rs4231_KY417146                         VPSQERNFTTAPAICHEGKAYFPREGVFVFNGTSWFITQRNFFSPQIITTDNTFVSGSCD
RsSHC014_KC881005                       VPSQERNFTTAPAICHEGKAYFPREGVFVFNGTSWFITQRNFFSPQIITTDNTFVSGSCD
Rs4084_KY417144                         VPSQERNFTTAPAICHEGKAYFPREGVFVFNGTSWFITQRNFFSPQIITTDNTFVSGSCD
BtKY72_KY352407                         VPSQQQNFTTAPAICHNGKAYFPREGVFVMNGTHWFITQRNFYSPQVITTDNTFESGSCD
                                        **:*::****:*****:***:******** *** **:*****:.**:******* **.**

SARS-CoV-2Wuhan_MN908947                VVIGIVNNTVYDPLQPELDSFKEELDKYFKNHTSPDVDLGDISGINASVVNIQKEIDRLN
SARS-CoV-2delta_QUD52764.1              VVIGIVNNTVYDPLQPELDSFKEELDKYFKNHTSPDVDLGDISGINASVVNIQKEIDRLN
SARS-CoV-2_BA1_UFO69279.1               VVIGIVNNTVYDPLQPELDSFKEELDKYFKNHTSPDVDLGDISGINASVVNIQKEIDRLN
SARS-CoV-2_BA2_UJP23605.1               VVIGIVNNTVYDPLQPELDSFKEELDKYFKNHTSPDVDLGDISGINASVVNIQKEIDRLN
SARS-CoV-2_BA5_UOZ45804.1               VVIGIVNNTVYDPLQPELDSFKEELDKYFKNHTSPDVDLGDISGINASVVNIQKEIDRLN
SARS-CoV-2_BQ1.1_UWM38596.1             VVIGIVNNTVYDPLQPELDSFKEELDKYFKNHTSPDVDLGDISGINASVVNIQKEIDRLN
SARS-CoV-2_EG.5.1_WGM84363.1            VVIGIVNNTVYDPLQPELDSFKEELDKYFKNHTSPDVDLGDISGINASVVNIQKEIDRLN
SARS-CoV-2_XBB.1.5_UZG29433.1           VVIGIVNNTVYDPLQPELDSFKEELDKYFKNHTSPDVDLGDISGINASVVNIQKEIDRLN
SARS-CoV-2_JN.1_WPF38074.1              VVIGIVNNTVYDPLQLELDSFKEELDKYFKNHTSPDVDLGDISGINASVVNIQKEIDRLN
SARS-CoV-2_KP.3.1.1                     VVIGIVNNTVYDPLQLELDSFKEELDKYFKNHTSPDVDLGDISGINASVVNIQKEIDRLN
Pangolin_GD-consensus_Lam2020           VVIGIVNNTVYDPLQPELDSFKEELDKYFKNHTSPDVDLGDISGINASVVNIQKEIDRLN
RaTG13_MN996532                         VVIGIVNNTVYDPLQPELDSFKEELDKYFKNHTSPDVDLGDISGINASVVNIQKEIDRLN
Pangolin_GX-P2V_EPI_ISL_410542          VVIGIVNNTVYDPLQPELDSFKEELDKYFKNHTSPDVDLGDISGINASVVNIQKEIDRLN
SARS-CoV-1_Urbani_HP03L_AY278741        VVIGIINNTVYDPLQPELDSFKEELDKYFKNHTSPDVDLGDISGINASVVNIQKEIDRLN
SARS-CoV-1_HGZ8L1-A_HP03E_AY394981      VVIGIINNTVYDPLQPELDSFKEELDKYFKNHTSPDVDLGDISGINASVVNIQKEIDRLN
SARS-CoV-1_GZ-C_HP03L_AY394979          VVIGIINNTVYDPLQPELDSFKEELDKYFKNHTSPDVDLGDISGINASVVNIQKEIDRLN
SARS-CoV-1_Sino1-11_HP03L_AY485277      VVIGIINNTVYDPLQPELDSFKEELDKYFKNHTSPDVDLGDISGINASVVNIQKEIDRLN
SARS-CoV-1_Sin852_HP03L_AY559082        VVIGIINNTVYDPLQPELDSFKEELDKYFKNHTSPDVDLGDISGINASVVNIQKEIDRLN
SARS-CoV-1_SZ1_PC03_AY304489            VVIGIINNTVYDPLQPELDSFKEELDKYFKNHTSPDVDLGDISGINASVVNIQEEIDRLN
SARS-CoV-1_GD03T0013_HP04_AY525636      VVIGIINNTVYDPLQPELDSFKEELDKYFKNHTSPDVDLGDISGINASVVNIQEEIDRLN
SARS-CoV-1_GZ0402_HP04_AY613947         VVIGIINNTVYDPLQPELDSFKEELDKYFKNHTSPDVDLGDISGINASVVNIQEEIDRLN
SARS-CoV-1_PC4-127_PC04_AY613951        VVIGIINNTVYDPLQPELDSFKEELDKYFKNHTSPDVDLGDISGINASVVNIQEEIDRLN
SARS-CoV-1_PC4-137_PC04_AY627045        VVIGIINNTVYDPLQPELDSFKEELDKYFKNHTSPDVDLGDISGINASVVNIQEEIDRLN
SARS-CoV-1_PC4-13_PC04_AY613948         VVIGIINNTVYDPLQPELDSFKEELDKYFKNHTSPDVDLGDISGINASVVNIQEEIDRLN
WIV1_KF367457                           VVIGIINNTVYDPLQPELDSFKEELDKYFKNHTSPDVDLGDISGINASVVNIQKEIDRLN
Rs7327_KY417151                         VVIGIINNTVYDPLQPELDSFKEELDKYFKNHTSPDVDLGDISGINASVVNIQKEIDRLN
Rs4231_KY417146                         VVIGIINNTVYDPLQPELDSFKEELDKYFKNHTSPDVDLGDISGINASVVNIQKEIDRLN
RsSHC014_KC881005                       VVIGIINNTVYDPLQPELDSFKEELDKYFKNHTSPDVDLGDISGINASVVNIQKEIDRLN
Rs4084_KY417144                         VVIGIINNTVYDPLQPELDSFKEELDKYFKNHTSPDVDLGDISGINASVVNIQKEIDRLN
BtKY72_KY352407                         VVIGIINNTVYDPLQPELESFKQELDKYFKNHTSPDVDFGDISGINASVVDIKKEIAHLN
                                        *****:********* **:***:***************:**********:*::** :**

SARS-CoV-2Wuhan_MN908947                EVAKNLNESLIDLQELGKYEQYIKWPWYIWLGFIAGLIAIVMVTIMLCCMTSCCSCLKGC
SARS-CoV-2delta_QUD52764.1              EVAKNLNESLIDLQELGKYEQYIKWPWYIWLGFIAGLIAIVMVTIMLCCMTSCCSCLKGC
SARS-CoV-2_BA1_UFO69279.1               EVAKNLNESLIDLQELGKYEQYIKWPWYIWLGFIAGLIAIVMVTIMLCCMTSCCSCLKGC
SARS-CoV-2_BA2_UJP23605.1               EVAKNLNESLIDLQELGKYEQYIKWPWYIWLGFIAGLIAIVMVTIMLCCMTSCCSCLKGC
SARS-CoV-2_BA5_UOZ45804.1               EVAKNLNESLIDLQELGKYEQYIKWPWYIWLGFIAGLIAIVMVTIMLCCMTSCCSCLKGC
SARS-CoV-2_BQ1.1_UWM38596.1             EVAKNLNESLIDLQELGKYEQYIKWPWYIWLGFIAGLIAIVMVTIMLCCMTSCCSCLKGC
SARS-CoV-2_EG.5.1_WGM84363.1            EVAKNLNESLIDLQELGKYEQYIKWPWYIWLGFIAGLIAIVMVTIMLCCMTSCCSCLKGC
SARS-CoV-2_XBB.1.5_UZG29433.1           EVAKNLNESLIDLQELGKYEQYIKWPWYIWLGFIAGLIAIVMVTIMLCCMTSCCSCLKGC
SARS-CoV-2_JN.1_WPF38074.1              EVAKNLNESLIDLQELGKYEQYIKWPWYIWLGFIAGLIAIVMVTIMLCCMTSCCSCLKGC
SARS-CoV-2_KP.3.1.1                     EVAKNLNESLIDLQELGKYEQYIKWPWYIWLGFIAGLIAIVMVTIMLCCMTSCCSCLKGC
Pangolin_GD-consensus_Lam2020           EVAKNLNESLIDLQELGKYEQYIKWPWYIWLGFIAGLIAIIMVTIMLCCMTSCCSCLKGC
RaTG13_MN996532                         EVAKNLNESLIDLQELGKYEQYIKWPWYIWLGFIAGLIAIIMVTIMLCCMTSCCSCLKGC
Pangolin_GX-P2V_EPI_ISL_410542          EVAKNLNESLIDLQELGKYEQYIKWPWYIWLGFIAGLIAIIMVTIMLCCMTSCCSCLKGC
SARS-CoV-1_Urbani_HP03L_AY278741        EVAKNLNESLIDLQELGKYEQYIKWPWYVWLGFIAGLIAIVMVTILLCCMTSCCSCLKGA
SARS-CoV-1_HGZ8L1-A_HP03E_AY394981      EVAKNLNESFIDLQELGKYEQYIKWPWYVWLGFIAGLIAIVMVTILLCCMTSCCSCLKGA
SARS-CoV-1_GZ-C_HP03L_AY394979          EVAKNLNESLIDLKELGKYEQYIKWPWYVWLGFIAGLIAIVMVTILLCCMTSCCSCLKGA
SARS-CoV-1_Sino1-11_HP03L_AY485277      EVAKNLNESLIDLQELGKYEQYIKWPWYVWLGFIAGLIAIVMVTILLCCMTSCCSCLKGA
SARS-CoV-1_Sin852_HP03L_AY559082        EVAKNLNESLIDLQELGKYEQYIKWPWYVWLGFIAGLIAIVMVTILLCCMTSCCSCLKGA
SARS-CoV-1_SZ1_PC03_AY304489            EVAKNLNESLIDLQELGKYEQYIKWPWYVWLGFIAGLIAIVMVTILLCCMTSCCSCLKGA
SARS-CoV-1_GD03T0013_HP04_AY525636      EVAKNLNESLIDLQELGKYEQYIKWPWYVWLGFIAGLIAIVMVTILLCCMTSCCSCLKGA
SARS-CoV-1_GZ0402_HP04_AY613947         EVAKNLNESLIDLQELGKYEQYIKWPWYVWLGFIAGLIAIVMVTILLCCMTSCCSCLKGA
SARS-CoV-1_PC4-127_PC04_AY613951        EVAKNLNESLIDLQELGKYEQYIKWPWYVWLGFIAGLIAIVMVTILLCCMTSCCSCLKGA
SARS-CoV-1_PC4-137_PC04_AY627045        EVAKNLNESLIDLQELGKYEQYIKWPWYVWLGFIAGLIAIVMVTILLCCMTSCCSCLKGA
SARS-CoV-1_PC4-13_PC04_AY613948         EVAKNLNESLIDLQELGKYEQYIKWPWYVWLGFIAGLIAIVMVTILLCCMTSCCSCLKGA
WIV1_KF367457                           EVAKNLNESLIDLQELGKYEQYIKWPWYVWLGFIAGLIAIVMVTILLCCMTSCCSCLKGA
Rs7327_KY417151                         EVAKNLNESLIDLQELGKYEQYIKWPWYVWLGFIAGLIAIVMVTILLCCMTSCCSCLKGA
Rs4231_KY417146                         EVAKNLNESLIDLQELGKYEQYIKWPWYVWLGFIAGLIAIVMVTILLCCMTSCCSCLKGA
RsSHC014_KC881005                       EVAKNLNESLIDLQELGKYEQYIKWPWYVWLGFIAGLIAIVMVTILLCCMTSCCSCLKGA
Rs4084_KY417144                         EVAKNLNESLIDLQELGKYEQYIKWPWYVWLGFIAGLIAIVMVTILLCCMTSCCSCLKGA
BtKY72_KY352407                         EIAKNLNESLIDLQELGKYEQYVKWPWYVWLGFVAGIVAILMSVIMLCCMTNCCSCFKGM
                                        *:*******:***:********:*****:****:**::**:* .*:*****.****:**

SARS-CoV-2Wuhan_MN908947                CSCGSCCKFDEDDSEPVLKGVKLHYT
SARS-CoV-2delta_QUD52764.1              CSCGSCCKFDEDDSEPVLKGVKLHYT
SARS-CoV-2_BA1_UFO69279.1               CSCGSCCKFDEDDSEPVLKGVKLHYT
SARS-CoV-2_BA2_UJP23605.1               CSCGSCCKFDEDDSEPVLKGVKLHYT
SARS-CoV-2_BA5_UOZ45804.1               CSCGSCCKFDEDDSEPVLKGVKLHYT
SARS-CoV-2_BQ1.1_UWM38596.1             CSCGSCCKFDEDDSEPVLKGVKLHYT
```

```
SARS-CoV-2_EG.5.1_WGM84363.1          CSCGSCCKFDEDDSEPVLKGVKLHYT
SARS-CoV-2_XBB.1.5_UZG29433.1         CSCGSCCKFDEDDSEPVLKGVKLHYT
SARS-CoV-2_JN.1_WPF38074.1            CSCGSCCKFDEDDSEPVLKGVKLHYT
SARS-CoV-2_KP.3.1.1                   CSCGSCCKFDEDDSEPVLKGVKLHYT
Pangolin_GD-consensus_Lam2020         CSCGSCCKFDEDDSEPVLKGVKLHYT
RaTG13_MN996532                       CSCGSCCKFDEDDSEPVLKGVKLHYT
Pangolin_GX-P2V_EPI_ISL_410542        CSCGSCCKFDEDDSEPVLKGVKLHYT
SARS-CoV-1_Urbani_HP03L_AY278741      CSCGSCCKFDEDDSEPVLKGVKLHYT
SARS-CoV-1_HGZ8L1-A_HP03E_AY394981    CSCGSCCKFDEDDSEPVLKGVKLHYT
SARS-CoV-1_GZ-C_HP03L_AY394979        CSCGSCCKFDEDDSEPVLKGVKLHYT
SARS-CoV-1_Sino1-11_HP03L_AY485277    CSCGSCCKFDEDDSEPVLKGVKLHYT
SARS-CoV-1_Sin852_HP03L_AY559082      CSCGSCCKFDEDDSEPVLKGVKLHYT
SARS-CoV-1_SZ1_PC03_AY304489          CSCGSCCKFDEDDSEPVLKGVKLHYT
SARS-CoV-1_GD03T0013_HP04_AY525636    CSCGSCCKFDEDDSEPVLKGVKLHYT
SARS-CoV-1_GZ0402_HP04_AY613947       CSCGSCCKFDEDDSEPVLKGVKLHYT
SARS-CoV-1_PC4-127_PC04_AY613951      CSCGSCCKFDEDDSEPVLKGVKLHYT
SARS-CoV-1_PC4-137_PC04_AY627045      CSCGSCCKFDEDDSEPVLKGVKLHYT
SARS-CoV-1_PC4-13_PC04_AY613948       CSCGSCCKFDEDDSEPVLKGVKLHYT
WIV1_KF367457                         CSCGSCCKFDEDDSEPVLKGVKLHYT
Rs7327_KY417151                       CSCGSCCKFDEDDSEPVLKGVKLHYT
Rs4231_KY417146                       CSCGSCCKFDEDDSEPVLKGVKLHYT
RsSHC014_KC881005                     CSCGSCCKFDEDDSEPVLKGVKLHYT
Rs4084_KY417144                       CSCGSCCKFDEDDSEPVLKGVKLHYT
BtKY72_KY352407                       CSCGSCCKFDEDHSEPV-SGVKLHYT
                                      ************.**** .*******
```
